# Raltegravir Non-Inferior to Nucleoside Based Regimens in SECOND-LINE Therapy with Lopinavir/Ritonavir over 96 Weeks: A Randomised Open Label Study for the Treatment Of HIV-1 Infection

**DOI:** 10.1371/journal.pone.0118228

**Published:** 2015-02-27

**Authors:** Janaki Amin, Mark A. Boyd, Nagalingeswaran Kumarasamy, Cecilia L. Moore, Marcello H. Losso, Chidi A. Nwizu, Lerato Mohapi, Stephen J. Kerr, Annette H. Sohn, Hedy Teppler, Boris Renjifo, Jean-Michel Molina, Sean Emery, David A. Cooper

**Affiliations:** 1 The Kirby Institute, UNSW Australia, Sydney, Australia; 2 Y. R. Gaitonde Centre for AIDS Research and Education, Chennai, India; 3 Hospital JM Ramos Mejia, Buenos Aires, Argentina; 4 Institute of Human Virology, University of Maryland School of Medicine, Baltimore, United States of America; 5 University of the Witwatersrand and Baragwanath Hospital, Johannesburg, South Africa; 6 UNSW Australia, The HIV Netherlands Australia Thailand Research Collaboration, Bangkok, Thailand; 7 TREAT Asia, amfAR, The Foundation for AIDS Research, Bangkok, Thailand; 8 Merck Research Laboratories, Merck Sharp & Dohme, Whitehouse Station, New Jersey, United States of America; 9 Global Medical Affairs Virology, Abbvie, North Chicago, Illinois, United States of America; 10 Service des Maladies Infectieuses, Hôpital Saint Louis, Assistance Publique-Hôpitaux de Paris, Paris, France; Imperial College London, UNITED KINGDOM

## Abstract

**Objective:**

To determine the durability over 96 weeks of safety and efficacy of lopinavir/ritonavir (LPV/r) and raltegravir (RAL) which was demonstrated to have non-inferior efficacy relative to a regimen of LPV/r with nucleoside/nucleotide reverse transcriptase inhibitors (N(t)RTIs) (Control) in primary analysis at 48 weeks.

**Design:**

Open label, centrally randomised trial.

**Setting:**

Recruitment was from 37 primary and secondary care sites from Africa, Asia, Australia, Europe and Latin America.

**Subjects:**

541 HIV-1 infected adults virologically failing first-line non-NRTI + 2N(t)RTI, with no previous exposure to protease inhibitors or integrase strand transfer inhibitors were analysed, 425 completed 96 weeks follow up on randomised therapy.

**Intervention:**

Randomisation was 1:1 to Control or RAL.

**Main outcome measures:**

Differences between the proportion of participants with plasma HIV-1 RNA (VL) <200 copies/mL by intention to treat were compared with a non-inferiority margin of −12%. Differences in biochemical, haematological and metabolic changes were assessed using T-tests.

**Results:**

VL <200 copies/mL at 96 weeks was: RAL 80.4%, Control 76.0% (difference: 4.4 [95%CI −2.6, 11.3]) and met non-inferiority criteria. The RAL arm had a significantly higher mean change (difference Control-RAL; 95%CI) in haemoglobin (−2.9; −5.7, −1.1), total lymphocytes (−0.2; −0.3, −0.0), total cholesterol (−0.5; −0.8, −0.3), HDL cholesterol (−0.1; −0.1, −0.0) and LDL cholesterol (−0.3; −0.5, −0.2).

**Conclusion:**

At 96 weeks, both RAL and Control maintained efficacy greater than 75% and continued to demonstrate similar safety profiles. These results support the use of a combination LPV/r and RAL regimen as an option following failure of 1st line NNRTI + 2N(t)RTIs.

**Trial Registration:**

ClinicalTrials.gov NCT00931463

## Introduction

An estimated 9.7 million people in low- and middle-income countries are now receiving antiretroviral therapy (ART) with the number of people receiving therapy globally tripling over the last five years [1]. With this increase in the numbers of people initiating therapy, so too there is inevitably an increase in the number failing first-line therapy. The choice and rationale for selection of second-line therapy is limited.

A recent Cochrane review suggests that there is little evidence to inform treatment guidelines for second-line therapy recommendations [2]. Within this setting of an acknowledged dearth of information, the World Health Organization (WHO) recommends a boosted-protease inhibitor plus two nucleoside analogues (N(t)RTIs) as the first option for second-line therapy [1]. This approach maximises the use of more commonly available combination antiretroviral therapies (cART); however it does not address the need for simple and tolerable regimens in order to maintain long term suppressive therapy.

The Week 48 report of the SECOND-LINE trial and the Week 96 results of the EARNEST study have reported outcomes of randomised trials that examined the safety and efficacy of experimental regimens of cART for treatment of people who were failing first line therapy[3,4]. Both studies demonstrated non-inferior outcomes for an experimental regimen of cART containing raltegravir plus ritonavir-boosted lopinavir (LPV/r), a very simple regimen, compared to 2–3N(t)RTI + LPV/r.

Here we report the 96 week follow-up data from the SECOND-LINE trial in order to assess the durability of treatment effects in terms of safety and efficacy of the randomised treatment groups.

## Methods

The protocol for this trial and supporting CONSORT checklist are available as supporting information; see S1 Protocol and S1 CONSORT Checklist.

A full description of the methods for the SECOND-LINE trial has been previously published [3]. In summary, SECOND-LINE was a parallel group, randomised, open-label multi-centre international trial conducted at 37 sites across Africa, Asia, Central and South America, Australia and Europe.

Eligible participants were adults (≥16 years or as per local legal requirements) positive for HIV- 1 antibody, failing first-line therapy (non-nucleoside reverse transcriptase inhibitor [NNRTI] plus two nucleoside/nucleotide reverse transcriptase inhibitors [N(t)RTIs]). Failure was defined as two consecutive (>7 days apart) plasma HIV-1 RNA (VL) measures of >500 copies/mL. Patients were excluded if they had prior exposure to a protease inhibitor, an integrase strand transfer inhibitor or had evidence of active viral hepatitis B infection (defined as the presence of HBV surface antigen in serum) [3].

Participants were randomised to one of two antiretroviral regimens containing a total daily dose of ritonavir (200 mg)-boosted lopinavir (800 mg) (LPV/r) (administered either once or twice daily) plus either: i) two or three NtRTIs (Control); or ii) raltegravir 400 mg twice daily (RAL). The dosing schedule (once or twice daily) for LPV/r was determined by the treating physician. Trial visits were scheduled at Weeks 0 (randomisation) 4, 8, 12 and then every 12 weeks to Week 96. At each visit vital signs, physical health and adverse events were assessed and blood was collected for T-cell subset counts, haematology, serum biochemistry including renal function markers, liver function tests, metabolic markers including cholesterol fractions (Weeks 0, 24, 48, 72 and 96 only) and plasma viral load (VL) assessment. Local assessment of VL was used for patient management. Centrally analysed VL was used for assessment of efficacy. Serum collected after fasting was used for assessment of metabolic measures. Estimated glomerular filtration rate (eGFR) was calculated using the modification of diet in renal disease (MDRD) equation [5]. Adherence was measured at Weeks 4, 48 and 96 using the Adult AIDS Clinical Trials Group adherence instrument [6].

### Outcomes

All outcomes in this report were assessed at Week 96. The primary endpoint of this analysis was the proportion of participants with VL <200 copies/mL at 96 weeks. Secondary endpoints were proportion with VL <50 copies/mL and <400 copies/mL, time to loss of virological response and mean change from baseline in CD4+ T-cell count/mm^3^. Virologic failure was also assessed and defined as a VL >200 copies/mL at any time during the trial. Clinical events (adverse events, AIDS, serious non-AIDS [SNAEs], and death), mean changes in laboratory measures and adherence were also assessed. SNAEs were independently reviewed by a physician at the Kirby Institute (Sydney, Australia) according to INSIGHT criteria [7]. Ten-year risk of coronary heart disease (CHD) was determined according to the Framingham equation and metabolic syndrome according to National Cholesterol Education Program Adult Treatment Panel III [8,9]. Both these composite endpoints were only calculated for individuals who had all data components available. There were no imputations for missing data.

### Sample size

The sample size was based on estimates expected at Week 48 of no difference between arms and a predicted efficacy of 80% (VL <200 copies/mL). Two hundred and forty eight participants per arm gave 90% power to show non-inferiority using a margin of 12% (two sided α = 0.05) of the RAL arm compared to the Control arm in the modified intention to treat analysis. The recruited sample size was inflated to allow for adequate power in the Week 48 per protocol population, resulting in 541 participants being randomly assigned. This population will retain 90% power to detect non- inferiority (margin of 12%, two sided α = 0.05) if efficacy over 96 week above 71.5% is maintained.

### Randomisation

Treatment arm allocation, stratified by clinical site and VL (≤ vs > 100,000 copies/mL), was automated with randomisation lists built in to the electronic clinical record form. Randomisation was 1:1 with a blocking factor of four. Allocation could only be triggered by the investigator after completion of participant consent and entry of screening and eligibility data. After random allocation, participants and investigators were not masked to treatment arm allocation.

### Statistical analysis

All binary virologic endpoints were assessed in three analytical populations: i) a modified intention to treat (ITT) population consisting of all randomised participants who commenced study drug and attended at least one follow-up visit- in this population deaths, loss to follow-up and treatment change due to virologic failure were imputed as failures; ii) a per protocol (PP); and iii) a non-completer imputed as failure (NC = F), equivalent to the FDA snapshot analysis [10].

Change in CD4+ T cells/mm^3^ was assessed in the ITT population (last observation carried forward). For other variables, summaries are provided of available data with no imputation.

All analyses were pre-specified. VL threshold analyses were assessed for non-inferiority on the basis of 95% confidence intervals (CI) at a pre-specified margin of the lower confidence bound of −12%. All other endpoints were tested for superiority (two side α = 0.05). The Cochran-Mantel-Haenszel test was used to check for consistency of associations between treatment arms and virological outcomes across screening VL strata.

We used χ^2^ and Fisher’s exact tests to analyse categorical variables; t-test for continuous variables; and Cox regression for time-to-event outcomes and calculation of hazard ratios (HR). Assessment of residual showed no evidence of violation of proportional hazards assumptions. Logistic regression was used to test for interaction of categorical variables. SAS version 9.2 was used for data analysis.

The study was approved by each site’s Ethics Committee under the auspices and approval of the lead ethics/IRB of St Vincent's Hospital Sydney (Reference HREC/09/SVH/89). All participants provided direct written informed consent by signing the Participant Information and Consent Form approved by the IRB. Where a participant was considered a minor written informed consent was obtained from both the participant and their parent/guardian, the ethics committees approved this procedure. Hard copies of the consent form are filed at the participant's relevant clinical site and a copy was given to each participant.

## Results

**Fig 1:**
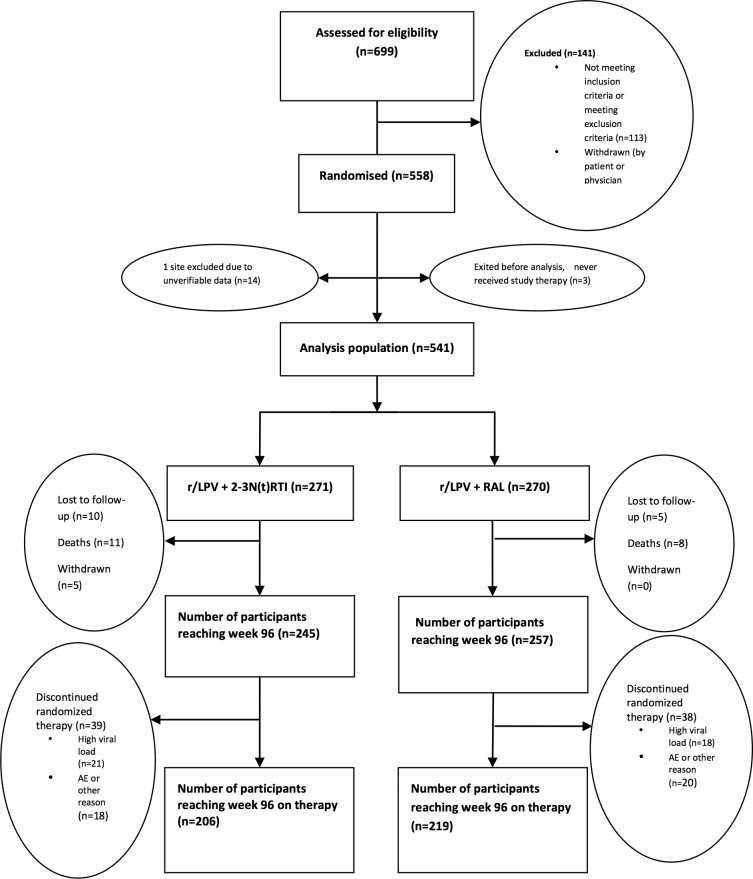
Participant disposition over 96 weeks.

The study commenced recruitment in March 2010 with last participant through 96 weeks of follow up in July 2013. The baseline characteristics of the study population were balanced between arms [3]. Participants were 55% male, 42% Asian, 36% African and 14% Hispanic, with a mean (sd) age of 39 (8.8) years, duration of first line ART was 4.1 (2.8) years, VL was 4.2 (0.9) log copies/mL, CD4+ T cell count was 211 (157) cells/mm^3^. Of the study population randomised to the Control and RAL arms respectively, 245/271(90%) and 257/270(95%) reached 96 weeks of follow up, of whom 206 (84%) and 219 (85%) were on randomised therapy (Fig. 1). At Week 96 there was no difference between arms in self-reported adherence with more than 85% of participants in each arm reporting taking “All” or “Most” of their pills.

**Fig 2:**
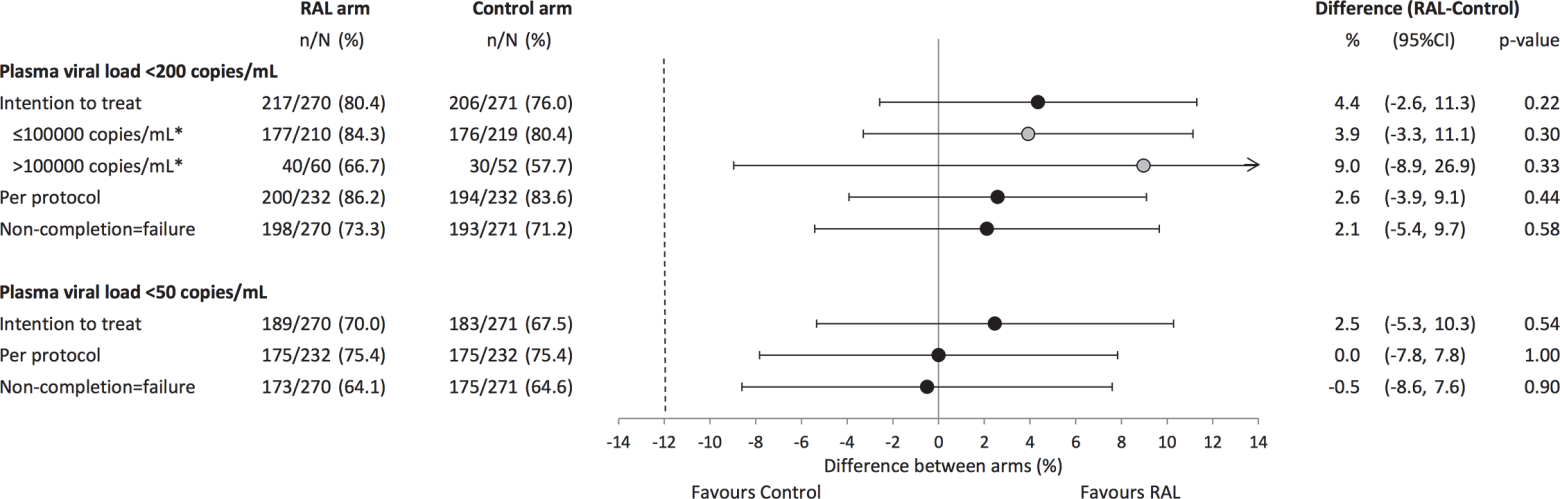
Virologic response at Week 96, by randomised arm, study population and screening strata. RAL = lopinavir/ritonavir+raltegravir Control = lopinavir/ritonavir+2/3 nucleoside/nucleotide reverse transcriptase inhibitors. *grey circle = screening plasma viral load strata, P interaction = 0.81, bars are 95%confidence intervals (CI).

At Week 96, 76% (206/271) in the Control and 80% (217/270) in the RAL arm reported a VL <200 copies /mL (difference RAL-Control = 4.4%, 95%CI −2.6, 11.3) thereby satisfying our *a priori* criteria for non-inferiority. Results were consistent in the PP and NC = F populations, <50 and <400 copy thresholds, and by screening VL strata (Fig. 2). Time to loss of virologic response did not differ between arms (Control 26.9/100 person years (py), RAL 28.1/100 py; HR 1.1 [95% CI 0.8, 1.4]; p = 0.68). Over the first 48 weeks of the trial 55 participants in the Control group and 49 in the RAL group had experienced virologic failure; this increased to 82 and 83 respectively over the entire 96 weeks of follow-up. The frequency of emergent resistance among those who failed with an amplifiable sequence, in the Control and RAL arm, was as follows: NtRTI 8/64 (12.5%) and 2/65 (3.1%); protease inhibitor 2/64 (3.1%) and 1/65 (1.5%); integrase strand inhibitor 1/72 (1.4%) and 20/79 (25.3%).

**Fig 3:**
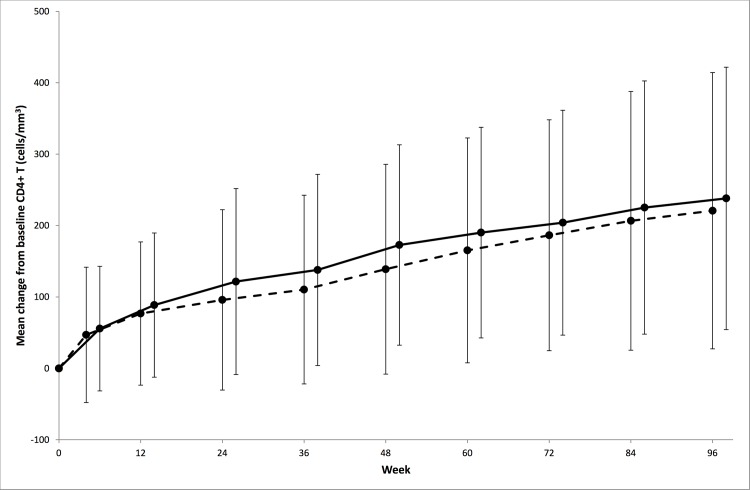
Mean change from baseline in CD4+ T cells/mm^3^ over 96 weeks by randomised arm. RAL = lopinavir/ritonavir+raltegravir Control = lopinavir/ritonavir+2/3 nucleoside/nucleotide reverse transcriptase inhibitors. (Control- - - RAL ---, bars indicate standard deviation).

CD4+ T cells/mm^3^ increased over the whole 96 weeks in both arms (mean change from baseline [95%CI]: 201.7 [178.6, 224.9] and 228.7 [206.6, 250.8] respectively) (Fig. 3). There was no significant difference between arms in mean change from baseline to Week 96 (Control-RAL −27.0 95%CI −59.0, 5.0; p = 0.10).

**Fig 4:**
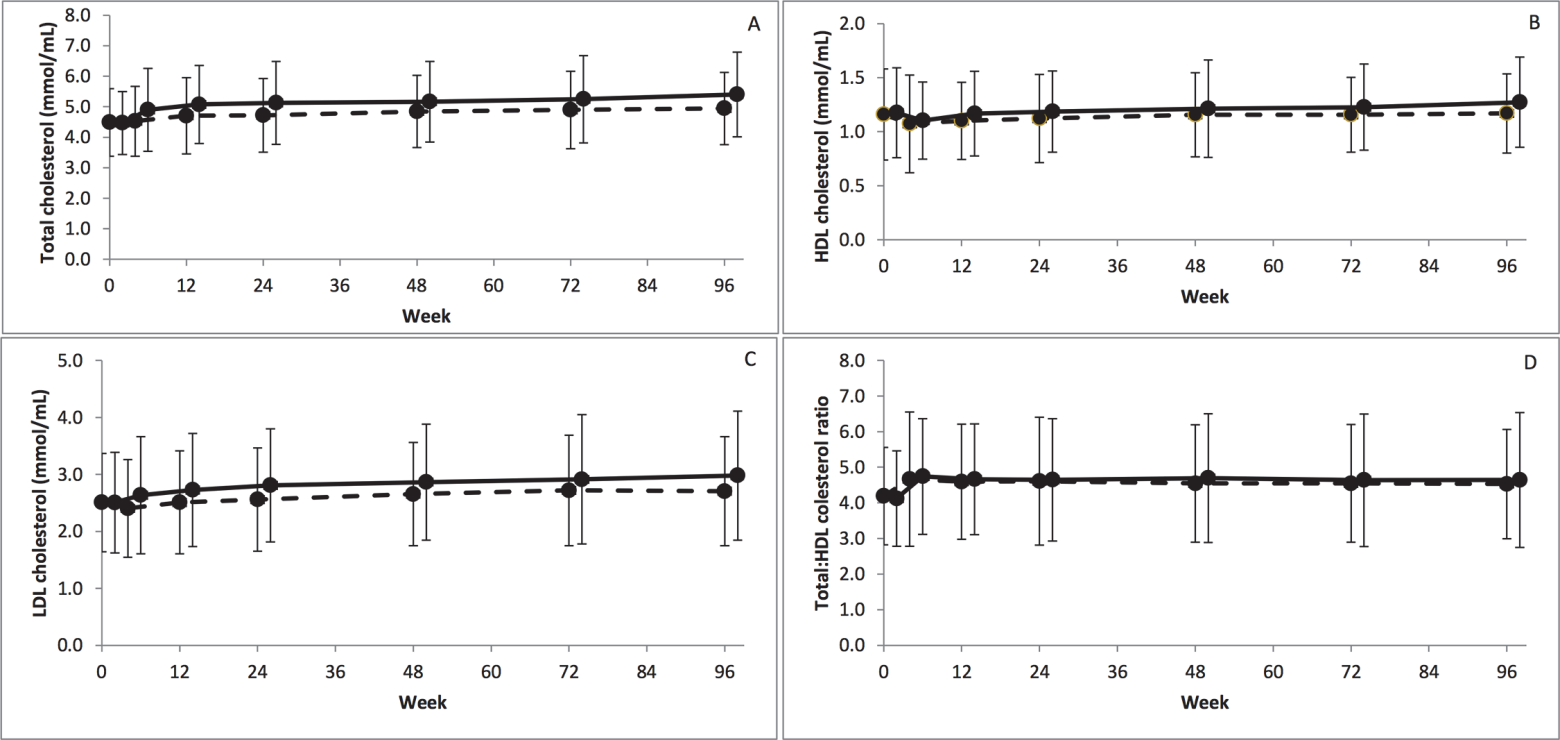
Absolute mean cholesterol fractions and ratio over 96 weeks by randomised arm. (A) total cholesterol, (B) high density lipoprotein (HDL) cholesterol, (C) low density lipoprotein (LDL) cholesterol, (D) Total:HDL cholesterol ratio. RAL = lopinavir/ritonavir+raltegravir Control = lopinavir/ritonavir+2/3 nucleoside/nucleotide reverse transcriptase inhibitors. (Control - - - RAL ---, bars indicate standard deviation).

The differences between arms for mean change from baseline to Week 96 were statistically significant, with greater increases in the RAL arm, for total lymphocytes, haemoglobin, total HDL and LDL cholesterol (Table 1). Mean total cholesterol after 96 weeks of therapy was 4.9 (1.2) in the Control arm and 5.4 (1.4) in the RAL arm, with total:HDL cholesterol ratio increasing up to week 4 (4.7 in both arms) before stabilizing through 96 weeks (Fig. 4). There was a significant difference for mean change from baseline in ten-year CHD risk between arms (mean [sd] baseline Control: 4.1 [4.7], RAL: 3.8 [4.5]) to Week 96 (Control: 4.4[4.4], RAL: 4.6 [5.1]; difference in change −0.6 95%CI −1.2, −0.1; p = 0.02).

**Table 1:** Differences in mean change from baseline to Week 96 in laboratory markers by randomised arm.

	Control		RAL		Difference (Control-RAL)		
	Mean	SD	Mean	SD		95%CI	p-value
*Immunology and haematology*							
CD4+ T (cells/mm^3^)	201.7	193.7	228.7	184.6	−27.0	(−59.0, 5.0)	0.100
Total lymphocytes (cells/mm^3^)	0.5	0.7	0.7	0.7	−0.2	(−0.3, −0.0)	0.018
Neutrophils(cells/mm3)	0.8	1.4	0.9	1.4	−0.1	(−0.3, 0.2)	0.452
Haemoglobin (g/L)	5.5	17.2	8.3	14.7	−2.9	(−5.7, −0.1)	0.046
*Biochemistry*							
ALT (U/L)	−11.1	25.4	−6.6	32.9	−4.5	(−9.7, 0.7)	0.091
Creatine kinase (U/L)	−13.7	203.3	−14.9	193.1	1.3	(−33.8, 36.4)	0.943
eGFR (mL/min/1.73m^2^)	−5.5	16.5	−5.2	14.7	−0.3	(−3.1, 2.5)	0.834
*Metabolic parameters*							
Total cholesterol (mmol/L)	0.4	1.2	0.9	1.2	−0.5	(−0.8, −0.3)	<0.001
HDL choletersol (mmol/L)	0.0	0.4	0.1	0.4	−0.1	(−0.1, −0.0)	0.003
Total:HDL cholesteol ratio	0.4	1.3	0.5	1.5	−0.2	(−0.4, 0.1)	0.204
LDL cholesterol (mmol/L)	0.2	0.8	0.5	0.9	−0.3	(−0.5, −0.2)	<0.001
Triglycerides (mmol/L)	0.8	1.9	1.0	1.8	−0.1	(−0.5, 0.2)	0.417
*Glycaemic parameters*							
Glucose (mmol/L)	−0.1	1.4	0.2	1.8	−0.3	(−0.5, 0.0)	0.090
Insulin (mU/L)	−0.9	16.3	0.6	14.4	−1.6	(−4.3, 1.2)	0.267
HOMA	−0.3	5.5	0.4	4.6	−0.7	(−1.6, 0.2)	0.128

eGFR: estimated glomerular filtration rate, LDL: low density lipoprotein, HDL: high density lipoprotein, HOMA: homeostasis model assessment

There were no significant differences in any biochemistry, including eGFR (mean difference −0.3 95%CI −3.1, 2.5; p = 0.834), glycaemic parameters (Table 1), nor in the proportion developing metabolic syndrome (Control: 22/238, RAL 34/253; difference −0.0% 95%CI−0.1, 0.0; p = 0.17).

There were no differences between arms in rates of individuals ever reporting an adverse event AIDS or death (Table 2). Of the 11 (Control) and 8 (RAL) deaths, 3 and 4 respectively occurred in the last 48 weeks of follow up. A total of 66 AIDS events were reported by 53 individuals. The most commonly occurring AIDS event was tuberculosis (Control 16/30, RAL 18/36) followed by *Pneumocystis jirovecii* pneumonia (Control 2/30, RAL 4/36). Serious non-AIDS events were reported by three participants in the Control arm and four in the RAL arm. Having any clinical event (AIDS, SNAE or death) was associated with never achieving virologic suppression (Odds ratio 5.8 95%CI 3.2–10.8; p = <0.001).

**Table 2:** Clinical outcomes by randomised arm.

	Control (N = 271)		RAL (N = 270)				
	n	Rate/100 py	n	Rate/100 py	Hazard Ratio	95%CI	p-value
Deaths	11	2.4	8	1.6	0.7	(0.3, 1.8)	0.456
AIDS or death	29	6.6	34	7.6	1.2	(0.7, 1.9)	0.538
Serious adverse events	33	7.4	40	8.8	1.2	(0.8, 1.9)	0.456
Adverse events	1391	297.2	1387	285.0	0.9	(0.8, 1.1)	0.238
Number of participants reporting Grade 3/4 adverse events*	*n*	%	*n*	%			
*Number of Grade 3/4 adverse events*							
Total	45	16.6	36	13.3			
	*55*		*46*				
Infections and infestations	11	4.1	7	2.6			
	*12*		*13*				
General disorders and administration site conditions	4	1.5	5	1.9			
	*4*		*5*				
Gastrointestinal disorders	7	2.6	1	0.4			
	*9*		*1*				
Blood and lymphatic system disorders	4	1.5	3	1.1			
	*10*		*3*				
Metabolism and nutrition disorders	2	0.7	3	1.1			
	*2*		*5*				
Pregnancy, puerperium and perinatal conditions	2	0.7	3	1.1			
	*2*		*4*				
Ceased antiretroviral therapy due to any grade adverse event	12/46	26.1	9/45	20.0			

*Grade 3/4 adverse events summarised by MedDRA System Organ Class which were reported by 5 or more people

py: person years

Grade 3/4 events were reported by 45/271 (17%) and 36/270 (13%) (p = 0.29) of individuals in the Control and RAL arms respectively (Table 2). The greatest disparity between arms for grade 3/4 events as summarized by system organ class was for gastrointestinal disorders which were reported by 7/271 (3%) and 1/270 (<0.1%) individuals in each arm respectively. Of the grade 3/4 adverse events 8/55 in the Control arm and 13/46 in the RAL arm were attributed as being possibly, probably or definitely related to study drugs. There was no difference in the proportion of participants reporting adverse events associated with skeletal muscle toxicity (Control 49/271, RAL 61/270; difference −4.5%, 95%CI −11.3, 2.13; p = 0.19). Similar proportions of participants reported ceasing antiretroviral therapy due to an adverse event (Control 12/46, RAL 9/45).

## Discussion

We have shown that over 96 weeks participants who have previously failed first line ART consisting of NNRTI + 2 N(t)RTIs and then commence a regimen of LPV/r with RAL or LPV/r + 2–3N(t)RTI, achieve and maintain VL suppression rates greater than 75%. This finding, as it relates to the N(t)RTI-containing arm, supports therapy guidelines as stated by the WHO. We also demonstrate that a regimen containing LPV/r + RAL provides a non-inferior alternative to currently recommended therapy in a second-line setting [1].

Our study was conducted in diverse settings across high, middle and low income countries, with a high proportion of females and included homosexual and heterosexual participants. Thus the findings from our study are likely to be highly generalizable across similar populations. While our study was not blinded, the primary endpoint, VL, was objective and centrally analysed and thus unlikely to be subject to biased assessment. Randomisation was also central and electronic and the well-balanced characteristics between arms suggests that allocation was random and there is no indication of confounding from measured covariates [3]. However, it is possible that patient management or participant reporting of self-assessed outcomes may have been influenced by the open label nature of this trial.

At Week 48, a statistically significant difference in mean change from baseline CD4+ T-cells/mm^3^ was noted between treatment arms [3], however this difference abated by 96 weeks. This finding may have resulted from sicker people leaving the trial. Alternatively there may have been a further improvement in patient health, with both arms showing a greater than 50 cell/mm^3^ increase in the latter 48 weeks of the trial.

Both trial regimens were well tolerated with the vast majority of participants staying in follow-up, on randomised therapy and adherent. Comparable tolerance is supported by the equal frequency of virologic failure in each arm. The predictors of failure and the role of emergent resistance, particularly integrase strand inhibitor resistance in the RAL arm, require further investigation. In the Control arm, mean change in haemoglobin and total lymphocyte count were significantly lower than in the RAL arm. Associations between thymidine-containing regimens and haematological changes have previously been reported [11], we therefore conducted a pre-specified exploratory analysis restricted to those not on zidovudine-containing regimens in the Control arm compared to the RAL arm. This analysis showed substantial attenuation of the difference in change from baseline in haemoglobin and lymphocytes, such that there was no significant difference between groups. This finding should, however, be interpreted with caution as it is not a true randomised comparison. We found no decrement in the Control arm in terms of eGFR. 253 of 271 (93%) of participants in the Control arm were tenofovir containing regimens, which have been found to result in greater decreases in cohort studies and some but not all switch studies [12–15].

The elevations in total LDL and HDL cholesterol in the RAL arm reported here are consistent with the findings reported at Week 48. It appears that total cholesterol in the RAL arm may be continuing to marginally increase at a higher rate than the Control arm. This may be partially due to high exposure to tenofovir in the Control arm which has been shown to have a beneficial impact on lipids [3,16]. However these findings occur in the presence of no difference between arms in total:HDL ratio. How the lipid profiles translate to clinical risk is difficult to ascertain. The mean total cholesterol in the RAL population (5.4mmol/L) is within the moderate risk estimation for an individual in the general population [8]. While those in the RAL arm were at significantly greater risk for CHD than in the Control arm, their mean absolute ten year risk at the end of study was 4.6%. Recent guidelines, in contrast to Framingham, are also moving away from threshold based assessment of risk to a more holistic individual based risk assessment [8,17]. Poorer lipid outcomes have not been reported in other extended follow up randomised trials of RAL containing regimens [18,19].

After 96 weeks of follow up, our study showed both continued efficacy of the RAL-containing regimen, and non-inferiority compared to Control, in the treatment of HIV following first-line failure. This outcome and the overall adverse events profile are consistent with the preliminary 96 week data from comparative arms in the EARNEST trial[4]. While EARNEST was set in low income countries and SECOND LINE in predominantly middle income countries, both trials demonstrate non-inferior efficacy of a simple RAL based therapy with high tolerability. Given the broad setting of these studies the results bode well for the effectiveness of Control and RAL regimens. However detailed results from other trials are required to determine whether RAL consistently results in an increase in cholesterol and if so the consequential long term risks. There were very low levels of emergent PI resistance in either arm of the trial and moderate NtRTI and integrase strand inhibitor resistance in the Control and RAL arms respectively. Further investigations are currently underway exploring the relationships between resistance, virological failure and adherence.

At current prices RAL containing regimens are unlikely to provide a cost effective alternative to standard of care in low income countries. This is likely to be the case until price reductions and generic options become available. However RAL use may provide a viable and durable alternative for second-line therapy in middle and high income countries, and for patients unable to receive NRTI-based regimens.

## Supporting Information

S1 CONSORT checklist (DOC)

S1 Protocol (PDF)

S1 Ethics (DOCX)
