# Correction: Raltegravir Non-Inferior to Nucleoside Based Regimens in SECOND-LINE Therapy with Lopinavir/Ritonavir over 96 Weeks: A Randomised Open Label Study for the Treatment Of HIV-1 Infection

**DOI:** 10.1371/journal.pone.0140623

**Published:** 2015-10-09

**Authors:** Janaki Amin, Mark A. Boyd, Nagalingeswaran Kumarasamy, Cecilia L. Moore, Marcello H. Losso, Chidi A. Nwizu, Lerato Mohapi, Stephen J. Kerr, Annette H. Sohn, Hedy Teppler, Boris Renjifo, Jean-Michel Molina, Sean Emery, David A. Cooper, SECOND-LINE

The group author, SECOND-LINE, was omitted from the author byline and should be listed as the fifteenth author. Please see the full list of contributing authors, as well as their full authorship contributions in the Acknowledgements section of the published article.
