# Supplementary material for: Raltegravir Non-Inferior to Nucleoside Based Regimens in SECOND-LINE Therapy with Lopinavir/Ritonavir over 96 Weeks: A Randomised Open Label Study for the Treatment Of HIV-1 Infection
Source: PLoS One. 2015 Feb 27;10(2):e0118228. doi: 10.1371/journal.pone.0118228 (PMC4344344; doi:10.1371/journal.pone.0118228)
Supplement: S1 Protocol — (PDF) [file pone.0118228.s002.pdf]

NATIONAL CENTRE IN HIV EPIDEMIOLOGY AND CLINICAL RESEARCH

Clinical Protocol:

A randomised open-label study comparing the safety and efficacy of ritonavir boosted lopinavir and 2-3N(t)RTI backbone versus ritonavir boosted lopinavir and raltegravir in participants virologically failing first-line NNRTI/2N(t)RTI therapy: the SECOND-LINE study

Original protocol date: 14th May 2008
Protocol version: Version 2.0
Amendment date: 5th November 2009

Name and address of sponsor:

National Centre in HIV Epidemiology and Clinical Research (NCHECR)
University of New South Wales (UNSW)
Sydney, Australia
Telephone: +61 2 9385 0900
Facsimile: +61 2 9385 0910
Email:SECONDLINE@nchecr.unsw.edu.au

Name and address of Protocol Steering Committee (PSC):

Prof David A Cooper
Principal Investigator
National Centre in HIV Epidemiology and Clinical Research (NCHECR)
University of New South Wales (UNSW)
Sydney, AUSTRALIA
Telephone: +61 2 9385 0900
Facsimile: +61 2 9385 0920
Email: dcooper@nchecr.unsw.edu.au

Prof Brian Gazzard
Principal Investigator
Chelsea & Westminster Hospital
St Stephen's AIDS Trust, 1st Floor, St Stephen's Centre
369 Fulham Road
Fulham, London, SW10 9NH
UNITED KINGDOM
Telephone: + 44 208 746 8239
Facsimile: + 44 208 746 5611
Email: Yvonne.Stupple@chelwest.nhs.uk

Dr Mark Boyd
Principal Clinical Advisor and Project Leader
National Centre in HIV Epidemiology and Clinical Research (NCHECR)
Level 2, CC4 East Wing, UNSW Cliffbrook Campus
45 Beach Street | Coogee NSW 2034 | Australia
Telephone: +61 2 9385 0900
Facsimile: +61 2 9385 0910
Email: mboyd@nchecr.unsw.edu.au

Prof Sean Emery
National Centre in HIV Epidemiology and Clinical Research (NCHECR)
Level 2, CC4 East Wing, UNSW Cliffbrook Campus
45 Beach Street | Coogee NSW 2034 | Australia
Phone: (+ 61 2) 9385 0900
Facsimile: +61 2 9385 0910
Email: semery@nchecr.unsw.edu.au

Dr Hedy Teppler
Merck Research Laboratories
Merck Clinical Research - Infectious Diseases
Mail stop: UG3D-56 USA
Telephone: +1 267-305-7403
Facsimile: +1 267-305-6545
Email: hedy_teppler@merck.com

Chris Woodward
Medical Affairs, Virology
Global Pharmaceutical Research and Development Abbott
21 Pacific Ave, Sinking Spring
Pennsylvania PA 19608 USA
Telephone: +1 847 951 3249
Facsimile: +1 610 670 8569
Email: chris.woodward@abbott.com

*In addition there will be one investigator from each participating country nominated as a member on the PSC (indicated below with an *).*

Name and addresses of the SECOND-LINE study team

Alli Humphries
Senior Clinical Project Coordinator
National Centre in HIV Epidemiology and Clinical Research (NCHECR)
Level 2, CC4 East Wing, UNSW Cliffbrook Campus
45 Beach Street | Coogee NSW 2034 | Australia
Phone: (+ 61 2) 9385 0900
Facsimile: +61 2 9385 0910
Email: ahumphries@nchecr.unsw.edu.au

Jialun Zhou
Project Statistician
St Vincent’s Centre for Applied Medical Research
Level 4, Lowy Packer Building
405 Liverpool Street
Darlinghurst NSW 2010 Australia
Phone: (+ 61 2) 9385 0900
Facsimile: +61 2 9385 0920
Email: jzhou@nchecr.unsw.edu.au
Maria Arriaga
Clinical Project Coordinator

National Centre in HIV Epidemiology and Clinical Research (NCHECR)
Level 2, CC4 East Wing, UNSW Cliffbrook Campus
45 Beach Street | Coogee NSW 2034 | Australia
Phone: (+ 61 2) 9385 0900
Facsimile: +61 2 9385 0910
Email: marriaga@nchecr.unsw.edu.au

Nisha Seneviratne
Clinical Project Coordinator
National Centre in HIV Epidemiology and Clinical Research (NCHECR)
Level 2, CC4 East Wing, UNSW Cliffbrook Campus
45 Beach Street | Coogee NSW 2034 | Australia
Phone: (+ 61 2) 9385 0900
Facsimile: +61 2 9385 0910
Email: nseneviratne@nchecr.unsw.edu.au

Natalie Espinosa
Clinical Project Coordinator
National Centre in HIV Epidemiology and Clinical Research (NCHECR)
Level 2, CC4 East Wing, UNSW Cliffbrook Campus
45 Beach Street | Coogee NSW 2034 | Australia
Phone: (+ 61 2) 9385 0900
Facsimile: +61 2 9385 0910
Email:

Wendy Lee
Project Data Manager
National Centre in HIV Epidemiology and Clinical Research (NCHECR)
Level 1, CC4 East Wing, UNSW Cliffbrook Campus
45 Beach Street | Coogee NSW 2034 | Australia
Phone: (+ 61 2) 9385 0900
Facsimile: +61 2 9385 0910
Email: wlee@nchecr.unsw.edu.au

Rossitza Chevkenova
Project Database Programmer
St Vincent's Centre for Applied Medical Research
Level 4, Lowy Packer Building
405 Liverpool Street
Darlinghurst NSW 2010 Australia
Phone: (+ 61 2) 9385 0900
Facsimile: +61 2 9385 0920
Email: rchevkenova@nchecr.unsw.edu.au

Rosemary Robson
Project Administrative Assistant
National Centre in HIV Epidemiology and Clinical Research (NCHECR)
Level 2, CC4 East Wing, UNSW Cliffbrook Campus
45 Beach Street | Coogee NSW 2034 | Australia
Phone: (+ 61 2) 9385 0900
Facsimile: +61 2 9385 0910
Email: rrobson@nchecr.unsw.edu.au

Investigational Sites and Principal Investigators

AUSTRALIA

NEW SOUTH WALES

ALBION STREET CENTRE
Don Smith
150-154 Albion St
Surry Hills, NSW, 2010
Telephone: +61 2 9332 9611
Facsimile: + 61 2 9332 6519
Email: don.smith@sesiahs.health.nsw.gov.au

ST VINCENT'S HOSPITAL
David Cooper
Immunology B Clinic
Level 4, Xavier Building
Darlinghurst, NSW, 2010
Telephone: + 61 2 8382 4903
Facsimile: + 61 2 8382 4964
Email: dcooper@nchecr.unsw.edu.au

LIVERPOOL HOSPITAL
John Quin
Liverpool Health Service
Immunology HIV Department
Ground Floor, Bigge Park Centre
13 Elizabeth Street
Cnr Bigge & Elizabeth Streets
Liverpool, NSW, 2170
Telephone: + 61 2 9827 8022
Facsimile: + 61 2 9602 4352
Email: john.quin@swsahs.nsw.gov.au

VICTORIA

THE ALFRED HOSPITAL
*Julian Elliott
Infectious Diseases Unit
Level 2, Burnet Institute
85 Commercial Road
Melbourne, Victoria, 3004
Telephone: + 61 3 9076 6077
Facsimile: + 61 3 9076 2431
Email: Julian.elliott@alfred.org.au

CENTRE CLINIC
Ban Kiem Tee
Rear 77 Fitzroy Street
St Kilda, Victoria, 3182
Telephone: + 61 3 9525 5866
Facsimile: + 61 3 9525 3673
Email: bk_tee@vicaids.asn.au

NEW ZEALAND

AUCKLAND HOSPITAL
*Mark Thomas
Infectious Diseases Unit
Park Road, Grafton
Auckland, New Zealand
Telephone: + 64 9 379 7440
Facsimile: +64 9 307 4940
Email: mg.thomas@auckland.ac.nz

ARGENTINA

CAICI
Sergio Lupo
Rodriguez 1215
2000 Rosario Provincia de Santa Fe
ARGENTINA
Telephone: + 54 (341) 424 8045
Facsimile: + 54 (341) 449 6326
Email: sergiolupo@arnet.com.ar

Hospital Central
Victor Bittar
Immunology Service
Alem and Salta - Ground Floor West Wing
5500 Mendoza
ARGENTINA
Telephone: + 54 (261) 420 1920
Facsimile: + 54 (261) 420 2624
Email: victorbittar@ciudad.com.ar

Hospital Italiano
*Waldo Belloso
Gascon 450
C1181ACH, CABA
ARGENTINA
Telephone: + 54 (11) 4959 0393
Facsimile: + 54 (11) 4959 0393
Email: waldo.belloso@hospitalitaliano.org.ar

Eurosistemas S.A.
Arnaldo Casiro
Gorriti 4001
1406 Buenos Aires
ARGENTINA
Telephone: + 54 (11) 4611 6666
Facsimile: + 54 (11) 4612 2805
Email: casiroa@fibertel.com.ar

Hospital J.M. Ramos Mejía
*Marcelo Losso
Servicio de Immunocomprometidos
Urquiza 609
Pabellon de Clinica, 2 Piso
C1221 ADC Buenos Aires
ARGENTINA
Telephone: + 54 (11) 4931 5252
Facsimile: + 54 (11) 4956 1522
Email: mlosso@hivramos.org.ar

Hospital Prof. Alejandro Posadas
Hector Laplume
Av. Presidente Illia y Av. Marconi
El Palomar, Haedo
1706 Provincia de Buenos Aires
ARGENTINA
Telephone: + 54 (11) 4469 9300
Facsimile: + 54 (11) 4469 9279
Email: hlaplume@speedy.com.ar

Hospital Rawson
Norma del Carmen Luna
Bajada Pucará 2025
5000 Córdoba
ARGENTINA
Telephone: +54 (351) 4348754
Facsimile: +54 (351) 422 7452
Email: danielo.david@gmail.com

FUNCEI
Gustavo Lopardo
French 3085,
C1425AWK, CABA
ARGENTINA
Telephone: +54 (11) 5236 7772
Facsimile: +54 (11) 5236 7706
Email: glopardo@intramed.net.ar

Centro de Estudio y Tratamiento Infectológico (CETI)
Jorge Contarelli
Calle 53 #1082 (between 16 and 17)
La Plata, Provincia de Buenos Aires
ARGENTINA
Telephone: +54 (221) 4518082
Facsimile: +54 (221) 4539462
Email:infectologiaceti@gmail.com

Centros de Estudios y Atencion De Infectologia (CEADI)
Oscar Garcia Messina
Guayaquil 866,
C1424CAV - CABA
ARGENTINA
Telephone: +54 (11) 32430151
Email:garciamessina@gmail.com

CHILE

Hospital San Borja-Arriaran
*Marcelo Wolff
University of Chile
Santa Elvira 629
Santiago
CHILE
Telephone: + 56 (2) 551 6738
Facsimile: + 56 (2) 556 5025
Email: marcewolff@yahoo.com

Hospital de la Universidad Católica Pontificia
Carlos Perez
Direccion Medica P8
Marcoleta 367
Santiago
CHILE
Telephone: +56 (2) 354 3103
Email: caperezco@gmail.com

PERU

Instituto de Medicina Tropical Alexander von Humboldt, Universidad Peruana Cayetano Heredia
*Eduardo Gotuzzo
Av Honorio Delgado 430
Urbanizacion Ingenieria
San Martin de Porres
PERU
Telephone: +51 (1) 482 3910
Email: eduardogotuzzo@yahoo.es

IMPACTA
Jorge Sanchez
Av Grau 1010
Barranco
Lima
PERU
Telephone: +51 (1) 242 3072
Email: jsanchez@inmensa.org

Hospital Almenara
Raul Salazar
Av Grau 800 Piso 1B Oeste
Urbanizacion Ingenieria
La Victoria
Lima
PERU
Telephone: +51 (1) 473 0956/475 6378
Email: salazarraul@speedy.com.pe

Via Libre
Robinson Cabello
Jr Paraguay 478
Carcado de Lima
Lima
PERU
Telephone: +51 (1) 203 9900
Email: robinsoncabello@yahoo.com

MEXICO

Instituto Nacional de Ciencias Medicas y Nutricion "Salvador Zubirán"
*Juan Sierra-Madero
Department of Infectious Diseases
Vasco de Quiroga 15, Tlalpan
Mexico D.F.
MEXICO
Telephone: + 52 (5) 655 9675
Facsimile: + 52 (5) 513 8106
Email: jsmadero2@terra.com.mx

Hospital General de Guadalajara
Jaime Andrade Villanueva
Calle Hospital 278 SH
Colonia Alcalde Barranquitas
Guadalajara 44280
Estado de Jalisco
MEXICO
Telephone: +33 (3658) 2848
Facsimile:
Email: jandradev@msn.com

ENGLAND

Chelsea & Westminster Hospital
*Brian Gazzard
369 Fulham Road
Fulham, London
SW10 9NH
UNITED KINGDOM
Telephone: + 44 208 746 8239
Facsimile: + 44 208 746 5611
Email: Yvonne.Stupple@chelwest.nhs.uk

IRELAND

Mater Misericordiae University Hospital
*Paddy Mallon
Catherine McAuley Education and Research Centre
Nelson Street
Dublin 7
IRELAND
Telephone: +353 1 716 6311
Facsimile: +353 1 716 6335
Email: Paddy.Mallon@ucd.ie

FRANCE

Hopital Saint-Louis
*Jean-Michel Molina
Department of Clinical Infectious Diseases
1 Avenue Claude
Vellefaux 75010
Paris
FRANCE
Telephone: +33 (1) 4249 9066
Facsimile: +33 (1) 4249 9067
Email: jean-michel.molina@sls.aphp.fr

GERMANY

Johann Wolfgang Goethe-University Hospital
*Christoph Stephan
HIVCENTER Building No. 68
Theodor-Stern-Kai 7
60590 Frankfurt
GERMANY
Telephone: +49 (69) 6301 7680
Facsimile: +49 (69) 6301 5712
Email: Christoph.Stephan@hivcenter.de

Medical Group Practice
Heiko Jessen
Medical Group Practice - General Medicine and HIV
Motzstr 19
Berlin 10777
GERMANY
Telephone: +49 (30) 235 10 70
Facsimile: +49 (30) 235 10 722
Email: heiko.jessen@gmx.de

ISRAEL

Rambam Medical Center
*Eynat Kedem
Immunology Allergy & AIDS Institute
B. Rappaport Faculty of Medicine, Technion
Haifa 31096
ISRAEL
Telephone: +972 4 854 3581
Facsimile: +972 4 854 3327
Email: e_kedem@rambam.health.gov.il

MALAYSIA

University of Malaya Medical Centre
*Adeeba Kamarulzaman
Clinical Investigations Centre (CIC)
13th Floor, Menara Utama
Lembah Pantai
Kuala Lumpur 50603
MALAYSIA
Telephone: + 60 (3) 7950 2168
Facsimile: + 60 (3) 7955 7740
Email: adeeba@ummc.edu.my

Hospital Sungai Buloh
Christopher Lee
Department of Medicine
Jalan Hospital
47000, Sungai Buloh
Selangor
MALAYSIA
Telephone: +60 (3) 6145 4333
Facsimile: +60 (3) 6157 1703
Email: chrislee@hkl.gov.my

Hospital Pulau Pinang
Chow Ting Soo
Jalan Residens
10990 Georgetown
Pulau Pinang
MALAYSIA
Email: tingsoochow@yahoo.com

HONG KONG

Queen Elizabeth Hospital
*Patrick Li
30 Gascoigne Road
Kowloon
HONG KONG
Telephone: + 852-295-86731
Facsimile: + 852-238-44698
Email: lickp@ha.org.hk

TAIWAN

National Taiwan University Hospital
*Chien-Ching Hung
Section of Infectious Diseases Department of Internal Medicine
No. 7 Chung-Shan South Road
Taipei, TAIWAN
Telephone: +886 02 397 0800 Ext 2743 or 3726
Facsimile: + 886 02 397 1412
Email: hcc0401@ntu.edu.tw

SINGAPORE

Tan Tock Seng Hospital
*Poh-Lian Lim
Infectious Diseases Research Centre
11 Jalan Tan Tock Seng
Singapore 308433
SiNGAPORE
Telephone: + 65 6357 7924
Facsimile: +65 (6252) 3540
Email: poh_lian_lim@ttsh.com.sg

THAILAND

HIV-NAT Program on AIDS - Thai Red Cross AIDS Research Centre
*Praphan Phanuphak
HIV-NAT Research Collaboration
104 Ratchadamri Road
Pathumwan
Bangkok 10330
THAILAND
Telephone: +66 2 252 7888
Facsimile: +66 2 252 5779
Email: praphan.p@chula.ac.th

Khon Kaen University
Ploenchan Chetchotisakd
Division of Infectious Disease and Tropical Medicine
Department of Medicine
The Srinagarind Hospital, Khon Kaen University
123 Mitraparb Highway
Khon Kaen 4002
THAILAND
Telephone: +66 43 363 654
Facsimile: +66 43 203 772
Email: ploencha@kku.ac.th

Research Institute for Health Sciences (RIHES), Chiang Mai University
Khuanchai Supparatpinyo
110 Intavaroros Road
Sriphum, Muang
Chiang Mai 50200
THAILAND
Telephone: +66 5394 5055-8
Facsimile: +66 5322 1849
Email: khuanchai@idthai.org

Chonburi Hospital,
Chureeratana Bowonwatanuwong
Chonburi 20000
THAILAND
Telephone: +66 38-931395
Email: c.bowon@gmail.com

Chiang Rai Prachanukroh Hospital
Pacharee Kantipong
1039 Sathanpayaban Road
Muang District
Chiang Rai 57000
THAILAND
Telephone: +66 53 711300 ext 1784
Email: pachareek@hotmail.com

Khon Kaen Hospital
Niramon Leerattanapecth
56 Srichan road, Amphor Muang
Khon Kaen 40000
THAILAND
Phone: + 66 81 876-8566
Email : niram02@yahoo.com

CHINA

DiTan Hospital
Hongxin Zhao
No8 Jingshun Dongjie
Chaoyang District
Beijing
China
Telephone: +86 (10) 8432 2581
Email: zhao_hongxin66@yahoo.com.cn

PUMCH
Taisheng Li
1 Shuaifuyuan Wangfujing
Beijing
China, 100730
Telephone: +(8610) 6529 5086
Facsimile: + (8610) 6529 5046
Email: litsh@263.net

Beijing You An Hospital
Hao Wu
No. 8 Xi Tou Tiao
You'an Men Wai
Fent Tai District
Beijing
China
Tel: +86 (10) 6305 3963
Facsimile: +86 (10) 6329 4417
Email: wuhd@public.bta.net.cn

Guangzhou No 8 People's Hospital
Cai Weiping
627 Dongfeng Dong Road
Guangzhou
P.R. China 510060
Telephone: +(8620) 83710422
Email: caiwp@163.net

Shanghai Public Health Clinical Centre (Fudan University)
Hongzhou Lu
2901 Caolang Road
Jinshan District
Shanghai
China, 201508
Telephone: +(8621) 5724 8758
Facsimile: + (8621) 5724 8758
Email: luhongzhou@fudan.edu.cn

INDIA

YRGCARE Medical Center VHS
*Nagalingeswaran Kumarasamy
Y.R.Gaitonde Medical, Educational & Research Foundation
Voluntary Health Services
Rajiv Gandhi Salai
Taramani
Chennai 600 113
INDIA
Telephone: +91 (44) 39106818
Email: kumarasamy@yrgcare.org

Institute of Infectious Diseases
Sanjay Pujari
Kumar Business court, D wing
1st Floor, Kumar Business Court, D wing
Mukundnagar, Pune 411037
India
Telephone: +91 20 24274950
Email: sanjaypujari@gmail.com

NIGERIA

Plateau State Specialist Hospital
*Chidi Nwizu
Institute Of Human Virology
North East Regional Office
No 11 Niven Close off
Ibrahim Taiwo Road GRA
Jos
Plateau State
Nigeria
Telephone: +2348086435943
Facsimile:
Email: canwiz@pol.net

Jos University Teaching Hospital (JUTH)
Oche Agbaji
2 Murtala Mohammed Way
Jos
Plateau State
NIGERIA
Tel: +234 803 349 1857
Email: oagbaji@yahoo.com

Evangel Hospital (ECWA)
Chidi Nwizu
P.M.B. 2238
Jos
Plateau State
NIGERIA
Email: canwiz@pol.net

SOUTH AFRICA

JOSHA Research Bloemfontein
Sharne Foulkes
1st Floor, Rubins Building
28 East Burger Street
Bloemfontein
SOUTH AFRICA
Telephone: + 27 514 128 160
Facsimile: + 27 514 128 190
Email: sharne.foulkes@josha.co.za

Desmond Tutu HIV Foundation
Robin Wood
Wernher Beit Building North
UCT Medical School
Anzio Road
Observatory 7925
Cape Town, South Africa
Phone: +27 21 650 6726
Fax: +27 21 650 6963
Email: Robin.Wood@hiv-research.org.za

Chris Hani Baragwanath Hospital
*Lerato Mohapi
University of Witwatersrand
Soweto
South Africa
Email: mohapil@hivsa.com

SECOND-LINE PROTOCOL AMENDMENT LIST

Original protocol date: 14th May 2008

Previous protocol version: Version 1.0 (2nd July 2009)

Current protocol version: Version 2.0

Amendment date: 28th October 2009

Please find below the list of amendments that have been made to the SECOND-LINE protocol Version 1.0 (2nd July 2009) to create the current version Version 2.0 (28th October 2009)

Chapter/Section	Page #	Issues/Comments
Cover page	1	Update date and protocol number.
PSC Committee	2	Add details for Hedy Teppler from Merck.
PSC Committee	2	Add details for Chris Woodward from Abbott.
PSC Committee	3-10	Added in an * for those site PIs on the PSC committee.
Site list	3-10	Removed Vietnam from site list and updated some contact details.
Protocol synopsis	19	Add details of dose, mode of administration, and duration of treatment of ritonavir boosted lopinavir soft gelatin capsules: 133.3mg/33.3mg 6 capsules once daily or 3 capsules twice daily.
Protocol synopsis	19	Add details of ritonavir boosted lopinavir availability both tablet and soft gelatin capsule formulations.
Protocol synopsis	20	Added a sentence to clarify that the third year of drug supply will only be to those sites/countries where the drugs are not available via the local health care system
Schedule of assessments	23	Superscripts h and i were inserted incorrectly in the table. These were switched back around to be sequential.
Schedule of assessments	23	Made the X grey for plasma HIV drug resistance test at screening as this is only required if the site is a "genotypic site". Plus, added an X at week 0 for the central drug resistance test. This has also been updated under subscript k
Schedule of assessments	23	Removed the "x" from dispense study medication at the week 96 visit.
Schedule of assessments	23	Under subscript e we have changed the wording to indicate that Hep B should be done at screening if the previous tests have been negative or there is no report to verify a previous positive test.
4.1	30	Changed the number of sites from 30 to approximately 50, and added New Zealand to the country list.
5.1	32	Add details of dose, mode of administration, and duration of treatment of ritonavir boosted lopinavir soft gelatin capsules: 133.3mg/33.3mg 6 capsules once daily or 3 capsules twice daily.
5.2.2	32	Added in additional language about the Kaletra soft gel capsules that will be available for use by sites.
5.2.2	33	Added in language about Kaletra not being sent to Australian sites. In Australia the costs associated with provision of Kaletra will be reimbursed via NCHECR.
5.2.3	33	Updated the information on the ALMAC depots that will be used for a central repository and shipment.
5.4	34	Additional language added to the selection on NRTIs regarding mandatory selection of innovator or FDA/WHO approved generic formulations.
5.13	41	Add more detail about prohibited drugs.
6.1.2	43	Clarified that Hep C antibody need only be done if previous test results have been negative or if there is no report available to confirm a positive result.
8.2 & 8.5	48 & 49	Updated the information on how to report a pregnancy on study. It needs to be an expedited report and therefore will be completed on the SAE form
8.5	50	Highlighted in bold the 24hour reporting time requirement for SAE reporting. Also stated that 24 hours equals one working day.
9.2	51	Updated the number of labels that are on the booklet label from 8 to 13. Also indicated that some labels are single-panel English labels.

9.2 AND 9.3	52	Drug packaging, labelling and dispensing sections have been updated to include Kaletra soft-gel capsules.
10.1	53	Remove information about SydPath blood packs being provided for collection on spare serum and plasma samples. Blood tubes will be sourced locally and reimbursed via the study budget.
10.7	54	Removed the statement that the first interim analysis will be conducted on locally measured HIV-RNA.
APPENDIX 2	81	Update the template for the patient information and consent form with the changed blood volumes collected at each visit.
APPENDIX 2	81	Updated the side effects profile of raltegravir
APPENDIX 2	82	Updated the table of adverse events to include those for Kaletra and Aluvia in the template for the patient information and consent form.
APPENDIX 2	83	Updated the patient information and consent form in regards to compensation in case of injury to make the template generic for ALL sites, not just Australia

Investigator agreement and signature page

between the NCHECR and the study investigator (s)

Principal Investigator: ____

Co-investigators (please list, if applicable): _________________________

This study will compare two SECOND-LINE regimens of combination antiretroviral therapy in people living with HIV infection (PLHIV) experiencing confirmed virological failure of a first-line NNRTI/2N(t)RTI regimen. The trial will enrol participants across a range of high-middle income sites in Australia, Asia, Latin America, Europe and low income sites in sub-Saharan Africa. Participants will be randomised to receive either boosted lopinavir + 2-3N(t)RTIs or boosted lopinavir + raltegravir. The study will be conducted for 96 weeks and is expected to involve 550 eligible participants.

Clinical work is expected to start in the second half of 2009. This trial protocol, ICH Guidelines on Good Clinical Practice, the NCHECR standard operating procedures, the Therapeutic Goods Administration Access to Unapproved Therapeutic Goods – Clinical Trials in Australia regulations and the Declaration of Helsinki will be adhered to in all cases.

All patient data will be completely and accurately recorded in the patient case record forms. Case record forms will be completed by the site following study visits. Patient medical records will also be kept up-to-date regarding study information. The trial monitor will be allowed to review the patient case record forms, the source medical records, the trial progress and the consent forms. In addition, the sponsor's auditors and regulatory authority auditors will be allowed access to all study documents, including source documents, if necessary.

Before entering this study, all participants, or in special cases, their legal representative or guardian will give their written informed consent. The signed consent forms will be retained by the investigator(s)/co-investigator(s) listed below.

I/We accept responsibility for the conduct of the research detailed in the proposal including all protocol-specific assessments, and I/we agree to abide by all decisions made by our Ethics Committee.

I/We agree to the above, which, in conjunction with the NHMRC Statement on Ethical Conduct in Research Involving Humans, will serve as the basis for co-operation in this study.

RESPONSIBLE INVESTIGATOR (signature and date)

____

CO-INVESTIGATORS (signature and date)

____

SPONSOR'S REPRESENTATIVE
(signature and date)

____

NCHECR MONITOR(S)/COORDINATOR (S)

____

TABLE OF CONTENTS

Name and address of sponsor: 1

Name and address of Protocol Steering Committee (PSC) : 1

Investigational Sites and Principal Investigators 4

Investigator agreement and signature page 12

PROTOCOL SYNOPSIS........ 19

Flow chart/time and events schedule 18

1 INTRODUCTION........ 25

1.1 Background and rationale 25

1.2 Rationale for investigation of a dual therapy, N(t)RTI sparing regimen 27

1.3 Research hypothesis 28

2 STUDY OBJECTIVES........ 29

2.1 Primary objective 29

2.2 Secondary objectives........ 29

2.3 Exploratory objectives........ 29

3 STUDY POPULATION 29

3.1 Eligibility criteria 29

3.2 Inclusion criteria........ 29

3.3 Exclusion criteria 29

3.4 Withdrawal of subjects from study 30

4 TRIAL DESIGN........ 30

4.1 Study design........ 30

4.2 Criteria for evaluation 31

5 TREATMENT OF SUBJECTS........ 33

5.1 Treatment group assignment 33

5.2 Study drugs 33

5.3 Genotypic resistance testing 34

5.4 Backbone N(t)RTI selection 34

5.5 Clinical management of ART regimen failure 36

5.6 Management of ART intolerance 37

5.7 Dose modifications 37

5.8 Toxicity management 37

5.9 Protocol-specific toxicity management guidelines 39

5.10 Management of AIDS Defining Illnesses (ADI) and Immune Restoration Disease (IRD) 41

5.11 Discontinuation of therapy 41

5.12 Treatment adherence 42

5.13 Prohibited and restricted therapies during the study 42

5.14 Concomitant medication 35

6 STUDY PROCEDURES 43

6.1 Initial screening period 43

6.2 Randomisation visit 45

6.3 Follow-up visits 46

7 DATA SAFETY MONITORING BOARD (DSMB) 47

8 ADVERSE EVENT REPORTING 48

8.1 Definition and reporting of an overdose 49

8.2 Reporting of pregnancy 49

8.3 Adverse events 49

8.4 Serious non-AIDS events (SNAEs) 49

8.5 Serious adverse events (SAEs) 50

8.6 Laboratory test abnormalities 51

8.7 Other safety considerations 52

9 PACKAGING, LABELLING and ACCOUNTABILITY of TRIAL SUPPLIES 52

9.1 Study drugs 52

9.2 Packaging and labeling 52

9.3 Handling and dispensing of study drugs 53

9.4 Study drug records at investigational site(s) 53

10 BIOLOGICAL SAMPLES 54

10.1 Blood collection 54
10.2 Labeling of specimen tubes 54
10.3 Transportation of samples to pathology 54
10.4 Storage of samples 55
10.5 Processing of samples 55
10.6 Reporting of results 55
10.7 Plasma HIV RNA determination 55
11 STATISTICAL METHODOLOGY 55
11.1 Sample size determination and statistical analysis 55
11.2 Supplementary sample size assessments for exploratory analysis 47
11.3 Analysis plan 48
11.4 Schedule of analyses 49
11.5 Demographics and baseline characteristics 51
11.6 Efficacy analyses 51
11.7 Subgroup analyses 51
11.8 Safety analyses 52
12 DATA COLLECTION, SOURCE DOCUMENTS AND RECORD RETENTION 60
12.1 Data collection and source documents 60
12.2 Records retention 61
12.3 Monitoring for protocol compliance 61
12.4 Auditing 62
13 ETHICS COMMITTEE/REGULATORY APPROVAL AND INFORMED CONSENT 62
13.1 Ethics 62
13.2 Institutional review board/independent ethics committee (IRB/IEC) 62
13.3 Compliance with the protocol and protocol revisions 63
13.4 Informed consent 63
13.5 Informed consent procedures 63
14 CONFIDENTIALITY OF DATA 65
14.1 Confidentiality of participant records 65

14.2 Confidentiality of study data 65

15 FINANCING AND INSURANCE 65

16 QUALITY CONTROL AND QUALITY ASSURANCE 66

17 PUBLICATION POLICY 66

References 67

Appendix 1: Definitions and Criteria For HIV Disease and AIDS Events 71

Appendix 2: Template Patient Information Statement and Consent Form 71

Appendix 3: Diagnostic Criteria for Serious Non-AIDS Events 80

PROTOCOL SYNOPSIS

Title of Study:

A randomised open-label study comparing the safety and efficacy of a ritonavir-boosted lopinavir and 2-3N(t)RTI backbone versus ritonavir-boosted lopinavir and raltegravir in participants virologically failing first-line NNRTI/2N(t)RTI therapy: the SECOND-LINE study.

Estimated Number of Study Centres and Countries/Regions:

Approximately 50 selected centres in Australia, Africa, Asia, Latin America, and Europe.

Primary Objective

The primary objective of this study is to compare the virological efficacy of the two strategies as measured by the proportion of participants with HIV RNA <200 copies/mL 48 weeks after randomisation.

Research Hypothesis

In HIV-infected subjects who have virologically failed first-line antiretroviral therapy comprising 2N(t)RTI + NNRTI a regimen of second-line therapy incorporating ritonavir-boosted lopinavir and raltegravir provides comparable (i.e. non-inferior) antiretroviral efficacy over 48 weeks to a regimen containing ritonavir-boosted lopinavir and 2-3N(t)RTIs.

Study Design:

This is a Phase IIIb/IV, international, randomised, open label study comparing two regimens of combination antiretroviral therapy in people living with HIV with confirmed virological failure of first-line NNRTI/2N(t)RTI regimens. The study will run for 96-weeks but the primary analysis will take place at the week 48 time point.

Eligible participants will be randomised in equal proportions to one of two regimens of combination ART as follows:

I. ritonavir boosted lopinavir (LPV/r) + 2-3N(t)RTIs
II. ritonavir boosted lopinavir (LPV/r) + raltegravir

Randomisation will be stratified for the following variables:

1. Clinical site
2. Plasma HIV-1 RNA viral load < or ≥ 100,000 copies/mL

Number of Subjects per Group:

Approximately 275 eligible subjects will be randomly allocated to each of the two treatment arms giving a study total of 550 participants.

Study Population:

Eligible participants will satisfy all of the following criteria within 45 days prior to randomisation.

Inclusion criteria:

1. HIV-1 positive by licensed diagnostic test
2. Aged 16 years or older (or minimum age as determined by local regulations or as legal requirements dictate)
3. Have received first antiretroviral regimen consisting of an NNRTI plus 2N(t)RTIs for ≥ 24 weeks
4. No change in antiretroviral therapy within 12 weeks prior to screening
5. Failed first-line NNRTI + 2N(t)RTI combination therapy according to virological criteria defined by two consecutive (≥7 days apart) HIV RNA results of >500 copies/mL
6. No prior or current exposure to HIV protease inhibitors and/or HIV integrase inhibitors
7. Able to provide written informed consent

Exclusion criteria:

1. The following laboratory variables:
   a) absolute neutrophil count (ANC) <500 cells/μL
   b) hemoglobin <7.0 g/dL
   c) platelet count <50,000 cells/μL
   d) ALT >5 x ULN
2. Pregnant or nursing mothers
3. Participants with active viral hepatitis B infection defined by the presence in serum of hepatitis B surface antigen
4. Use of immunomodulators within 30 days prior to screening
5. Use of any prohibited medications (rifampicin, midazolam, triazolam, cisapride, pimozide, amiodarone, dihydroergotamine, ergotamine, ergonovine, methylergonovine, astemizole, terfenadine, vardenafil, and St. John's wort)
6. Intercurrent illness requiring hospitalisation
7. Active opportunistic disease not under adequate control in the opinion of the site Principal Investigator
8. Participants with current alcohol or illicit substance abuse that in the opinion of the site Principal Investigator might adversely affect participation in the study
9. Participants deemed by the site Principal Investigator unlikely to be able to remain in follow-up for the protocol-defined period

Study Drugs, Dose and Mode of Administration, Duration of Treatment:

I. ritonavir boosted lopinavir (LPV/r) 200mg/50mg 4 tablets once daily or 2 tablets twice daily (OR 133.3mg/33.3mg 6 soft gelatin capsules once daily or 3 soft gelatin capsules twice daily) + 2-3N(t)RTIs

II. ritonavir boosted lopinavir (LPV/r) 200mg/50mg 4 tabs once daily or 2 tabs twice daily (OR 133.3mg/33.3mg 6 soft gelatin capsules once daily or 3 soft gelatin capsules twice daily) +

raltegravir 400mg twice daily

A maximum of 3 years drug supply (2 years of trial and one year post study) will be provided to study participants. If the study drugs are available via the local healthcare system at the end of the two years, the 3rd year supply will not be provided. Raltegravir will be in tablet form and should be taken orally. Ritonavir boosted lopinavir will be available in both soft gelatin and tablet formulations. Selection of the N(t)RTI component of study arm I will be at the discretion of the treating physician according to local drug availability. Recommendations about the composition of the N(t)RTI backbone are made in section 5.4 of this protocol. For each combination ART regimen, section 5.5 of this protocol recommends options for episodes of drug intolerance that preserve the SECOND-LINE composition of the regimen where possible.

Primary endpoint (week 48 and 96)

The proportion of participants with HIV RNA <200 copies/mL.

Secondary endpoints (week 48 and 96)

A number of secondary endpoints will be examined in this protocol by randomised treatment arm. These will include, but not be limited to the following:

Virological

- an analysis of the time to loss of virological response (TLOVR)
- proportions of participants with <50 and <400 HIV RNA copies /mL
- time to plasma HIV RNA <200 copies/mL

Immunological

- mean change in CD4+ cell count from baseline

Safety

- total number of participants with any serious adverse events (SAEs), and the cumulative incidence of SAEs
- total number of participants with any adverse event(s) (AEs) and the cumulative incidence of all AEs
- total number of participants with any adverse event (AE), and cumulative incidence of all AEs associated with cessation of randomly assigned therapy

Antiretroviral treatment

- analyses in which participants who change ART for any reason are classified as having failed randomised treatment
- time to change in randomly assigned therapy (all reasons individually and on aggregate)

Exploratory Endpoints (week 48 and 96)

A number of exploratory endpoints will be examined in this protocol by randomised treatment arm. These will include, but not be limited to the following:

Clinical

- total number of AIDS events and deaths
- total number of serious non-AIDS events

Metabolic and anthropometric

- mean/median changes from baseline in fasted lipids (TC, LDL-c, HDL-c, and TG), insulin and blood glucose
- changes in body morphology as determined by waist and hip measurements

Resistance

- patterns of genotypic HIV resistance associated with virological failure

Adherence

- adherence and virological outcomes (using the CPCRA instrument)

Quality of Life

- quality of life assessment using the SF-12 instrument

Statistical Methods:

The primary endpoint is the comparison of proportions of participants in each arm whose plasma HIV RNA is <200 copies/mL after 48 weeks. Under the assumption that there is no difference between randomised treatment policies, to have 90% power to demonstrate non-inferiority in the intention to treat (ITT) analysis using a 12% margin will require 248 participants per arm to be randomised (2-sided alpha=5%) making a total of 496 participants.

To ensure that the per protocol (PP) analysis also has 90% power to demonstrate non-inferiority, the sample size needs to be increased for participants who switch ART due to treatment toxicity over the course of the first 48 weeks of study. In this population this is estimated to be no more than 10% of randomised participants. Therefore, to ensure 90% power to demonstrate non-inferiority on the ITT and subsequently on the PP analyses, a total of 550 (i.e. 273 per arm, round to 550) participants will be randomised. The power of the study for the ITT analysis in this case is 92.9%.

Schedule of Analyses:

There will be four formal analyses conducted within this protocol: two interim analyses of week 24 data (one when 125 participants in each arm reach week 24; a second when all participants reach week 24), a primary analysis at week 48 (all participants complete week 48) and a follow up confirmatory analysis at week 96 (all participants complete week 96).

Both interim analyses will be reviewed by an independent Data Safety Monitoring Board (DSMB) which will recommend to the Protocol Steering Committee (PSC) that the study should continue unchanged or be amended in light of observed differences between treatment arms or aspects of study conduct that warrant modification (e.g. poor recruitment, safety concerns, and substantial losses to follow-up). Their review of treatment regimens will be coded as A and B. The DSMB, in agreement with the PSC, may require stopping rules to be implemented that require changes in the conduct of the study should concerns about the safety

of participants arise from review of the week 24 results. A conservative Peto-Prentice type stopping rule ($p<0.001$) will be used to judge whether one arm should be ceased for inferiority at each of the two interim analyses.

Flow chart/time and events schedule

	Study week number[a]										
**Procedure**	**SCR[b]**	**0**	**4**	**12**	**24**	**36**	**48**	**60**	**72**	**84**	**96**
**Clinical Assessments**											
Informed Consent	X										
Medical History	X										
Vital Signs[c]	X	X	X	X	X	X	X	X	X	X	X
Adverse Events Assessment		X	X	X	X	X	X	X	X	X	X
Anthropometric studies[d]		X					X				X
Quality of life		X					X				X
Adherence			X				X				X
**Laboratory Assessments**											
Hepatitis B surface antigen	X										
Hepatitis C antibody[e]	X										
Pregnancy Test[f]	X	X	X	X	X	X	X	X	X	X	X
Biochemistry[g]	X	X	X	X	X	X	X	X	X	X	X
Haematology[h]	X	X	X	X	X	X	X	X	X	X	X
Plasma HIV-1 RNA[i]	X	X	X	X	X	X	X	X	X	X	X
CD4+ and CD8+ T cell (absolute and %)	X	X	X	X	X	X	X	X	X	X	X
Fasted metabolic parameters[j]		X	X	X	X		X		X		X
Plasma HIV drug resistance test[k]	X	X									
Plasma and serum preparation for storage		X	X	X	X	X	X	X	X	X	X
Plasma storage for central HIV-RNA		X	X	X	X	X	X	X	X	X	X
PBMC preparation and storage		X									
**Clinical Drug Supplies**											
Dispense Study Medication		X	X	X	X	X	X	X	X	X	

a All efforts should be made to schedule visits in keeping with the proposed week numbers. Visit windows are: week 4 (from week 2 to week 6), week 12-96 (actual visit can be 6 weeks either side of the scheduled visit date)
b All screening assessments must be completed within 45 days prior to the date of randomisation
c Vital signs include blood pressure, temperature, pulse, weight, height (baseline only)
d Anthropometric studies include umbilical waist and maximum hip measurement
e Hepatitis C antibody results need only be done at screening if previous test results were negative or there is no report available to confirm a positive result
f If female and of child bearing potential
g Biochemistry includes: electrolytes (sodium, potassium, bicarbonate, chloride, phosphate), creatinine, urea, ALT, creatinine kinase and total bilirubin. (aniona gap, creatinine clearance and glomerular filtration rate will be automatically calculated on the eCRF)
h Haematology includes: haemoglobin, white cells, neutrophils, lymphocytes, platelets
i All HIV-1 RNA assays will be run by a laboratory using an assay with a lower limit of detection of 50 copies/mL in plasma
j Fasting parameters includes: glucose, total cholesterol, HDL cholesterol, calculated LDL cholesterol, triglycerides and insulin (homeostasis model assessment-insulin resistance (HOMA-IR) will be automatically calculated on the eCRF)
k If a site elects to conduct genotypic resistance testing, testing on all participants at that site must be conducted at screening. However, ALL participants will have plasma stored at week 0 for HIV resistance genotype testing which will be centrally analysed

INTRODUCTION

1.1 Background and rationale

The emergence of the new antiretroviral therapy (ART) class of HIV-integrase strand transfer inhibitors (InSTIs) and data suggesting that the first-in-class drug raltegravir offers substantial and rapid anti-HIV potency means that the current paradigms of ART management may be revolutionised in the near future [1-3].

Current treatment guidelines recommend the construction of initial combination regimens derived from three classes – nucleoside/nucleotide reverse transcriptase inhibitors (N(t)RTI), non-nucleoside reverse transcriptase inhibitors (NNRTI) and protease inhibitors (PI) [4,5]. The selection of the component drugs for inclusion in a regimen of combination antiretroviral therapy includes consideration of the following factors: potency; pill burden, dosing frequency, food restrictions, overlapping toxicities, genetic barrier to HIV drug resistance; and overall impact on activities of daily living and quality of life. The availability of potent new agents increases the possible combinations of antiretroviral therapy.

While the evidence base for the use of NNRTI+2N(t)RTI (2N(t)RTI defined as two drugs from the N(t)RTI class) as first-line therapy is reasonably well established, what should be done when first-line therapy fails is not [6]. The majority of the evidence that informs our current decision making regarding the management of antiretroviral regimens is based on studies conducted in antiretroviral naïve participants, or in those who have been exposed to and failed numerous N(t)RTI, NNRTI and PI containing regimens ('deep salvage'). In the case of first-line failure (i.e. defined as 'virological failure' and not simply intolerance of one or more components of the first-line regimen), current guidelines generally recommend the introduction of a ritonavir pharmaco-enhanced or 'boosted' PI (PI/r) and the use of new or recycled N(t)RTIs. Guidelines also allow for the maintenance of lamivudine (or emtricitabine) with a new N(t)RTI in a second line regimen because of evidence that the presence of the typical lamivudine/emtricitabine mutation (M184V) reduces viral fitness, confers residual antiviral activity, and improves viral sensitivity to zidovudine or tenofovir [4,5]. However, as compared to the case of first-line therapy, none of these recommendations are based on evidence gathered from optimal study designs, and the strength of evidence is therefore considered no more than moderate [6].

Guidelines generally recommend the use of an HIV resistance genotype assay if available to guide decision making in selection of second-line therapy. There is however no conclusive proof that HIV resistance genotype testing contributes significantly to virological response after failure of first-line therapy [7,8].

The need for a better, evidence based understanding of the optimal therapy to follow first-line NNRTI+2N(t)RTI failure is made more urgent by the ART roll-out efforts in the developing world. WHO/UNAIDS announced in April 2007 that its 'Three by Five' campaign had registered two million HIV-infected people in developing countries receiving ART [9]. This, in combination with the efforts of The United States President's Emergency

Plan for AIDS Relief (PEPFAR), Médicins Sans Frontières (MSF) and other non-governmental organisations and developing world governments means that many HIV-infected individuals are likely to need second-line therapy in the near future after the inevitable failure of first-line ART [10]. To compound the problem, most of those accessing combination ART in resource-limited settings do not receive regular monitoring of plasma HIV RNA, and therefore, may remain on failing first-line regimens for far longer than those receiving the same drug combinations in settings in which both routine HIV RNA monitoring and alternative ART combinations are readily available [11,12]. It is likely in this latter situation that individuals will accumulate substantial resistance to the N(t)RTI (and NNRTI) class, and therefore the response to second-line combination therapy may be attenuated compared to that seen in developed countries (i.e. those placed on standard PI/r+2-3N(t)RTI second-line regimens may effectively be receiving PI/r monotherapy as a result of multi-N(t)RTI resistance) [13-15]. A computer simulation model suggests that in ART programs in which only clinical criteria are used for monitoring of standard first-line combination ART consisting of nevirapine (NVP) plus stavudine (d4T) and lamivudine (3TC), at least half will have selected at least 3 or more thymidine-analogue mutations at the time of switch [16]. Finally, evidence suggests that resistance to the NNRTIs is associated with a greater risk of death compared to resistance to PIs [17,18].

The availability of raltegravir, coupled with a lack of robust evidence supporting the use of a PI/r+2-3N(t)RTI as an effective second-line strategy following the failure of a first-line combination consisting of an NNRTI+2N(t)RTI provides an opportunity to investigate a new SECOND-LINE approach. The experimental arm of this study will consist of raltegravir coupled with a ritonavir-boosted PI versus a control arm comprising a ritonavir-boosted-PI+2N(t)RTI (the current guideline recommended standard of care) in participants with documented failure of conventional first-line NNRTI+2N(t)RTI combination therapy.

The PI/r to be used in both the experimental and control arms is heat stable ritonavir-boosted lopinavir (LPV/r). Heat stable LPV/r is an attractive option for a number of reasons. Firstly, it is co-formulated product that does not require refrigeration, a distinct advantage in middle- and low-income countries which are located in tropical and sub-tropical zones. Secondly it has acted as the 'gold-standard' boosted-PI since it was proven to be superior to nelfinavir in 2002 [19]. Since that time a substantial evidence base including data from a number of randomised trials in both treatment-naïve and -experienced participants has accumulated which has repeatedly demonstrated its efficacy and safety [20-22]. More recently it has been demonstrated in a randomised, controlled trial that LPV/r administered at 800/200 mg once daily provides a non-inferior virological response compared to that achieved with administration of LPV/r 400/100 mg twice daily, with similar rates of study discontinuation and adverse events [23]. Therefore this study protocol will allow prescription of LPV/r once daily or twice daily depending upon the preference of the investigator in discussion with study participants.

This randomised study will permit the development of a stronger evidence base for second-line therapy while at the same time testing an innovative, attractive and compact regimen against the currently recommended standard(s) of care. The study is SECOND-LINE in nature as it aims to not only test a novel dual ART combination but also a new management strategy. The new management strategy aims to prove that drugs selected from

two drug classes to which a patient is naïve is non-inferior to the currently accepted standard-of-care strategy in which an agent from a new class (PI) is combined with two drugs from a class to which the patient has previously been exposed (N(t)RTIs). Furthermore, the experimental dual combination arm of this study represents an antiretroviral management strategy consistent with the 'public health approach' recommended for use in resource-limited settings [24]. If this approach is proven effective it would open up a simplified ART management pathway consisting of first line ART incorporating two ART drug classes (NNRTI+2N(t)RTI) followed by second-line therapy incorporating two independent ART drug classes, PI (ritonavir-boosted PI) + InSTI (raltegravir))

1.2 Rationale for investigation of a dual therapy, N(t)RTI sparing regimen

The era of combination antiretroviral therapy has been characterised by the use of 'triple-therapy' and a proposal to use only 2 agents from 2 new classes to construct a second-line therapy might be viewed as inadequate. However, there is evidence that the use of two potent antiretroviral drugs in combination is sufficient to confer powerful and durable antiretroviral activity. The Merck 006 study suggested that a combination of unboosted indinavir and efavirenz conferred similar antiretroviral efficacy to a standard combination of unboosted indinavir plus 2N(t)RTIs [25]. This experience was repeated in the ACTG 5142 study in which a combination of boosted-lopinavir and efavirenz was comparable to that of boosted-lopinavir and 2N(t)RTIs [26]. A single arm study in Thailand took 61 participants failing combination N(t)RTIs and enrolled them in a study of boosted indinavir 800/100 mg tid and efavirenz 600 mg qd. Four year follow-up of the study demonstrated that 80% of all enrolled participants were maintaining a viral load <50 copies/ml on the same ART combination [27,28]. One recent study investigated and reported the efficacy of dual therapy with ritonavir-boosted lopinavir and tenofovir with conventional triple therapy consisting of boosted-lopinavir used with various combinations of 2N(t)RTIs. The study, while small, demonstrated very similar outcomes in both arms for virological control at 400 and 50 copies/ml. CD4 count increase from baseline was significantly better in the dual therapy arm [29].

Evidence derived from antiretroviral salvage trials conducted in multi-class experienced participants provides indirect supporting evidence for the concept of the use of 2 potent agents as effective combination ART. Most late salvage trials have demonstrated the best virological responses in those receiving the greatest number of fully active agents [30-34]. For instance, the addition of enfuvirtide in those naïve to the drug, improved virological responses in both the *RESIST* and *POWER* studies. In these studies, participants who received the active protease inhibitor and enfuvirtide as a novel agent received at most two fully active agents. More recent salvage studies have been able to examine the benefit of three fully active agents over two fully active agents. In most cases there are numerically greater virological responses in persons who received three versus two fully active agents. However, the differences are small and in some cases not statistically significant. Seventy-three percent of those who received three active novel agents (enfuvirtide, etravirine and darunavir) achieved a viral

load of < 50 copies/ml at 24 weeks compared with 68% of participants who received two active novel agents (enfuvirtide and darunavir). This difference was not statistically significant [32].

Similarly, the BENCHMRK studies demonstrated that 98% of 44 participants who received enfuvirtide, darunavir and raltegravir achieved a viral load of <400 copies/ml at 24 weeks compared with 87% of the 23 individuals who received enfuvirtide and darunavir but not raltegravir as novel agents [33]. Given the small numbers who received three fully active drugs it is impossible to draw firm conclusions from these studies relating to the benefit of three versus two fully active agents. In addition, we propose in this study to provide two agents drawn from separate ARV classes to which people have not been previously exposed, which differs from the situation described in the late salvage studies described above in which participants received various mixtures of new classes in addition to new agents from classes to which the patient had previously been exposed.

A separate reason for examining a dual combination of drugs from two new classes of ART in second-line therapy is that it is consistent with the simplified public health approach for the use of antiretroviral therapy. This strategy is generally agreed to be the only feasible option for the antiretroviral roll-out effort across resource-limited settings worldwide [24]. The currently recommended approach for second-line ART is inconsistent with public health principles in that the advice about optimising the N(t)RTI combination is vague and complicated. In response to this concern WHO has published a document which prioritises two 2N(t)RTI combinations according to whether thymidine-analogues (two high priority dual combinations) or non thymidine analogues (two high priority dual combinations) were used in the first-line combination regimen. The document also allows for the use of two specific 3N(t)RTI combinations as support for the boosted-PI in second-line ART based on theoretical considerations for which there is however no clinical efficacy data [35]. If this proposed study were to demonstrate non-inferiority with a regimen consisting of ritonavir boosted lopinavir plus raltegravir, then it would be possible to recommend simplified guidelines for both first- and second-line therapy in resource-limited settings.

A final reason that studying a dual combination of drugs from two new classes of ART is attractive is that this strategy spares the continued use of N(t)RTIs in second-line regimens. The N(t)RTIs have been associated with a range of toxicities which are most likely related to the mitochondrial toxicity of N(t)RTIs as a class, albeit with various manifestations depending upon the particular drug(s) administered. The toxicities are many and include myelosuppression, peripheral neuropathy, lactic acidosis, pancreatitis, myopathy, peripheral lipoatrophy, impairment of renal function and metabolic derangements.

1.3 Research hypothesis

In HIV-infected subjects who have virologically failed first-line antiretroviral therapy, treatment with second-line antiretroviral therapy comprising ritonavir-boosted lopinavir and raltegravir provides comparable (i.e. non-inferior) antiretroviral efficacy over 48 weeks to treatment with a regimen comprising ritonavir-boosted-lopinavir and 2-3N(t)RTIs.

2 STUDY OBJECTIVES

2.1 Primary objective

To compare the virological efficacy of the two regimens.

2.2 Secondary objectives

A number of secondary outcomes will be assessed which are of relevance and interest in the assessment of the performance of the two study treatment regimens. These will include (but will not necessarily be limited to) virological, immunological, safety and antiretroviral treatment change.

2.3 Exploratory objectives

Some exploratory objectives will also be examined including clinical, metabolic, anthropometric, medication adherence and quality of life.

3 STUDY POPULATION

3.1 Eligibility criteria

Eligible participants will satisfy all the following inclusion and exclusion criteria within 45 days prior to randomisation, unless otherwise stated. No waivers will be issued. Those with mild to moderate laboratory abnormalities can be re-screened (within the 45 day period) for the abnormal variable if appropriate in the opinion of the Principal Investigator at the site.

3.2 Inclusion criteria

1. HIV-1 positive by licensed diagnostic test
2. Aged 16 years or older (or minimum age as determined by local regulations or as legal requirements dictate)
3. Have received first antiretroviral regimen consisting of an NNRTI plus 2N(t)RTIs for ≥ 24 weeks
4. No change in antiretroviral therapy within 12 weeks prior to screening
5. Failed first-line NNRTI + 2N(t)RTI combination therapy according to virological criteria defined by two consecutive (≥7 days apart) HIV RNA results of >500 copies/mL
6. No prior or current exposure to HIV protease inhibitors and/or HIV integrase inhibitors
7. Able to provide written informed consent

3.3 Exclusion criteria

1. The following laboratory variables
    a) absolute neutrophil count (ANC) <500 cells/µL.

b) hemoglobin <7.0 g/dL

c) platelet count <50,000 cells/µL

d) ALT >5 x ULN

2. Pregnant or nursing mothers
3. Participants with active viral hepatitis B infection defined by the presence in serum of hepatitis B surface antigen
4. Use of immunomodulators within 30 days prior to screening
5. Use of any prohibited medications (rifampicin, midazolam, triazolam, cisapride, pimozide, amiodarone, dihydroergotamine, ergotamine, ergonovine, methylergonovine, astemizole, terfenadine, vardenafil, St. John's wort)
6. Intercurrent illness requiring hospitalisation
7. Active opportunistic disease not under adequate control in the opinion of the investigator
8. Participants with current alcohol or illicit substance abuse that in the opinion of the investigator might adversely affect participation in the study
9. Participants deemed by the investigator unlikely to be able to remain in follow-up for the protocol defined period

3.4 Withdrawal of subjects from study

In general terms, all randomised study subjects should remain in follow-up for the duration of the trial, regardless of whether or not they continue to take randomly assigned therapy. All study subjects should continue to attend all study visits and complete all study-mandated assessments.

Subjects MUST be discontinued from study therapy AND withdrawn from the study for the following reasons:

- Withdrawal of informed consent (subject's decision to withdraw for any reason).
- Termination of the study by the Protocol Steering Committee.

4 TRIAL DESIGN

4.1 Study design

This study is a Phase IIIB/IV, randomised, open label comparison of two independent regimens of combination antiretrovirals as second-line therapy following confirmed virological failure of a first-line NNRTI/2N(t)RTI combination ART regimen. Eligible participants will be randomly allocated to receive one of the two study regimens.

The study will continue for 96-weeks but the primary analysis will take place using the week 48 time point. A total of 550 subjects will be recruited over approximately 50 selected sites in Australia, New Zealand, Africa, Asia, Latin America and Europe.

4.2 Criteria for evaluation

4.2.1 Primary endpoint

To compare the virological efficacy of the two regimens as measured by the proportion of participants with HIV RNA <200 copies/mL 48-weeks and 96 weeks after randomisation in the intention-to-treat (ITT) population*.

*See definition of the ITT and other analysis populations in the analysis plan (Section 11.3)

(note: all sites will have plasma stored at each visit (except screening visit) that will be analysed retrospectively by a central laboratory for HIV-RNA testing).

4.2.2 Secondary endpoints

A number of secondary endpoints will be examined at week 48 and 96 in this protocol by randomised treatment arm. These will include, but not be limited to the following:

Virological endpoints

- an analysis of the time to loss of virological response (TLOVR)
- proportions of participants with <50 and <400 HIV RNA copies /mL
- time to plasma HIV RNA <200 copies/mL
- time to treatment failure (>200 copies/mL)

Immunological endpoints

- mean change in CD4+ cell count from baseline

Safety

- total number of participants with any serious adverse events (SAEs), and the cumulative incidence of SAEs
- total number of participants with any adverse event(s) (AEs) and the cumulative incidence of all AEs
- total number of participants with any adverse event (AE), and cumulative incidence of all AEs associated with cessation of randomly assigned therapy

Antiretroviral treatment

- analyses in which participants who change ART for any reason are classified as having failed randomised treatment
- time to change in randomly assigned therapy (all reasons individually and on aggregate)

4.2.3 Exploratory endpoints

Clinical

- total number of AIDS events and deaths
- total number of serious non-AIDS events*

Metabolic and anthropometric

- mean/median changes from baseline in fasted lipids (TC, LDL-c, HDL-c, and TG), insulin and blood glucose
- changes in body morphology as determined by waist and hip measurements

Resistance

- patterns of genotypic resistance associated with virological failure

Adherence

- adherence and virological outcomes (using the CPCRA instrument)

Quality of Life

- quality of life assessment using the SF-12 instrument

*Serious non-AIDS defining events will be defined as follows (refer to Appendix 4 for details on diagnostic criteria):

- acute myocardial infarction
- coronary artery disease requiring drug treatment
- coronary revascularisation
- congestive heart failure
- deep vein thrombosis
- peripheral arterial disease
- pulmonary embolism
- stroke
- end-stage renal disease (ESRD)
- decompensated liver disease
- non-AIDS-defining cancers
- diabetes mellitus

For all endpoints (primary, secondary and exploratory), plans describing all proposed analyses will be prepared in advance of database lock for each defined interim and final evaluation.

5 TREATMENT OF SUBJECTS

5.1 Treatment group assignment

Eligible participants will be randomised in equal proportions to one of two regimens of combination ART:

I. ritonavir boosted lopinavir (LPV/r) 200mg/50mg 4 tabs once daily or 2 tabs twice daily (OR 133.3mg/33.3mg 6 soft gelatin capsules once daily or 3 soft gelatin capsules twice daily) + 2-3N(t)RTIs

II. ritonavir boosted lopinavir (LPV/r) 200mg/50mg 4 tabs once daily or 2 tabs twice daily (OR 133.3mg/33.3mg 6 soft gelatin capsules once daily or 3 soft gelatin capsules twice daily) + raltegravir 400 mg 1 tablet twice daily

Randomisation will be conducted via the electronic case report form. Specific details regarding randomisation will be provided in the study Manual of Operations (MOOP). Randomisation will be stratified for the following variables:

1. Clinical site
2. Plasma HIV-1 RNA viral load < or ≧100,000 copies/mL

5.2 Study drugs

5.2.1 Raltegravir

Raltegravir is an HIV integrase strand-transfer inhibitor licensed for use as part of combination ART in HIV-infected subjects. Tablets are pink, oval-shaped, film-coated tablets with "227" on one side. One 400mg tablet is taken orally, twice daily with or without food. Tablets will be dispensed from local pharmacies in bottles containing 60 tablets. The bottles must be stored at 20-25°C (68-77°F).

5.2.2 Ritonavir boosted lopinavir

Ritonavir boosted lopinavir is a co-formulation of one protease inhibitor and a pharmaco-enhancer licensed for use as part of combination ART in HIV infected subjects. Ritonavir boosted lopinavir is available in two different forms (‘Kaletra’ and ‘Aluvia’) depending upon the country in which it is marketed. The ‘Kaletra’ form comes as two formulations: (1) yellow, film-coated, ovaloid tablets embossed with the Abbott logo on one side and ‘KA” on the other, (2) orange, soft gelatin, liquid filled capsules. The ‘Aluvia’ form comes as red, ovaloid, film-coated tablets embossed with the Abbott logo on one side and ‘AL’ on the other. Two 200 mg lopinavir/50 mg ritonavir tablets (i.e. 400 mg lopinavir/100 mg ritonavir) should be taken twice daily or four 200 mg lopinavir/50 mg ritonavir tablets (i.e. 800 mg lopinavir/200 mg ritonavir) should be taken once daily with or without food. Three 133.3 mg lopinavir/33.3 mg ritonavir capsules should be taken twice daily or six 133.3 mg lopinavir/33.3 mg ritonavir capsules should be taken once daily with or without food. The tablets and capsules should be swallowed whole and not chewed, broken, or crushed. The choice of once or twice daily dosing is the decision of the treating physician in discussion with the participant. As described in the background section of this protocol,

once daily dosing has now been shown to be virologically equivalent to twice daily. Tablets and capsules will be dispensed from local pharmacies in bottles containing 120 tablets and 180 capsules. The tablet bottles must be stored at 20-25°C (68-77°F) and capsule bottles at 2-8°C (36°F - 46°F). Ritonavir boosted lopinavir will not be provided to Australian sites, as it is ready available via the local healthcare system. In Australia the cost associated with provision of the drug will be covered by NCHECR.

5.2.3 Drug distribution

Drug will be distributed through central ALMAC depots in the following countries:

- USA - will ship directly to one clinical site in Australia, New Zealand, and Mexico. Drugs will be internally transported if there are more than one clinical site within the country
- Ireland- will ship directly to sites in England, Ireland, France, Israel, Germany, Nigeria, South Africa, and India
- Singapore - will ship directly to sites in Singapore, Malaysia, Hong Kong, Taiwan and Thailand
- Argentina
- Peru
- Chile

5.3 Genotypic resistance testing

This trial will be conducted in high, medium, and low income settings, in which genotyping will be variably available. As such, the approach to HIV drug resistance testing during screening will be as follows: genotyping will be allowed but will be optional. Sites wishing to guide the selection of the N(t)RTI backbone according to genotypic resistance testing must have local access to the technology and specify its use to construct the N(t)RTI backbone before the study is implemented. Once a site elects to conduct genotypic HIV drug resistance testing, it must do so for all participants enrolled. However, regardless of this specification, all participants will have a baseline (week 0) sample collected and stored for retrospective analysis of HIV resistance genotype which will be analysed at a single centralised site. This information will then be used in a pre-specified analysis of responses according to genotype.

5.4 Backbone N(t)RTI selection

At sites in which genotypic HIV drug resistance testing is performed, N(t)RTI selection will be the decision of the treating clinician based upon the result. At sites in which such testing is not available or at sites which have access to genotyping but elect not to use this option for selection of the N(t)RTI backbone, the following recommendations are made with regard to selection of N(t)RTIs in the control arm (arm I):

- If first-line N(t)RTIs included zidovudine (AZT) or stavudine (d4T) with lamivudine (3TC) or emtricitabine (FTC), the two second line N(t)RTIs should consist of:

1. tenofovir disoproxil fumerate (TDF) and
2. 3TC or FTC with or without
3. zidovudine (AZT)

- If first-line N(t)RTIs consisted of didanosine (ddI), tenofovir disoproxil fumerate (TDF), or abacavir (ABC) with lamivudine (3TC) or emtricitabine (FTC), the two second line N(t)RTIs should consist of:

  1. zidovudine (AZT) or stavudine (d4T) and
  2. 3TC or FTC with or without
  3. abacavir (ABC)

If uncertainty exists about the selection of the most appropriate N(t)RTI backbone, investigators are advised to refer to the WHO recommendations on antiretroviral therapy for HIV-infection in adults and adolescents in resource-limited settings (http://www.who.int/hiv/pub/guidelines/adult/en/index.html).

The decision of whether to incorporate either two or three N(t)RTIs into the nucleoside/nucleotide backbone of the control arm is up to the investigator. The use of 3N(t)RTIs in the backbone of the second line regimen is based mainly on theoretical considerations; the clinical efficacy of this strategy is unproven [5].

The WHO recommendations on antiretroviral therapy for HIV-infection in adults and adolescents in resource-limited settings states that the combination of TDF and ddI should be used with caution and, with an adjustment of ddI dose according to body weight [5]. From the point of view of the SECOND-LINE protocol the combination of TDF with ddI is considered not recommended, based on data that has suggested unsatisfactory immune reconstitution, comparatively high rates of virological failure and a suggestion of an increased risk of N(t)RTI-associated toxicity using this backbone N(t)RTI combination [36-38].

The use of AZT combined with d4T and 3TC combined with FTC is absolutely contraindicated.

The regimen of raltegravir and 2NRTIs is not permitted in this study.

In the SECOND-LINE protocol the research sites will locally procure nucleoside/nucleotide reverse transcriptase inhibitor (N(t)RTI) antiviral agents for use as components of the combination antiretroviral regimen used in the control arm of the study. NCHECR will reimburse the costs of the N(t)RTIs used in the study.

For all participating research sites NCHECR mandates the use of innovator nucleoside/nucleotide reverse transcriptase inhibitor agents if locally available.

In situations in which innovator nucleoside/nucleotide reverse transcriptase inhibitor agent are not locally available or are in short supply, the use of alternative generic products will be allowed. However, the generic alternatives will be limited to only those products which have received pre-approval through the agency of the World Health Organization (WHO) and/or the US Federal Drug Authority (FDA).

Research sites in which generic formulations may be used will be asked to specify before study commencement the WHO and/or FDA approved generic formulations of choice. This list will be updated on an annual basis.

The pre-approved nucleoside/nucleotide reverse transcriptase inhibitor products can be found at the respective WHO and FDA websites:

WHO: http://apps.who.int/prequal

FDA: http://www.fda.gov/InternationalPrograms/FDABeyondOurBordersForeignOffices/AsiaandAfrica/ucm119231.htm

5.5 Guidelines for clinical management of ART regimen failure on study

This section deals with the clinical management of a series of clinical scenarios which may arise during the course of the study and which require a clinical response.

It is emphasised that these scenarios and suggested responses relate to clinical management issues and **ARE INDEPENDENT** of the analyses of the primary and secondary study endpoints.

Failure of the regimens in this study from the perspective of clinical management (independent of the study endpoints) will be defined as any (one or more) of the following:

1. failure to attain a plasma HIV RNA level <500 copies/mL within the first 24-weeks following commencement of randomised therapy
2. recrudescence of plasma HIV RNA to >500 copies/mL on 2 consecutive occasions ≥7 days apart
3. WHO stage IV HIV diseases progression event (not deemed by the investigator to be a result of immune restoration disease [IRD]) – see section 5.10 for IRD definition
4. Failure of the absolute CD4 T-cell count to rise from baseline value following 24-weeks of continuous randomised therapy
5. Adverse events experienced that result in discontinuation of study drug (see section 5.6 for further details).

Participants failing the regimen according to the above criteria should be managed as considered clinically appropriate. Participants will be encouraged to remain in clinical follow-up and have blood drawn according to the study protocol schedule.

From the point of view of individual patient management investigators may elect to continue participants on the same regimen if, in their judgement the patient continues to benefit on virological, and/or immunological and/or clinical grounds.

In the case of virological failure of the control regimen (LPV/r + 2-3N(t)RTI) it is likely that the boosted-lopinavir will retain activity and therefore participants will be allowed to replace the 2-3N(t)RTI component with raltegravir and continue the LPV/r component. It is emphasised that under this clinical scenario (virological failure of the control regimen) participants should NOT switch the LPV/r to raltegravir and continue on N(t)RTIs as this is associated with an increased risk of virological failure [39].

In the case of virological failure of the experimental arm, management will be at the discretion of the investigator but may include continuation of the same regimen, partial or complete regimen change (e.g. ceasing raltegravir and replacing with 2-3(t)RTIs) or simplification to LPV/r monotherapy. The use of resistance genotypic testing is advised if local testing is available, as well as reference to the results of the screening resistance test. For sites not using resistance testing at screening, reference to Section 5.4 of this protocol (Backbone N(t)RTI selection) is recommended for selection of a suitable N(t)RTI combination.

5.6 Management of ART intolerance

Management of intolerance to one or more of the antiretrovirals will be at the discretion of the investigator and will depend upon the local availability of alternatives. In the case of intolerance attributed to LPV/r a switch to an alternative boosted-PI is advised. If intolerance is thought to be due to one of the N(t)RTIs, an alternative N(t)RTI is recommended (e.g. AZT to d4T for anaemia, TDF to ddI for renal toxicity, d4T to AZT for peripheral neuropathy, ddI to TDF for peripheral neuropathy or pancreatitis).

In all such situations the study will provide material support for switch to alternative agents sourced locally at each site.

5.7 Dose modifications

It is recommended that no dose modifications be employed in response to observed toxicities or failure to achieve or maintain a clinical response. Toxicities and unacceptable treatment responses should be managed as guided in Sections 5.10 and 5.11.

5.8 Toxicity management

Limited pathology will be collected for analysis as part of this Protocol. Each investigator must ensure that safety pathology is collected and reviewed, in keeping with local standards of care.

Clinical events and clinically significant laboratory abnormalities will be graded according to the DAIDS Common Toxicity Grading Scale (please refer to Appendix B of the Manual of Operations [MOOP]).

5.8.1 Grade 1 or 2 toxicity

Subjects who develop a Grade 1 or 2 adverse event or toxicity may continue study drugs without modification.

Subjects experiencing Grade 1 or 2 adverse events who choose to discontinue the study drugs should remain on study and continue to undergo protocol-mandated evaluations and assessments.

5.8.2 Grade 3

If the investigator has compelling evidence that the adverse event has NOT been caused by the study drug(s), dosing may continue.

Subjects who develop a Grade 3 adverse event or toxicity, except for elevated triglycerides (Section 5.10.1), glucose (Section 5.10.2) and AST/ALT (Section 5.10.3) should have their antiretroviral study drugs withheld, at the investigator's discretion.

The subject should be re-evaluated regularly until the adverse event returns to Grade ≤2, at which time the study drugs may be reintroduced at the discretion of the investigator or according to standard practice.

If the same Grade 3 adverse event recurs within four weeks, study drug(s) must be permanently discontinued if the investigator considers the adverse event related to study drug(s). However, if the same Grade 3 adverse event recurs after four weeks, the management scheme outlined above may be repeated.

Subjects experiencing Grade 3 adverse events requiring permanent discontinuation of study drug therapy should be followed regularly (weekly is suggested) until resolution of the adverse event. Participants should remain in follow-up and continue to attend for protocol-mandated assessments and evaluations. See Section 5.4 for permitted drug substitutions.

5.8.3 Grade 4 adverse events

Subjects who develop a Grade 4 adverse event or toxicity will have all study drug(s) temporarily discontinued (except for those abnormalities described in Sections 5.10.1 – 5.10.4 inclusive). The AE should be resolved before any decision is made to rechallenge the subject with study drugs. Subjects experiencing Grade 4 AEs requiring permanent discontinuation of study drug therapy should be followed regularly (weekly is suggested) until resolution of the adverse event. The patient should remain on study and continue to undergo protocol-specified evaluations and assessments.

Subjects with Grade 4 asymptomatic or non-significant laboratory abnormalities may continue study drug therapy if the investigator has compelling evidence that the toxicity is NOT related to the study drug(s).

5.9 Protocol-specific toxicity management guidelines

Certain clinical and laboratory abnormalities will be subject to the following guidelines under this protocol. These guidelines are provided to encourage uniformity of patient management in light of observed clinical and/or laboratory abnormalities that are well documented as expected adverse experiences following administration of study drugs. Departure from these guidelines is discouraged.

5.9.1 Hypertriglyceridaemia/Hyperlipidaemia

If elevated triglyceride or lipid levels are from a non-fasting blood draw, the test should be repeated on a specimen drawn after a minimum eight hour fast. Only levels done in a fasting state (8-hour minimum) should be graded for toxicity.

Subjects with asymptomatic Grade ≥3 triglyceride, total cholesterol, or LDL elevations may continue study medications at the discretion of the investigator. Appropriate dietary modification and lipid lowering therapy should be considered. Thought should also be given to modification of other existent cardiovascular disease (CVD) risk factors (e.g. sedentariness, overweight/obese, tobacco smoking). The preferred first line pharmaceutical intervention is gemfibrozil 600 mg q 12 hours, taken 30 minutes prior to the morning and evening meals. Fasting triglycerides should be rechecked at regular (suggest at least monthly) intervals. For persistent uncontrolled hypertriglyceridaemia and/or hypercholesterolemia, the addition of niacin and/or an HMG-CoA reductase inhibitor (i.e. 'statin') may be considered. These medications should be introduced with caution (see Section 6.3). Niacin has the propensity to worsen the control of blood sugar in subjects with diabetes mellitus or a history of hyperglycaemia. Several HMG-CoA reductase inhibitors have substantial interactions with the PI class. The use of pravastatin or atorvastatin is advised. Rosuvastatin should be used with care (please refer to table 5.1 under section 5.13 in this protocol for further details).

5.9.2 Hyperglycaemia

Subjects with Grade ≥3 hyperglycaemia may continue study medications at the discretion of the investigator and be managed with oral hypoglycaemic medications or insulin, with referral to endocrinologists as appropriate.

5.9.3 Hepatitis/Hepatotoxicity

For Grade 3 elevation in AST or ALT (5.1-10.0 x ULN), study medications may be continued at the discretion of the site investigator. Careful assessments should be performed to rule out alcohol-related hepatitis, non-study medication-related drug toxicity, or viral hepatitis (e.g. flare of disease activity, acute infection, super-infection or IRD) as the cause of the Grade 3 elevation. The possibility of mitochondrial toxicity/hepatic steatosis/lactic acidosis syndrome should also be explored in participants in the control arm receiving N(t)RTIs.

For Grade 4 elevations (>10 x ULN) in AST or ALT, all study medications should be withheld until the toxicity grade returns to Grade $\leq$2.

5.9.4 Lactic acidosis

The relevance of asymptomatic lactic acid elevations is unclear, and routine collection of lactates is not recommended and will not form part of the routine safety evaluations for this study. Routine lactate monitoring is not currently recommended.

The syndrome of lactic acidosis is sometimes fatal (particularly if recognised late) and is often associated with evidence of hepatic steatosis. It is a recognised but nevertheless uncommon complication of N(t)RTI therapy. This syndrome is thought to be secondary to mitochondrial toxicity induced by the inhibitory effect of N(t)RTIs on DNA polymerase gamma, a key enzyme needed for mitochondrial DNA synthesis. Current knowledge regarding this syndrome is not complete, although female sex, obesity and prolonged N(t)RTI exposure may be risk factors. The clinical features of lactic acidosis frequently involve non-specific symptoms such as fatigue, weakness, and fever; the majority of cases also involve symptoms suggestive of hepatic dysfunction such as nausea, vomiting, abdominal or epigastric discomfort, abdominal distension, hepatomegaly, and elevation of liver enzymes (often to only mild to moderate levels). A high index of suspicion is required to diagnose this condition. Alternatively, unwarranted concern may be raised by over interpretation of lactate levels, particularly if the participant is asymptomatic. N(t)RTI toxicity is only one cause of lactic acidosis. Type "B" lactate elevations or those without clinically apparent tissue hypoxia are also seen in the context of diabetes mellitus, uraemia, liver disease, infections, malignancies, alkaloses, drug and/or toxin ingestion of such substances as ethanol, methanol, ethylene glycol and salicylates.

5.9.5 Renal toxicity

Routine assessment of renal function is required with derivation of creatinine clearance (CL_{cr}) using the Cockcroft-Gault equation. Dosing of all medications in a patient's regimen should be critically reviewed if the estimated creatinine clearance falls below 50mls/minute.

Glomerular filtration rate will also be estimated using the abbreviated MDRD equation. Estimated GFR = 186 x ($[S_{CR}/88.4]^{-1.154}$) x $age^{-0.203}$ x (0.742 if female) x (1.210 if African-American). Dosing of all medications in a patient's regimen should be critically reviewed if the eGFR falls below 60ml/min/1.73m^2.

It is important to note that recommendations regarding dose adjustments in the presence of renal impairment are based on calculations of renal function using the Cockcroft-Gault equation.

5.10 Management of AIDS Defining Illnesses (ADI) and Immune Restoration Disease (IRD)

In general, management will follow relevant local and international guidelines. Any episode of an ADI on study will be regarded as a study failure according to CDC criteria (Appendix 1), unless the episode is thought to represent immune restoration disease (IRD). The protocol and Manual of Operations will contain guidelines for the differentiation of ADI and IRD.

For the purposes of this study the definition of IRD will follow a modification of the proposed criteria suggested by French, Price and Stone as follows [40]:

A. Atypical presentation of opportunistic infections or tumours in participants responding to antiretroviral therapy (ART)

I Localised disease

II Exaggerated inflammatory reaction

III Atypical inflammatory response in affected tissues

IV Progression of organ dysfunction or pre-existing lesions after definite clinical improvement with pathogen-specific therapy prior to commencement of ART and exclusion of treatment toxicity and new diagnoses.

B. Decrease in plasma HIV RNA level by $>1\log_{10}$copies/mL

To qualify for a diagnosis of IRD participants would be required to demonstrate at least one of criterion A (AI – AIV) and criterion B [40].

In the case of an episode of new or recurrent pulmonary tuberculosis (PTB) this will not be counted as evidence of HIV disease progression. In the case of PTB, treatment will be according to standard recommendations with the caveat that participants will have access to rifabutin under the auspices of the study in place of rifampicin as the rifamycin for inclusion in combination anti-TB therapy. Rifabutin will be dose-reduced in the presence of a protease inhibitor according to guidelines. In this instance, participants may remain on their randomised regimen.

5.11 Discontinuation of therapy

Study therapy MUST be immediately discontinued for the following reasons:

- any clinical adverse event, laboratory abnormality or intercurrent illness that, in the opinion of the investigator, indicates that continued treatment with study therapy is not in the best interest of the subject

- termination of the study by the Protocol Steering Committee
- withdrawal of consent by the participant

Women found to be pregnant should be managed in keeping with the provisions in Section 8.2.

Subjects discontinuing their study drugs should remain in follow-up and be encouraged to continue protocol-mandated evaluations and assessments.

5.12 Treatment adherence

Study drug adherence will be assessed using the CPCRA (Community Programs for Clinical Research on AIDS) questionnaire. For full details on this adherence tool please refer to the Manual of Operations (MOOP). All participants should be counselled regularly on the need for maintaining strict adherence with the allocated study regimens. The objective should be 100% adherence at all times during follow-up. The site Principal Investigator is responsible for assessing adherence with all aspects of the study including use of study drugs and attendance at protocol-mandated clinical visits and assessments.

5.13 Prohibited and restricted therapies during the study

The following medications are prohibited during the study: rifampicin, midazolam, triazolam, cisapride, pimozide, amiodarone, dihydroergotamine, ergotamine, ergonovine, methylergonovine, astemizole, terfenadine, vardenafil, St. John's wort and immunomodulators.

Investigational drugs other than those included in the assigned regimens are prohibited in this protocol.

Systemic cytotoxic chemotherapy is prohibited in this protocol. If a participant requires chemotherapy whilst on study follow-up, please contact NCHECR.

Caution should be used when coadministering Raltegravir with strong inducers of uridine diphosphate glucuronosyltransferase (UGT) 1A1 (e.g. rifampicin which is prohibited in this protocol) due to reduced plasma concentrations of raltegravir. Other less stronger inducers (e.g., rifabutin) may be used.

Drugs known to interact with HIV protease inhibitors in ways that may require therapeutic drug monitoring and/or adjustment of concomitant medication are listed in Table 5.1 below.

Table 5.1 Significant drug interactions requiring dosage alteration or alterative drugs (LPV)

-

LIPID-LOWERING AGENTS	Avoid simvastatin, lovastatin with LPV. Use pravastatin, fluvastatin or rosuvastatin instead.
IMMUNOSUPPRESSANTS	Caution when using cyclosporine, sirolimus and tacrolimus with LPV, as they may increase levels of immunosuppressants.

STEROIDS	Caution when using steroids like dexamethasone, fluticasone, propionate and ethinyl oestradiol.
HEART MEDICINE	Caution using calcium channel antagonistas like felodipine, nifedipine and nicardipine with LPV.<br>Caution using bepridil, systemic lidocaine and quinidine with LPV.<br>Caution using digoxin with LPV.
ANTI-INFECTIVES	Caution using certain antibiotics like rifabutin and clarithromycin with LPV
ANTI-CONVULSANTS	Caution using anti-convulsants like carbamazepine, phenytoin and phenobarbital with LPV
ANTI-DEPRESSANTS	Caution using antidepressants like trazodone with LPV
ANTI-FUNGALS	Caution using antifungals like ketoconazole and itraconazol with LPV
MORPHINE LIKE MEDICINE	Caution using drugs like methadone with LPV.
ERECTILE DYSFUNCTION	Caution with erectile dysfunction drugs (sildenafil and tadalafil) with LPV as dose of ED drug can be increased and may need dose reduction.
SEX STEROIDS	Caution using oral contraceptives with LPV as sole method of contraception; unreliable.

For further information on HIV drug interactions please refer to the website:
http://www.hiv-druginteractions.org

5.14 Concomitant Medication

For this study we will only be collecting information on concomitant medications that are prescribed by a physician.

6 STUDY PROCEDURES

6.1 Initial screening period

Potentially eligible individuals can be screened within 45 days before the randomisation visit and all results from screening must be available for randomisation. All subjects should be given adequate information about the trial including the Participant Information Sheet and be given an opportunity to ask questions about the trial. Written consent for the trial should be obtained at the screening visit before any protocol specified assessments are performed. The following evaluations will be performed within 45 days prior to randomisation:

6.1.1 Clinical assessments

- Complete medical history to include

  - non-HIV related diagnosis including liver disease, renal disease, cardiovascular disease, diabetes, hypertension, pancreatitis, bone disorders, rheumatoid arthritis, non-AIDS defining cancer, non-traumatic fracture and substance abuse
  - smoking history
- family history of diabetes or hip fracture
- race and gender at birth
- measure weight, sitting blood pressure and pulse
- informed consent

6.1.2 Laboratory assessments

- If the patient is female and is of child bearing potential, she must have a urine or blood sample pregnancy test
- serum chemistries and liver function tests: electrolytes (sodium, potassium, bicarbonate, chloride, phosphate), urea, creatinine, LFTs (total bilirubin and ALT), anion gap (automatically calculated on the eCRF) and creatinine kinase
- Haematology: full blood count (haemoglobin, white cells, neutrophils, lymphocytes, platelets)
- creatinine clearance will be calculated by the Cockcroft-Gault equation

Male: $$\frac{(140 - \text{age in years}) \times (\text{wt in kg})}{0.814 \times (\text{serum creatinine in } \mu\text{mol/L})} = CL_{Cr} \text{ (mL/min)}$$

Female: $$\frac{(140 - \text{age in years}) \times (\text{wt in kg})}{0.814 \times (\text{serum creatinine in } \mu\text{mol/L}).} \times 0.85 = CL_{Cr} \text{ (mL/min)}$$

- glomerular filtration rate (GFR) will be calculated by the abbreviated MDRD (Modification of Diet in Renal Disease) equation.

$$\text{Estimated GFR} = 186 \times ([S_{CR}/88.4]^{-1.154}) \times \text{age}^{-0.203} \times (0.742 \text{ if female}) \times (1.210 \text{ if African-American})$$

- real time plasma HIV-RNA analysis at local laboratory
- Immunology: CD4 and CD8 (% and absolute)
- Hepatitis B surface antigen
- Hepatitis C antibody results need only be done if status is unknown, or previous tests were negative or reports for positive results are not available to verify the result
- HLA-B*5701 testing can be conducted if locally available (costs will be reimbursed)
- For those sites which have nominated as sites performing genotype testing as part of the study a local genotype should be collected at screening to assist with N(t)RTI selection at randomisation if participant is randomised to that arm*

*The site must advise NCHECR if they wish to guide the N(t)RTI backbone by conducting genotypic resistance testing prior to the randomisation visit. Once the site elects this option it must be followed for all enrolled participants.

6.2 Randomisation visit

To proceed, all subjects must have fulfilled the eligibility criteria by the results of evaluations at screening. All randomisation evaluations must be completed prior to the commencement of study drug.

Trial entry procedures will include:

6.2.1 Clinical assessments

- targeted physical examination (symptom directed)
- height, weight, sitting blood pressure and pulse
- symptoms reported by the subject will be reviewed by the clinician at each visit and recorded on the CRFs as adverse events
- full HIV history to include: mode of transmission, duration of HIV infection, antiretroviral treatment history, nadir CD4+ cell count, highest recorded HIV RNA, and current stage of HIV disease and any prior AIDS events (CDC classification – refer Appendix 1)
- anthropometric assessments: umbilical waist and maximum hip measurement (refer to MOOP for instructions on how to perform this measurement)
- updated medical history including changes or additions to diagnoses, diseases, or any change in antiretroviral drugs, concomitant medications
- complete Short Form 12 (SF12) quality of life questionnaire
- collection of current prescribed concomitant medications

6.2.2 Laboratory assessments

- If the patient is female and is of child bearing potential, they must have a urine or blood sample pregnancy test
- serum chemistries and liver function tests: electrolytes (sodium, potassium, bicarbonate, chloride, phosphate), urea, creatinine, LFTs (total bilirubin and ALT), anion gap (automatically calculated on eCRF) and creatinine kinase
- Fasting metabolic parameters (minimum 8 hours): total cholesterol, HDL cholesterol, LDL cholesterol, triglycerides, glucose, HOMA and insulin.
- Haematology: full blood count (haemoglobin, white cells, neutrophils, lymphocytes, platelets)
- creatinine clearance will be calculated by the Cockcroft-Gault equation, as above
- estimated glomerular filtration rate will be calculated by the MDRD equation, as above
- real time plasma HIV-RNA analysis at local laboratory
- Immunology: CD4 and CD8 (% and absolute)
- storage plasma, serum and PBMC for possible future investigations related to HIV disease
- Plasma storage for central HIV RNA and genotype analysis

6.2.3 Drug supply and randomisation

After completion of all randomisation study visit assessments the participant will be randomised online via the web based CRF. Eligibility must be confirmed and the participant will then be automatically randomised to commence either:

I. ritonavir boosted lopinavir (LPV/r) 200mg/50mg 4 tabs once daily or 2 tabs twice daily (OR 133.3mg/33.3mg 6 soft gelatin capsules once daily or 3 soft gelatin capsules twice daily) + 2-3N(t)RTIs
II. ritonavir boosted lopinavir (LPV/r) 200mg/50mg 4 tabs once daily or 2 tabs twice daily (OR 133.3mg/33.3mg 6 soft gelatin capsules once daily or 3 soft gelatin capsules twice daily) + raltegravir 400 mg 1 tablet twice daily

The treatment assignment will be stratified according to:

1. Clinical site
2. Plasma HIV-1 RNA viral load < or ≥100,000 copies/mL

The investigator should complete a prescription for the randomised regimen and the participant should fill the prescription that same day or the next. Therefore, the study drug should be commenced as soon as possible after randomisation.

6.3 Follow-up visits

The scheduled date of study visits will be calculated from the date of randomisation (day 0, week 0). A schedule of the dates for study visits will be automatically provided for each participant on the eCRF, once randomisation has been completed. For study visit week 4, the actual visit can be from week 2 to week 6. For study visits week 12 and onwards the actual visit can be 6 weeks either side of the scheduled visit date.

NOTE: Subjects must be in a fasting state, but encouraged to take free fluids at weeks 4, 12, 24, 48, 72, and 96.

6.3.1 Clinical assessments

- targeted physical examination (symptom directed) [all visits]
- symptoms reported by the subject or symptoms identified after examination will be reviewed by the clinician at each visit and recorded on the CRFs as adverse events [all visits]
- updated medical history including changes or additions to diagnoses, diseases, or any change in antiretroviral drugs, prescribed concomitant medications [all visits]
- measure vital signs: weight, sitting blood pressure and pulse [all visits]
- anthropometric measurements including waist and hip measurements [week 48 and 96] (refer to MOOP for instructions on how to perform this measurement)
- complete the CPCRA adherence questionnaire [weeks 4, 48, 96]
- complete SF12 quality of life questionnaire [week 48 and 96]

6.3.2 Laboratory assessments

- Pregnancy test if the patient is female and of child bearing potential [all visits]
- haematology - full blood count (haemoglobin, white cells, neutrophils, lymphocytes, platelets) [all visits]
- serum chemistries and liver function tests: electrolytes (sodium, potassium, bicarbonate, chloride, phosphate), urea, creatinine, LFTs (total bilirubin and ALT), anion gap (automatically calculated on the eCRF and creatinine kinase [all visits].
- creatinine clearance will be calculated by the Cockcroft-Gault equation as above [all visits]
- estimated glomerular filtration rate will be calculated by the abbreviated MDRD equation as above [all visits]
- fasting parameters (total cholesterol, HDL cholesterol, LDL cholesterol, triglycerides, glucose, HOMA and insulin) [all weeks with the exception of weeks 36, 60 and 84]
- Immunology: CD4 and CD8 (% and absolute) [all visits]
- real time plasma HIV-RNA analysis [all visits], tested at local laboratory.
- plasma storage for central HIV RNA analysis [all visits]
- storage plasma and serum [all weeks].

General comments

- The window period for each study visit is continuous so that all collected results may be utilised.
- If a scheduled visit is conducted after the window period has ceased for that specific visit, it will be counted as the next visit, and the previous visit should be documented as "missed". DO NOT conduct a second routine visit in the same window period.

7 DATA SAFETY MONITORING BOARD (DSMB)

An independent DSMB will be established. Clinicians or investigators responsible for the clinical care of study subjects or representatives of Pharmaceutical Companies are not permitted to be members of the DSMB.

The DSMB will monitor the trial and will review efficacy and safety data by blinded treatment allocation (A and B). Safety monitoring will be carried out on two occasions, firstly when 250 (125 in each arm) participants complete the week 24 visit and secondly when all participants complete the week 24 visit. The first analysis will assess virological response data between arms; the second analysis will include laboratory data, Grade 3 and Grade 4 adverse events, viral load data, serious adverse events (SAEs) and adverse events leading to cessation of study medication.

The DSMB will develop a formal stopping rule for early evidence of regimen failure or success. They will make their recommendations to the Protocol Steering Committee who will decide whether to amend the trial.

Separate procedures will be developed to assist the DSMB in these tasks. All data will be reviewed blind to treatment allocation.

8 ADVERSE EVENT REPORTING

In this study we will be collecting all grade (grade 1-4) clinical adverse events. Laboratory abnormalities that occur without related clinical symptoms and signs should not be recorded as an adverse event. If the laboratory event becomes clinically significant, then the resulting clinical event should be reported as an adverse event (e.g. a low haemoglobin should be reported as anaemia).

An AE can be any unfavorable and unintended sign (including a clinically significant abnormal laboratory finding, for example), symptom, or disease temporally associated with the use of a medicinal (investigational or marketed) product, whether or not considered related to that product. AEs include any illness, sign, symptom, or clinically significant laboratory test abnormality that has appeared or worsened during the course of the clinical trial, regardless of causal relationship to the drug(s) under study. Changes resulting from normal growth and development which do not vary significantly in frequency or severity from expected levels are not to be considered adverse experiences. Examples of this may include, but are not limited to, onset of menses or menopause occurring at a physiologically appropriate time.

Adverse experiences may occur in the course of the use of a drug in clinical studies or within the follow-up period specified by the protocol, or prescribed in clinical practice, from overdose (whether accidental or intentional), from abuse and from withdrawal.

Adverse experiences may also occur in screened subjects/participants during any pre-allocation baseline period as a result of a protocol-specified intervention including washout or discontinuation of usual therapy, diet, placebo treatment or a procedure.

Timely and complete reporting of all AEs assists in identifying any untoward medical occurrence, thereby allowing: (1) protection of safety of study subjects; (2) a greater understanding of the overall safety profile of the study drugs; (3) recognition of dose-related study drug toxicity; (4) appropriate modification of study protocols; (5) improvements in study design or procedures; and (6) adherence to worldwide regulatory requirements.

AEs may be either spontaneously reported or elicited during questioning and examination of a subject. All identified AEs must be recorded and described on the appropriate Non-serious or Serious AE (SAE) page of the CRF. If known, the diagnosis of the underlying illness or disorder should be recorded, rather than its individual symptoms.

Subjects experiencing AEs that cause interruption or discontinuation of study drugs, or those experiencing AEs that are present at the end of their participation in the study should receive follow-up as appropriate. If possible, report the outcome of any AE that caused permanent discontinuation or that was present at the end of the study particularly if the AE was considered by the investigator to be certainly, probably, or possibly related to the study drugs.

8.1 Definition and reporting of an overdose

Any overdose, whether or not associated with an adverse experience, must be reported within 24 hours to the NCHECR. An overdose of raltegravir would be an excess of 1200 mg per day (raltegravir).

8.2 Reporting of pregnancy

Sexually active women of childbearing potential must use an effective method of birth control during the course of the study, in a manner such that risk of failure is minimised.

Women found to be pregnant during follow-up should be managed in keeping with prevailing national or international guidelines. If a patient on raltegravir becomes pregnant during the study and there are other appropriate treatment options, it is suggested that they discontinue raltegravir and use an alternative.

All pregnancies on study are subject to expedited reporting on the study, and therefore the serious advise event form will need to be completed and submitted to NCHECR within 24 hours (one working day) of becoming aware of the pregnancy. It is the responsibility of investigators or their designees to report any pregnancy in a subject/patient (spontaneously reported to them) which occurs during the study or within 30 days of completing the study. All subjects/participants who become pregnant must be followed to the completion/termination of the pregnancy. If the pregnancy continues to term, the outcome (health of infant) must also be reported to the NCHECR.

8.3 Adverse events

All adverse events must be recorded on the AE page of the CRF. These should be followed to resolution or stabilisation, and reported as an SAE if they become serious or as an SNAE if they meet the criteria.

Laboratory abnormalities that occur without related clinical symptoms and signs should not be recorded as AEs unless of themselves they represent a clinically significant event.

8.4 Serious non-AIDS events (SNAEs)

Serious non-AIDS defining events will be defined as follows (refer to Appendix 4 for details on diagnostic criteria):

- acute myocardial infarction

- coronary artery disease requiring drug treatment
- stroke
- coronary revascularisation
- congestive heart failure
- deep vein thrombosis
- peripheral arterial disease
- pulmonary embolism
- end-stage renal disease (ESRD)
- decompensated liver disease
- *non-AIDS-defining ca*ncers
- diabetes mellitus

Please note that most of the SNAEs will also be reported as a serious adverse event (SAE) and whether or not a serious adverse event, all SNAEs will require expeditious reporting as outlined in the next section. There is only one paper-based serious event form that needs to be completed if the event is either an SNAE or SAE.

8.5 Serious adverse events (SAEs)

An event that is serious must be recorded on the paper SAE (SAE) form as well as on the AE eCRF and requires expeditious handling to comply with regulatory requirements.

A Serious Adverse Event or reaction is any untoward medical occurrence that at <u>any dose</u>:

- Results in death
- Is life threatening -
  (Defined as an event in which the subject or patient was at risk of death at the time of the event; it does not refer to an event which hypothetically might have caused death if it were more severe.)
- Requires inpatient hospitalisation or prolongation of existing hospitalisation
- Results in persistent or significant disability/incapacity
- Is a congenital anomaly/birth defect
- Is a cancer (a pre-existing cancer need only be reported if after study entry it has worsened)
- Is an overdose (whether accidental or intentional)
- Pregnancy
- Is an important medical event -
  (Defined as a medical event(s) that may not be immediately life-threatening or result in death or hospitalisation but, **based upon appropriate medical and scientific judgement**, may jeopardise the patient/subject or may require intervention (e.g., medical, surgical) to prevent one of the other serious outcomes listed in the definition above). Examples of such events include, but are not limited

to, intensive treatment in an emergency room or at home for allergic bronchospasm; blood dyscrasias or convulsions that do not result in hospitalisation.

Adverse events classified as ‘serious‘ require expeditious handling and reporting to NCHECR to comply with regulatory requirements. Any SAE that, in the opinion of the Investigator, is certainly, probably, possibly, not likely related or unrelated to the study drugs must be immediately reported by telephone, if feasible. Regardless, all SAEs whether related or unrelated to study drugs, must be immediately reported by confirmed facsimile transmission and mailing of the completed SAE page. A facsimile transmission does not preclude mailing of the SAE page. Overnight express mail may be used in lieu of facsimile. If only limited information is initially available, follow-up reports are required.

Any serious adverse experience, including death due to any cause, which occurs to any subject/patient entered into this study or within 30 days following cessation of study drug, whether or not related to the drug product, **must be reported within 24 hours (or one working day) to the NCHECR.**

If the participant is prescribed abacavir and subsequently experiences an “Abacavir Hypersensitivity Reaction” this will need to be reported as a serious adverse event.

Additionally, any serious adverse experience considered by an investigator who is a qualified physician to be possibly, probably, or definitely related to the study drug that is brought to the attention of the investigator, at any time outside of the time period specified in the previous paragraph, also must be reported immediately to the NCHECR. Should the investigator become aware of an SAE (regardless of its relationship to investigational product) that occurs within 30 days after stopping the study drugs, the SAE must be reported in accordance with procedures specified in this protocol. In the event of death, if an autopsy is performed, a copy of the report should be sent to NCHECR.

Upon receiving such notices, the Investigator must review and retain the notice with the Investigator Brochure and immediately submit a copy of this information to the Institutional Review Board (IRB)/Independent Ethics Committee (IEC) according to local regulations. The Investigator and IRB/IEC will determine if the informed consent requires revision. The Investigator should also comply with the IRB/IEC procedures for reporting any other safety information.

Updates by the Investigator to Health Authorities should be handled according to local regulations.

Contact information for Safety Reporting will be provided by the suppliers of the clinical trial material and will be outlined in the Manual of Operations.

8.6 Laboratory test abnormalities

Laboratory test abnormalities will not be recorded as AEs unless there is an associated clinical manifestation, in which case only the clinical sign and symptoms should be recorded on the AE form.

8.7 Other safety considerations

Any clinically significant changes noted during physical examinations, electrocardiograms, x-rays, and any other potential safety assessments, whether or not these procedures are required by the protocol, should also be recorded on the appropriate AE page of the CRF (i.e., NON-SERIOUS or SERIOUS) in order for NCHECR to collect additional information about that abnormality, including information regarding relationship to investigational product or other causes, any action taken and resolution.

9 PACKAGING, LABELLING and ACCOUNTABILITY of TRIAL SUPPLIES

9.1 Study drugs

Study materials will be dispensed in accordance with the random allocation following receipt of prescriptions from recognised study investigators. Study drugs will be dispensed at weeks 0, 4, 12, 24, 36, 48, 60, 72, and 84 plus one more year after that.

There will be no drug accountability in this study so no returned bottles or destruction required.

At each dispensing, participants should be counselled on the importance of adherence.

Raltegravir and LPV/r will be the only study drugs which will be provided centrally in this protocol. As such the study requires that management of raltegravir and LPV/r supplies be subject to a number of important provisions (as shown in the following sections).

Mechanisms will be implemented to secure adequate supplies of all other study drugs in countries where access is constrained by cost. Continued supply of required study drugs will be ensured to the study participants in these countries for a period of no less than one year after completion of the 2 year study (i.e. a minimum of 3 year drug supply will be provided to all study participants). Material support for the cost of the N(t)RTI component of arm I of the randomisation will be provided to the clinical site.

All other study drugs should be handled in keeping with normal procedures for licensed products.

9.2 Packaging and labeling

The raltegravir and ritonavir boosted lopinavir will be packaged and labeled by ALMAC clinical services based in the USA and UK. Each bottle will be labeled either with a single-panel English label or a booklet label containing 13 languages.

Raltegravir will be packaged in bottles containing 60 pink tablets with “227” image, which will be sufficient for 30 days supply.

Ritonavir boosted lopinavir is available in two different forms (‘Kaletra’ and ‘Aluvia’) depending upon the country in which it is marketed. The ‘Kaletra’ form comes as two formulations: (1) yellow, film-coated, ovaloid tablets embossed with the Abbott logo on one side and ‘KA” on the other, (2) orange, soft gelatin, liquid filled capsules. The ‘Aluvia’ form comes as red, ovaloid, film-coated tablets embossed with the Abbott logo on one side and ‘AL’ on the other. Tablets and capsules will be dispensed from local pharmacies in bottles containing 120 tablets and 180 capsules. The tablet bottles must be stored at 20-25°C (68-77°F) and capsule bottles at 2-8°C (36°F - 46°F).

9.3 Handling and dispensing of study drugs

Raltegravir (400 mg tablets) and LPV/r (200mg/50mg tablets or 133.3 mg lopinavir/33.3 mg soft gel capsules) should be stored in a secure area according to local regulations. It is the responsibility of the Investigator to ensure that the study drugs are only dispensed to study participants. The study drugs must be dispensed only from official study sites by authorised personnel according to local regulations.

Once the subject has received a treatment assignment, a 30-day supply will be dispensed to the subject from clinical supplies stored at the study site. A 60- to 90-day supply will be dispensed at the week 4 and subsequent visits. The subject must be instructed to take the tablets whole and to store all medication out of sight and reach of children.

9.4 Study drug records at investigational site(s)

It is the responsibility of the Investigator to ensure that a current record of raltegravir disposition is maintained at each study site where it is inventoried and disposed. Records or logs must comply with applicable regulations and guidelines and should include:

- amount received and placed in storage area
- amount currently in storage area
- label ID number or batch number
- dates and initials of person responsible for each investigational product inventory entry/movement
- amount dispensed to each subject, including unique subject identifiers
- amount transferred to another area for dispensing or storage
- non-study disposition (e.g., lost, wasted, broken)

The Sponsor will provide forms to facilitate inventory control if the investigational site does not have an established system that meets these requirements.

10 BIOLOGICAL SAMPLES

10.1 Blood collection

It is important that the handling of blood samples is undertaken according to local guidelines and regulations for handling infectious substances. The blood tubes required to be used for each test should be as per your local laboratory guidelines. Please check requirements with your local laboratory. Storage samples for HIV RNA, spare plasma and PBMC will be collected. Information on the types of blood tubes required for these specimens are detailed in the Manual of Operations. These samples will be processed locally and then shipped to a central laboratory.

It is preferred that the blood is collected within 14 days of the planned visit date (except the screening visit).

All lipid and glycaemic parameters must be collected in the fasting state defined as no intake of food or beverage for at least 8 hours prior to blood collection (although plain water can be taken ad libitum). Prior to blood collection, subjects must have:

- Followed a regular diet for 3 days
- Undertaken normal physical activity for 3 days but no strenuous physical activity within the previous 24 hours

When blood is taken, subjects must have been sitting for at least 5 minutes. A tourniquet may be applied, but for no longer than 2 minutes.

Ensure all tubes are filled to capacity. Invert all the tubes 5-10 times after filling, DO NOT SHAKE.

All samples should be kept at ROOM temperature until delivered to the laboratory.

10.2 Labeling of specimen tubes

It is important that the blood tubes are labelled accurately and legibly. If your normal procedure is to write directly onto the tube then please ensure you use a permanent marker and all required sections are completed. NCHECR will provide you with specimen labels for you to use if you prefer. These labels are only to be used on the blood tubes to be sent to the laboratory, not to be placed on the aliquot tubes for frozen storage.

10.3 Transportation of samples to pathology

It is important that during the transportation of blood samples precaution is taken according to local guidelines and regulations for handling infectious substances.

You will be required to set up the procedures for transporting the blood tubes to your local laboratory. It is important that the samples arrive at the laboratory within 2-3 hours of blood collection (this time frame is determined by the chemistry, lipids and glucose).

10.4 Storage of samples

Serum and plasma samples will be collected and processed at the local pathology provider. The samples will be stored in the interim at local laboratories and then sent for central storage in Sydney at regular intervals throughout the study. Transport to the central laboratory will be organised by NCHECR. The cost of processing and storage of bloods will be provided to the sites as per the financial agreements. For more detailed information on the types of tubes and processing requirements please refer to the Manual of Operations.

10.5 Processing of samples

All samples will be analysed at the local laboratory. It is important that the handling of blood samples is undertaken according to local guidelines and regulations for handling infectious substances. The investigator may be contacted should the technical condition of the sample, absence of information or inconsistencies on the request form be such that the samples cannot be processed. Demographic errors (i.e. incomplete or inconsistent participant information) will be called through to the site and a faxed confirmation of correct information requested. Where such errors are noted after a report has been sent to the site, then a second corrected report will be reissued by the local laboratory.

10.6 Reporting of results

The results will be reported to you as per your local standard procedure. It is important that once these results are received they are entered into the eCRF and a hardcopy of the results are kept in the medical notes. If you receive the laboratory results electronically, the electronic format is adequate for source data verification.

10.7 Plasma HIV RNA determination

Plasma HIV RNA will be measured at your local laboratory for assessment of eligibility and clinical decisions. Plasma will be stored and sent to a central laboratory for central assessment of HIV RNA. These central results will be used for the analysis of the study at week 24, 48 and 96.

11 STATISTICAL METHODOLOGY

11.1 Sample size determination and statistical analysis

Approximately 550 participants will be enrolled.

The primary endpoint is the comparison of proportions of people in each arm whose plasma HIV RNA is <200 copies/mL after 48 weeks. A virological threshold of 200 copies/mL was chosen because of recent work suggesting that this viral load threshold is sufficiently sensitive, while avoiding unreliable very low level viral load failures ('blips') that on subsequent measurement are not confirmed [41]. Contemporary studies of the use of a boosted-PI with background N(t)RTIs in ART naïve participants suggest that ~80% will return a plasma HIV RNA result <200 copies/mL after 48 weeks. In this study we would expect a similar response in a standard WHO recommended control arm of PI/r + 2-3N(t)RTIs. Non-inferiority will be defined as the lower 95% confidence limit of the difference in proportions with undetectable viral load lying above -12% (i.e. a non-inferiority margin of 12%).

Under the assumption that there is no difference between randomised treatment arms, to have 90% power to demonstrate non-inferiority in the ITT analysis using a 12% margin will require 248 participants per arm to be randomised (2-sided alpha=5%) making a total of 496 participants.

To ensure that the PP analysis also has 90% power to demonstrate non-inferiority, the sample size needs to be increased to compensate for participants who switch ART due to treatment toxicity over the course of the first 48 weeks of study. In this population this is estimated to be no more than 10% of randomised participants. Therefore, to ensure 90% power to demonstrate non-inferiority on the ITT and subsequently on the PP analyses, a total of 546 (i.e. 273 per arm) participants need to be randomised. We therefore aim to recruit a total of 550 participants to the study. The power of the study for the ITT analysis in this case is 92.9%.

11.2 Supplementary sample size assessments for exploratory analyses

Estimates of variability (standard deviations for changes from baseline) to detect differences in fasted lipid measures with an 80% power for pair-wise comparisons between arms I and II were obtained from the Gilead 903 and 934 studies [42,43]. Standard deviations were estimated based on mean changes and p-values quoted in the text.

1. Total cholesterol (mg/dl): 201 participants per arm has 80% power to detect a mean difference between study arms of 21 mg/dl (SD = 75 mg/dl)
2. LDL-cholesterol (mg/dl): 221 participants per arm has 80% power to detect a mean difference between study arms of 8 mg/dl (SD = 30 mg/dl)
3. HDL-cholesterol: 185 participants per arm has 80% power to detect a mean difference between study arms of 3.5 mg/dl (SD = 12 mg/dl)
4. Triglycerides mg/dl): 215 participants per arm has 80% power to detect a mean difference between study arms of 100 mg/dl (SD = 370 mg/dl)

11.3 Analysis plan

The criteria by which the experimental regimen of ritonavir-boosted lopinavir and raltegravir will be judged non-inferior to the control regimen will be if the lower limit of the 95% confidence interval for the difference in virological response between the two groups does not exceed -12%. Tests for non-inferiority will be primarily performed in analyses of the intention-to-treat (ITT) study population. If non-inferiority is demonstrated in the ITT population a supportive analysis of the PP population will be undertaken.

For secondary and exploratory efficacy endpoints the ITT population will be primary, although analyses of the PP population will also be performed. Safety endpoints will all be analysed according to ART received.

11.3.1 The intention to treat (ITT) population

The ITT population is defined as all participants who undergo randomisation and who receive at least one dose of study medication. Participants will be compared as randomised regardless of the treatment received.

During follow-up it is anticipated that a proportion of participants will experience various events which may result in different exposures to ART.

The following describes a framework for the analysis of the primary endpoint based on a particular handling of anticipated events:

I. in the event of the patient dying or becoming lost to follow-up the patient will be considered to have failed randomised treatment

II. in the event that ART is changed because of plasma HIV RNA ≥200 copies/mL the patient will be considered to have failed randomised treatment

III. ART changes for any other reason (other than II) do not constitute failure in the ITT population

IV. if the week 48 data are missing, a last observation carried forward (LOCF) imputation will be made for all participants, except for those who are missing week 36 data, who are dead, lost to follow-up, or have experienced virological failure (HIV RNA ≥200 copies/mL) that resulted in a change of ART.

This analysis corresponds to a comparison of the randomised treatment strategies, including all changes to ART regimens that occur subsequent to randomisation and will constitute the primary analysis.

A secondary analysis of this population but using a virological threshold of 50 and 400 copies/mL will also be undertaken

The following describes a framework for a secondary analysis of the ITT based on a different handling of the same anticipated events. This analysis will constitute a secondary endpoint of the study.

I. in the event of the patient dying or becoming lost to follow-up the patient will be considered to have failed randomised treatment

II. in the event that ART is changed because of plasma HIV RNA ≥200 copies/mL the patient will be considered to have failed randomised treatment

III. ART changes for any other reason (other than II), are considered failures

IV. if the week 48 data are missing, a last observation carried forward (LOCF) imputation will be made for all participants, except for those with week 36 data missing, who are dead, lost to follow-up, have experienced virological failure (HIV RNA ≥200 copies/mL) or have changed randomised ART for any other reason.

This analysis corresponds to a non-completer=failure (NC=F) analysis, making this secondary endpoint directly comparable with other trials.

11.3.2 The per protocol (PP) population

The PP population is defined as all participants included in the ITT population excluding those who changed randomly assigned ART for any reason other than plasma HIV RNA ≥200 copies/mL.

Analyses will be based on available data.

This approach corresponds to a comparison of the effectiveness of the two randomised ART regimens as if participants had adhered to their randomly allocated ART.

11.4 Schedule of analyses

There will be four formal analyses to be conducted within this protocol: two interim analyses of week 24 data (one when 125 participants each arm reach week 24; second when all participants reach week 24), a primary analysis at week 48 (all participants complete week 48) and a follow up confirmatory analysis at week 96 (all participants complete week 96).

The first interim analysis will occur after 125 participants in each arm (a total of 250 participants) have reached 24 weeks and will assess the rate of viral decline from baseline between the two groups. The aim of the analysis is to ensure that there is no evidence of a substantially different performance between regimens in virological suppression as judged by a difference between groups of >0.5 log10. Assuming that between-patient variability of time-weighted change in plasma HIV-1 RNA corresponds to a standard deviation of 1.0 log10, and adopting a significance level of 0.05, 125 participants per arm will confer >98% power to determine that the two-sided 95% confidence has an upper limit below 0.5 log10. A recommendation to stop the study at this analysis would only be made in the event that the experimental arm was considered inferior (i.e. if the control arm (arm I) is found to have provided a ≥ 0.5 log10 difference in virological suppression from baseline, using a ONE-sided significance level of 0.01). In the event that the experimental arm (arm II) appeared to be superior, the recommendation

would be to continue study until at least the second scheduled interim analysis in order to allow accumulation of sufficient data to make a reasonable judgement of the primary endpoint.

The second interim analysis will summarise study variables once all enrolled participants have completed 24 weeks of follow-up and will include all stated primary and key secondary measures of interest. The proportions of participants in each study arm at weeks 36 and 48 will also be compared at this time. The trial may be stopped if one arm is found to be inferior on the endpoint of percentage of participants with detectable plasma HIV RNA at week 24, using a two-sided significance level of 0.001. A recommendation of this sort could only be made if the finding for the week 24 data was consistent with the observations made at weeks 36 and 48 (in the sense that observations at all time points consistently suggest that one arm is inferior to the other).

Both interim analyses will be reviewed by an independent Data Safety Monitoring Board (DSMB) which will recommend to the Protocol Steering Committee that the study should continue unchanged or be amended in light of observed differences between treatment arms or aspects of study conduct that warrant modification (e.g. poor recruitment, safety concerns, and/or substantial losses to follow-up). Their review of treatment regimens will be coded as A and B. The DSMB, in agreement with Protocol Steering Committee, may require stopping rules to be implemented that require changes in the conduct of the study should concerns surrounding the safety of participants arise from review of the week 24 results.

In addition, the DSMB will be provided with information relating to the conduct and management of the study. Un-blinded datasets will not be made available for review outside of the DSMB and relevant project statistician.

The primary analysis will summarise study variables when the last patient randomised has completed 48 weeks of follow-up. This analysis will be reviewed firstly by the Protocol Steering Committee and then presented publicly with treatment arms identified. Since this constitutes the final analysis of week 48 data (the primary analysis point), decisions regarding the future conduct of the trial will be made based on statistical significance at the conventional $p<0.05$ level.

It is explicitly recognised that publication of the week 48 data may affect conduct of the trial between weeks 48 and 96, and so may affect final results between those time points. The 96-week analysis will provide longer term durability and safety data.

11.5 Demographics and baseline characteristics

Demographic and baseline characteristics will be summarised by randomised treatment group. There will be no formal comparisons of randomised treatments for demographic and baseline characteristics (i.e. no p-values), as any imbalance will by definition have occurred by chance.

11.6 Efficacy analyses

Treatment estimates and 95% confidence intervals will be calculated and used to assess primary and secondary efficacy endpoints. If non-inferiority is established for the primary comparison (ITT and secondary PP analyses) further analyses to assess for superiority will be undertaken.

Binary endpoints will be analysed using chi-square tests or logistic regression. Continuous endpoints will be analysed using ANOVA methods or non-parametric equivalents. Time to event endpoints will be analysed using survival analysis methods.

For all endpoints and analysis populations, the primary treatment comparisons will be simple, unadjusted, two-group comparisons. If there are important imbalances in baseline characteristics, then adjusted analyses will also be performed and presented in addition to unadjusted analyses.

11.7 Subgroup analyses

There will be four pre-specified stratified analyses performed. Outcomes of the primary and key secondary efficacy variables will be compared in strata defined by baseline plasma HIV-1 RNA viral load < or ≥100,000 copies/mL. There will also be a pre-specified analysis according to whether sites used HIV genotyping at baseline for construction of the N(t)RTI backbone in the control arm and an analysis of outcomes according to World Bank defined income criteria (high, middle and low) [43]. It is accepted that the reduced power of these comparisons will make establishing non-inferiority within each strata unlikely (i.e. increase the probability of making a Type II error). The consistency of treatment comparisons across strata will be assessed using formal statistical tests for interaction between treatment group and strata.

11.8 Safety analyses

The proportion of participants with all grade adverse events and grade 3/4 adverse events will be summarised by randomised treatment group, by severity and by relation to study drug for all subjects treated with study drug. Serious adverse events will be summarised for all enrolled subjects. The incidence rate of adverse events will also be calculated. The analysis of safety variables will be done according to a per protocol (see 11.3.2) approach.

12 DATA COLLECTION, SOURCE DOCUMENTS AND RECORD RETENTION

12.1 Data collection and source documents

An Investigator is required to prepare and maintain adequate and accurate case histories designed to record all observations and other data pertinent to the investigation on each individual treated with the investigational

product or entered as a control in the investigation. Data reported on the CRF that are derived from source documents must be consistent with the source documents or the discrepancies must be explained.

The CRF for this study will be an Electronic Data Capture (EDC) system using a web-enabled password protected platform.

The confidentiality of records that could identify subjects must be protected, respecting the privacy and confidentiality rules in accordance with the applicable regulatory requirement(s).

Corrections to CRFs will only be possible by study personnel with sufficient authorisation to make changes. All changes will record time, date, computer ID and the name of the authorised person's access code.

12.2 Records retention

The Investigator must retain investigational product disposition records, copies of CRFs (or electronic files), and source documents for the maximum period required by applicable regulations and guidelines, or Institution procedures, or for the period specified by the NCHECR, whichever is longer. The Investigator must contact the Sponsor prior to destroying any records associated with the study.

If the Investigator withdraws from the study (e.g. relocation, retirement), the records shall be transferred to a mutually agreed upon designee (i.e. another Investigator, IRB). Notice of such transfer will be given in writing to the NCHECR.

12.3 Monitoring for protocol compliance

Representatives of the Sponsor must be allowed to visit all study site locations periodically to assess the data, quality and study integrity. On site they will review study records and directly compare them with source documents and discuss the conduct of the study with the Investigator, and verify that the facilities remain acceptable. Such visits will be scheduled in advance and with sufficient warning to allow arrangements of diaries and personnel as appropriate.

There will be a total of 5 visits throughout the 2 year study. The first will be an initiation visit prior to study commencement. The 4 monitoring visits will be conducted firstly within a couple of months of the first participant being randomised, then when all participants have completed week 24, 48 and 96.

In addition, the study may be evaluated by the regulatory authorities. Audit reports will be kept confidential.

THE INVESTIGATOR MUST NOTIFY THE SPONSOR PROMPTLY OF ANY INSPECTIONS SCHEDULED BY REGULATORY AUTHORITIES, AND PROMPTLY FORWARD COPIES OF INSPECTION REPORTS TO NCHECR.

12.4 Auditing

The study may be subject to audit by properly authorised individuals but such information will be treated as strictly confidential and will in no circumstances be made publicly available. Under such circumstances, the investigator must agree to allow access to study documents and relevant hospital/clinic records.

13 ETHICS COMMITTEE/REGULATORY APPROVAL AND INFORMED CONSENT

13.1 Ethics

This study will be conducted in accordance with the ethical principles that have their origin in the Declaration of Helsinki and will be consistent with Good Clinical Practice (GCP) and applicable regulatory requirements.

The study will be conducted in compliance with the protocol. The protocol, any Amendments and the Participant Informed Consent (PIC) will receive Institutional Review Board (IRB)/Human Research Ethics Committee (HREC) approval prior to initiation of the study.

Freely given written informed consent must be obtained from every research subject or his or her legally acceptable representative prior to clinical trial participation, including informed consent for any screening procedures conducted to establish eligibility for the trial.

For further details on informed consent, see Section 13.5

The rights, safety and wellbeing of the trial subjects are the most important considerations and should prevail over interests of science and society.

Study personnel involved in conducting this trial will be qualified by education, training, and experience to perform their respective task(s).

This trial will not use the services of study personnel where sanctions have been invoked or where there has been scientific misconduct or fraud (e.g. loss of medical licensure, debarment).

Systems with procedures that assure the quality of every aspect of the study will be implemented.

13.2 Institutional review board/independent ethics committee (IRB/IEC)

Before study initiation, the Investigator must have written and dated approval/favourable opinion from the IRB/IEC for the protocol, consent form, subject recruitment materials/process (e.g. advertisements) and any other written information to be provided to subjects. The Investigator should also provide the IRB/IEC with a copy of the product labelling and information to be provided to subjects and any updates.

The Investigator should provide the IRB/IEC with reports, updates, and other information (e.g., Safety Updates, Amendments, and Administrative Letters) according to regulatory requirements or Institution procedures.

13.3 Compliance with the protocol and protocol revisions

The study shall be conducted as described in this approved protocol. The Investigator should not implement any deviation or change to the protocol without prior review and documented approval/favourable opinion from the Institutional Review Boards/Human Research Ethics Committees (IRB/HREC) of an Amendment, except where necessary to eliminate an immediate hazard(s) to study subjects. Any significant deviation must be documented in the CRF.

If a deviation or change to a protocol is implemented to eliminate an immediate hazard(s) prior to obtaining IRB/IEC approval/favourable opinion, as soon as possible the deviation or change will be submitted to:

- IRB/IEC for review and approval/favourable opinion;
- The Sponsor
- Regulatory Authority(ies), if required by local regulations.

Documentation of approval signed by the chairperson or designee of the IRB(s)/HREC(s) must be sent to NCHECR along with other required documentation as outlined in the Manual of Operations.

If the revision is an Administrative Letter, Investigators must inform their IRB(s)/HREC(s).

If an Amendment substantially alters the study design or increases the potential risk to the subject: (1) the consent form must be revised and submitted to the IRB(s)/IEC(s) for review and approval/favourable opinion; (2) the revised form must be used to obtain consent from subjects currently enrolled in the study if they are affected by the Amendment; and (3) the new form must be used to obtain consent from new subjects prior to enrolment.

13.4 Informed consent

Investigators must ensure that subjects or their legally acceptable representative are clearly and fully informed about the purpose, potential risks and other critical issues regarding clinical trials in which they volunteer to participate.

13.5 Informed consent procedures

Preparation of the consent form is the responsibility of the Investigator and must include all elements required by ICH GCP and applicable regulatory requirements, and must adhere to GCP and to the ethical principles that have their origin in the Declaration of Helsinki. The consent form must also include a statement that study

sponsors and regulatory authorities will have direct access to subject records. Prior to the beginning of the study, the Investigator must have the IRB/IEC's written approval/favourable opinion of the written informed consent form and any other information to be provided to the subjects.

The Investigator must provide the subject or legally acceptable representative with a copy of the consent form and written information about the study in the language in which the subject is most proficient. The language must be non-technical and easily understood. The Investigator should allow time necessary for subject or subject's legally acceptable representative to inquire about the details of the study, then informed consent must be personally signed and dated by the subject or the subject's legally acceptable representative and by the person who conducted the informed consent discussion. The subject or legally acceptable representative should receive a copy of the signed informed consent and any other written information provided to study subjects prior to subject's participation in the trial.

13.5.1 Other circumstances

Imprisonment or the compulsory detention for treatment of either a psychiatric or physical (e.g. infectious disease) illness precludes enrolment in clinical trials.

When a subject may be in a dependent relationship with the investigator, a well-informed physician who is not engaged in the clinical trial and is completely independent of the relationship between the subject and investigator should obtain the subject's informed consent.

13.5.2 Update of informed consent

The informed consent and any other information provided to subjects or the subject's legally acceptable representative, should be revised whenever important new information becomes available that is relevant to the subject's consent, and should receive IRB/IEC approval/favourable opinion prior to use. The Investigator, or a person designated by the Investigator should fully inform the subject or the subject's legally acceptable representative of all pertinent aspects of the study and of any new information relevant to the subject's willingness to continue participation in the study. This communication should be documented.

During a subject's participation in the trial, any updates to the consent form and any updates to the written information will be provided to the subject.

14 CONFIDENTIALITY OF DATA

14.1 Confidentiality of participant records

By signing of the protocol, the investigator agrees that the sponsor, ethics committee or regulatory authorities may consult and/or copy study documents to verify information in the case record form. By signing of the consent form the participant agrees to this process.

Participant confidentiality will be maintained at all times and no documents containing the participant's name or other identifying information will be collected by the sponsor. It may be necessary for the sponsor's representatives, the ethics committee and regulatory authority representatives to have direct access to the subject/participant's medical records. If study documents need to be photocopied during the process of verifying case record form data, the subject/participant will be identified by a unique code only; full names and other identifying information will be masked.

14.2 Confidentiality of study data

By signing this protocol, the investigator affirms to the sponsor that information provided to the investigator by the sponsor will be maintained in confidence and will be divulged only as necessary to the ethics committee and institution employees directly involved in the study. Both ethics committee members and employees must also understand the confidentiality requirements for any information divulged to them. The data generated by this study will be considered confidential by the investigator, except to the extent that it is included in a publication as agreed in the publication policy of this protocol.

15 FINANCING AND INSURANCE

Investigators will be paid according to the separate financial agreement document that must be signed and dated prior to study commencement.

It is the investigators responsibility to provide details of the study budget to the ethics committee, as required by each individual ethics committee. Unless the clinical site comes under the St Vincent's Hospital ethics application, in which case NCHECR will do this on behalf of the investigator.

Indemnification for site personnel, investigators and institutions will be provided as required in keeping with the provisions as determined by the Medicines Australia guidelines through the University of New South Wales. Details of this provision will be documented in separate agreements.

No fault compensation is available to study participants who experience injury as a result of their participation in this study. Details of the method of compensation will be provided in separate agreements between institutions.

16 QUALITY CONTROL AND QUALITY ASSURANCE

By signing of this protocol, the sponsor agrees to be responsible for implementing and maintaining quality control and quality assurance systems with written standard operating procedures. These procedures will ensure that trials are conducted and data are generated, documented and reported in compliance with the protocol, Good Clinical Practice standards and all applicable local laws and regulations relating to the conduct of a clinical trial.

17 PUBLICATION POLICY

In the interests of collegiality and recognising that completion of this trial will have resulted from the contribution of many people the masthead authorship for the manuscript will be "The SECOND-LINE Study Group". The protocol steering committee (PSC) will constitute the writing committee for the primary manuscript. In addition, one person from each investigational site will be listed in a separate appendix as being part of the SECOND-LINE Study Group. The PSC will determine if there is a need for other appendices in which to identify others who have contributed in a significant way to the design AND conduct AND reporting of resultant trial data. If the journal will not accept group authorship the writing committee will be listed as authors and be completed with the phrase 'on behalf of the SECOND-LINE Study Group'.

Additional manuscripts that are expected to report on the findings of any subsequent sub-studies should have named investigators and be completed with the phrase 'on behalf of the SECOND-LINE Study Group'. In these circumstances an appendix should contain the names of the PSC.

All proposed manuscripts should be submitted to the PSC 45 days before they are to be submitted to a journal for peer review.

Conference presentations should identify an authorship group consistent with those who have contributed to the data to be reported. All proposed conference presentations should be submitted to the PSC at least 20 days before submission of an abstract.

References

[1] Grinsztejn B, Nguyen BY, Katlama C, et al. Safety and efficacy of the HIV-1 integrase inhibitor raltegravir (MK-0518) in treatment-experienced patients with multidrug-resistant virus: a phase II randomized controlled trial. *Lancet* **2007**;369:1261-9.

[2] Markowitz M, Nguyen BY, Gotuzzo E, et al. Rapid and durable antiretroviral effect of the HIV-1 integrase inhibitor raltegravir as part of combination therapy in treatment naïve patients with HIV-1 coinfection. *J Acquir Immune Defic Syndr* **2007**; 46;125-133.

[3] Murray JM, Emery S, Kelleher A, et al. The integrase inhibitor raltegravir alters viral decay kinetics of HIV, significantly reducing the second phase. *AIDS* **2007**; 21:2315-2321.

[4] Department of Health and Human Services (USA). Guidelines for the use of antiretroviral agents in HIV-1 infected adults and adolescents. November 03, **2008**. Available at: http://aidsinfo.nih.gov/contentfiles/AdultandAdolescentGL.pdf

[5] World Health Organization. Antiretroviral Therapy for HIV Infection in Adults and Adolescents in Resource-Limited Settings. Towards Universal Access. Geneva, Switzerland: WHO, **2006** revision. Available at: http://www.who.int/hiv/pub/guidelines/art/en/index.html

[6] Humphreys EH, Hernandez LB, Rutherford GW. Antiretroviral regimens for patients with HIV who fail first-line antiretroviral therapy (Review). *Cochrane Database of Systematic Reviews* **2007**, Issue 4. Art. No.: CD006517. DOI: 10.1002/14651858.CD006517.pub2.

[7] Rajasekaran S, Jayaseelan L, Vijila S, Gomathi C, Raja K. Predictors of failure of first-line antiretroviral therapy in HIV-infected adults: Indian experience. *AIDS* **2007**;21 (suppl 4): S47-S53.

[8] Cozzi-Lepri A, Phillips AN, Ruiz L, et al. Evolution of drug resistance in HIV-infected patients remaining on a virologically failing combination antiretroviral therapy regimen. *AIDS* **2007**;21:721-32.

[9] World Health Organization. Significant growth in access to HIV treatment in 2006. Available at: HHUU http:// www.int/mediacentre/news/releases/2007/pr16/en/index.htmlUU

[10] Galarraga O, O'Brien ME, Gutierrez JP, et al Forecast of demand for antiretroviral drugs in low- and middle-income countries: 2007-2008 *AIDS* **2007**;21 (suppl 4): S97-S103.

[11] Tam LW, Hogg RS, Yip B, Montaner JS, Harrigan PR, Brumme CJ. Performance of a World Health Organization first-line regimen (stavudine/lamivudine/nevirapine) in antiretroviral-naive individuals in a Western setting. *HIV Med* **2007**;8:267-70.

[12] Zhou J, Paton NI, Ditangco R, et al. Experience with the use of a first-line regimen of stavudine, lamivudine and nevirapine in patients in the TREAT Asia HIV Observational Database. *HIV Med* **2007**;8:8-16.

[13] Sungkanuparph S, Manosuthi W, Kiertiburanakul S, Piyavong B, Chumpathat N, Chantratita W. Options for a second-line antiretroviral regimen for HIV type 1-infected patients whose initial regimen of a fixed-dose combination of stavudine, lamivudine, and nevirapine fails. *Clin Infect Dis* **2007**;44:447-52.

[14] Sungkanuparph S, Manosuthi W, Kiertiburanakul S, Chantratitra W. Tenofovir resistance among HIV-infected patients failing a fixed-dose combination of stavudine, lamivudine, and nevirapine in a resource-limited setting. *AIDS Pat Care STDs* **2007**; 21:711-713.

[15] Hosseinipour M et al. Resistance profile of patients failing first line ART in Malawi when using clinical and immunologic monitoring. *Program and Abstracts of the Seventeenth International AIDS Conference*, Mexico DF, Mexico, 03-08 August **2008**. Abstract number TUAB0105.

[16] Phillips AN, Pillay D, Miners AH, et al. Outcomes from monitoring of patients on antiretroviral therapy in resource-limited settings with viral load, CD4 cell count, or clinical observation alone: a computer simulation model. *Lancet* **2008**;371:1443-51.

[17] Hogg RS, Bangsberg DR, Lima VD, et al. Emergence of drug resistance is associated with an increased risk of death among patients first starting HAART. *PLoS Med* **2006**;3:e356.

[18] Deeks, S. When to switch? First line failure and its implications. Program and abstracts of the 4th IAS Conference on HIV Pathogenesis, Treatment and Prevention, 22-25 July, **2007**, Sydney, Australia. Abstract number TUBS103.

[19] Walmsley S, Bernstein B, King M, et al. Lopinavir-ritonavir versus nelfinavir for the initial treatment of HIV infection. *N Eng J Med* **2002** ;346:2039-46.]

[20] Dragsted UB, Gerstoft J, Youle M, et al. A randomized trial to evaluate lopinavir/ritonavir versus saquinavir/ritonavir in HIV-1-infected patients: the MaxCmin2 trial. *Antivir Ther* **2005**;10:735-43.

[21] Eron J, Jr., Yeni P, Gathe J, Jr., et al The KLEAN study of fosamprenavir-ritonavir versus lopinavir-ritonavir, each in combination with abacavir-lamivudine, for initial treatment of HIV infection over 48 weeks: a randomized non-inferiority trial. *Lancet* **2006**;368:476-82.

[22] Madruga JV, Berger D, McMurchie M, et al. Efficacy and safety of darunavir-ritonavir compared with that of lopinavir-ritonavir at 48 weeks in treatment-experienced, HIV-infected patients in TITAN: a randomized controlled phase III trial. *Lancet* **2007**;370:49-58.

[23] Gathe J, da Silva BA, Cohen DE, et al. A once-daily lopinavir/ritonavir-based regimen is noninferior to twice daily dosing and results in similar safety and tolerability in antiretroviral-naïve subjects through 48-weeks. J Acquir Immune Defic Syndr 2009;50:474-481.

[24] Gilks CF, Crowley S, Ekpini R, et al. The WHO public-health approach to antiretroviral treatment against HIV in resource-limited settings. Lancet 2006; 368:505-10.

[25] Straszewski S, Morales-Ramirez J, et al.Efavirenz plus zidovudine and lamivudine, efavirenz plus indinavir, and indinavir plus zidovudine and lamivudine in the treatment of HIV-1 infection in adults. Study 006 Team.. New Engl J Med 1999; 341:1865-73.

[26] Riddler SA, Haubrich R, Di Rienzo AG, et al. Class-sparing regimens for initial treatment of HIV-1 infection. N Engl J Med. 2008;358:2095-106.

[27] Boyd MA, Siangphoe U, Ruxrungtham K, et al. Indinavir/ritonavir 800/100 mg bid and efavirenz 600 mg qd in patients failing treatment with combination nucleoside reverse transcriptase inhibitors: 96-week outcomes of HIV-NAT 009. *HIV Med* **2005**;6:410-20.

[28] Avihingsanon A, Boyd MA, Pornprasit P, et al. Long term durability of boosted indinavir and efavirenz in patients with combination nucleoside analogue failure in a resource constrained setting: 4- year follow-up of HIV-NAT 009. *Program and abstracts of the Sixteenth International AIDS Conference, Toronto*, Canada, 13-18 August, **2006**. Abstract number THPE0126.

[29] Pinola M, Carosi G, Di Perri G, et al. LPV/r-based 2-drug HAART vs LPV/r-based 3-drug HAART: Comparable virological efficacy and tolerability in HIV-1-infected naive subjects (Kalead1 study) Ð 48-Week results. *Program and Abstracts of the Fourth IAS Conference on HIV Pathogenesis, Treatment and Prevention*, Sydney, Australia, 22-25 July **2007**Abstract number WEPEB035.

[30] Lazzarin A, Clotet B, Cooper D, et al. Efficacy of enfuvirtide in patients infected with drug-resistant HIV-1 in Europe and Australia. *N Eng J Med* **2003**;348:2186-95.

[31] Hicks CB, Cahn P, Cooper DA, et al. Durable efficacy of tipranavir-ritonavir in combination with an optimised background regimen of antiretroviral drugs for treatment-experienced HIV-1-infected patients at 48 weeks in the Randomized Evaluation of Strategic Intervention in multi-drug reSistant patients with Tipranavir (RESIST) studies: an analysis of combined data from two randomised open-label trials.. *Lancet* **2006**;368:466-75.

[32] Clotet B, Bellos N, Molina JM, et al. Efficacy and safety of darunavir-ritonavir at week 48 in treatment-experienced patients with HIV-1 infection in POWER 1 and 2: a pooled subgroup analysis of data from two randomized trials. *Lancet* **2007**;369:1169-78.

[33] Cooper DA, Steigbigel RT, Gatell JM, et al. Subgroup and resistance analyses of raltegravir for resistant HIV-1 infection. *N Engl J Med*. **2008** 359:355-65.

[34] Fätkenheuer G, Nelson M, Lazzarin A, et al. Subgroup analyses of maraviroc in previously treated R5 HIV-1 infection. *N Engl J Med* **2008**;359:1442-55.

[35] World Health Organization. Prioritizing Second-Line Antiretroviral Drugs for Adults and Adolescents: a Public Health Approach. Report of a WHO Working Group Meeting. Available at: http://www.who.int/hiv/pub/meetingreports/second_line_art_report_2008.pdf

[36] Negredo E, Molto J, Burger D et al. Unexpected CD4 cell count decline in patients receiving didanosine and tenofovir-based regimens despite undetectable viral load. *AIDS* 2004;**18**:459–63

[37] Maitland D, Moyle G, Hand J et al. Early virological failure in HIV-1 infected subjects on didanosine/tenofovir/efavirenz: 12-week results from a randomized trial. *AIDS* 2005;19:1183–8

[38] Martínez E, Milinkovic A, de Lazzari E, et al. Pancreatic toxic effects associated with co-administration of didanosine and tenofovir in HIV-infected adults. Lancet 2004;364:65-7.

[39] Eron J, Andrade J, Zajdenverg R, et al. Switching from stable lopinavir/ritonavir-based combination ART resulted in a superior lipid profile at week 12 but did not demonstrate non-inferior virological efficacy at week 24. Program and abstracts of the 16th Conference on Retroviruses and Opportunistic Infections, February 8-11, 2009, Montreal, Canada; abstract 70aLB.

[40] French MA, Price P, Stone SF. Immune restoration disease after antiretroviral therapy. *AIDS* **2004** Aug 20;18(12):1615-27

[41] Ribaudo H, Lennox J, Currier J, et al., Virological Failure Endpoint Definition in Clinical Trials Is using HIV-1 RNA Threshold <200 copies/mL Better than <50 Copies/mL? An Analysis of ACTG Studies. Program and abstracts of the 16th Conference on Retroviruses and Opportunistic Infections, February 8-11, 2009, Montreal, Canada, abstract 580.

[42] Gallant JE, Straszewski S, Pozniak AL, et al Efficacy and safety of tenofovir DF vs stavudine in combination therapy in antiretroviral-naive patients: a 3-year randomized trial. *JAMA* **2004**;292:191-201.

[43] Gallant JE, De Jesus E, Arribas JR, et al. Tenofovir DF, emtricitabine, and efavirenz vs. zidovudine, lamivudine, and efavirenz for HIV. *N Engl J Med* **2006**;354:251-60.

[44] The World Bank. Data and Statistics. Available at: http://web.worldbank.org

Appendix 1: Definitions and Criteria For HIV Disease and AIDS Events

The following list encompass the CDC's 1993 surveillance case definition of AIDS (without the CD4+ criterion), with the addition of several diagnoses increasingly felt to be associated with severe immunosuppression in participants infected with HIV.

Modified CDC Category C 1993 Definition
- Candidiasis of bronchi, trachea or lungs
- Candidiasis, esophageal
- Cervical cancer, invasive
- Coccidioidomycosis, disseminated or extrapulmonary
- Cryptococcosis, extrapulmonary
- Cryptosporidiosis, chronic intestinal (> 1 month's duration)
- CMV disease (other than liver, spleen, or nodes)
- CMV retinitis (with loss of vision)
- Encephalopathy, HIV-related (including AIDS Dementia Complex)
- *Herpes simplex*, chronic ulcers (> 1 month's duration); or bronchitis, pneumonitis or esophagitis
- Histoplasmosis, disseminated or extrapulmonary
- Isosporiasis, chronic intestinal (> 1 month's duration)
- Kaposi's sarcoma (mucocutaneous or visceral)
- Lymphoma, Burkitt's (or equivalent term)
- Lymphoma, primary, of brain
- *Mycobacterium avium* complex or *M. kansasii*, disseminated or extrapulmonary
- *M. tuberculosis*, any site (pulmonary or extrapulmonary)
- *Mycobacterium*, other species or unidentified species, disseminated or extrapulmonary
- *Pneumocystis carinii* pneumonia (*pneumocystic jiroveci*)
- Pneumonia, recurrent bacterial (2 documented episodes within 1 year of each other following randomization)
- Progressive multifocal leukoencephalopathy
- *Salmonella* septicemia, recurrent (2 documented episodes within 1 year of each other following randomization)
- Toxoplasmosis of brain
- Wasting syndrome due to HIV

Additions to CDC Definition
- Aspergillosis, invasive
- Bartonellosis
- Chagas disease (American trypanosomiasis) of the CNS
- *Herpes zoster*, multi-dermatomal (≥10 lesions in a non-contiguous site)
- Leishmaniasis, visceral (kala-azar)
- Lymphoma, Hodgkin's
- Lymphoma, non-Hodgkin's, all cell types
- Microsporidiosis (> 1 month's duration)
- Nocardiosis
- *Penicillium marneffii*, disseminated
- *Pneumocystis carinii*, extrapulmonary
- *Rhodococcus equi* disease

	CONFIRMED	PROBABLE
**CONSTITUTIONAL DISEASE**		
HIV wasting syndrome	None	A plus B plus C: (A) unexplained, involuntary weight loss >10% from baseline, (B) persistent diarrhoea with >2 liquid stools/d for >1 month or weakness for >1 month or fever for >1 month, (C) tests for alternate causes of weight loss, such as cancer, TB, MAC, cryptosporidiosis or other specific causes of weight loss, if obtained, should be negative
**INFECTIONS**		
Aspergillosis, invasive pulmonary	A plus B plus C: (A) CXR abnormality compatible with aspergillosis, (B) invasive mycelia consistent with Aspergillus on lung biopsy, (C) positive culture from lung biopsy or sputum collected by any method	A plus B: (A) CXR abnormality compatible with aspergillosis, (B) invasive mycelia consistent with Aspergillus on lung biopsy or positive culture of lung tissue or positive culture of sputum collected by any method
Aspergillosis, other invasive	A plus B plus C: (A) compatible clinical course, (B) invasive mycelia consistent with Aspergillus on tissue biopsy or clinical evidence of infection, (C) positive culture from the affected tissue	A plus B: (A) clinical evidence of invasive infection, (B) invasive mycelia consistent with Aspergillus on tissue biopsy or positive culture at a normally sterile site (e.g., blood) apart from the involved tissue
Bartonellosis	A plus B: (A) Clinical or histologic evidence of bacillary angiomatosis or bacillary peliosis, (B) a positive culture or PCR for *B. quintana* or *B. henselae*	A plus B: (A) Clinical evidence of bacillary angiomatosis or bacillary peliosis, (B) positive silver stain for bacilli from a skin lesion or an affected organ
Candidiasis of bronchi, trachea or lungs	Macroscopic appearance at bronchoscopy or autopsy plus microscopic evidence of yeasts or pseudo hyphae	None
Candidiasis, esophageal	A plus B: (A) Macroscopic appearance at esophagoscopy or autopsy, (B) microscopic evidence of yeasts or pseudo hyphae	A plus B plus C: (A) Recent onset of retrosternal pain or difficulty on swallowing, (B) a clinical diagnosis of oral candidiasis plus microscopic evidence of yeasts or pseudo hyphae from oropharyngeal mucosa, (C) clinical response to treatment

	**CONFIRMED**	**PROBABLE**
**INFECTIONS (CONTINUED)**		
Chagas disease (American trypanosomiasis) of the CNS	Histologic evidence obtained by brain tissue biopsy or autopsy	A plus B plus C plus D: (A) Focal, typically hemispheral neurological dysfunction with onset over several days or weeks; (B) enhancing focal lesion(s) with mass effect, and surrounding edema and contrast enhancement, typically located in grey matter; (C) serum antibodies to *T. cruzi*, (D) response to standard therapy with documented clinical or radiographic improvement (if radiography was done, it must be improved), or peripheral blood smear or CSF smear positive for *T. cruzi*
Coccidioidomycosis, disseminated or extrapulmonary	From tissue other than lung or hilum, A or B or C: (A) Microscopic demonstration of spherules, (B) positive culture, (C) antigen detection	None
Cryptococcosis, meningitis or extrapulmonary	From tissue other than lung or hilum, A or B or C or D: (A) microscopic demonstration of narrow based budding yeast, (B) for meningitis, if done, a positive CSF India ink test, (C) positive culture, (D) antigen detention	None
Cryptosporidiosis	Diarrhea for >1 month and positive microscopy	None
CMV retinitis	Autopsy demonstration	Typical appearance on fundoscopy of discrete patches of retinal whitening, spreading along blood vessels, associated with vasculitis, hemorrhage and necrosis, confirmed by ophthalmologist
CMV radiculomyelitis	Autopsy demonstration	A plus B plus C plus D plus E plus F: (A) Loss of sensation, leg weakness, or decreased reflexes, (B) presentation over 3 days to 3 weeks, (C) CT, MRI or myelogram must be done and all imaging studies must not show a mass lesion, (D) CSF shows >10 WBC with >50% polymorphs, (E) CSF shows no other pathogen, (F) persistence of symptoms in the absence of CMV treatment, or elevated quantitative CMV in the CSF by PCR

	CONFIRMED	PROBABLE
**INFECTIONS (CONTINUED)**		
CMV meningoencephalitis	Autopsy or brain biopsy demonstration	A plus B: (A) Rapid <4 weeks syndrome with progressive delirium, cognitive impairment and fever, (B) CT/MRI demonstration of periventricular abnormalities or elevated quantitative CMV DNA in the CSF by PCR
CMV, other disease	A plus B plus C plus D: (A) compatible illness, (B) histologic demonstration of inclusion bodies from affected tissue, (C) if done, detectible CMV antibodies, (D) if done, detectible CMV DNA or CMV antigen in blood	A plus B plus C plus D: (A) Compatible illness, (B) moderate to markedly high CMV antigen or CMV DNA in blood, (C) response to therapy, (D) if done, detectible CMV antibodies
HSV mucocutaneous ulceration	A plus B: (A) Ulceration for >1 month, (B) histology or culture or detection of antigen from affected tissue	A plus B: (A) Typical HSV ulceration for >1 month, (B) response to an antiviral active against HZV unless resistance is demonstrated
HSV, bronchitis, pneumonitis, esophagitis or other visceral disease	A plus B: (A) Compatible symptoms, (B) histology or culture or detection of antigen from affected tissue	None
HZV, disseminated	A plus B: (A) multiple ulcerated lesions affecting at least 2 non-contiguous dermatomes, or with genralised cutaneous dissemination: or HZV involvement of the lung, liver, brain, or other internal organs (B) positive culture, PCR, or antigen asssay from from affected tissue	A plus B: (A) multiple typical ulcerated lesions affecting at least 2 non-contiguous dermatomes, or with generalised cutaneous dissemination (B) response to an antiviral active against HZV unless resistance is demonstrated
Histoplasmosis, disseminated or extrapulmonary	A plus B: (A) Compatible symptoms, (B) histology or culture or elevated blood or urine antigen levels	None
Isosporiasis	Diarrhea for >1 month, plus microscopic identification of *Isospora belli*	None
Leishmaniasis, visceral	Compatible symptoms, plus microscopic identification of Leishmania	None
Microsporidiosis	Diarrhea for >1 month plus Microscopic identification of Microsporidia	None
MAC and other mycobacterial disseminated disease	A plus B: (A) Fever, fatigue, anemia or diarrhea, (B) positive culture from blood, body fluids or tissue other than pulmonary, hilar or stool	A plus B plus C: (A) Fever, fatigue, anemia or diarrhea, (B) AFB or positive direct MAC PCR in blood, body fluids or tissue other than pulmonary, hilar or stool (C) no concurrent non-pulmonary TB

	**CONFIRMED**	**PROBABLE**
**INFECTIONS (CONTINUED)**		
M. tuberculosis disease, pulmonary	A plus B: (A) Compatible symptoms of fever, dyspnea, cough, weight loss or fatigue, (B) culture or PCR from sputum or bronchial lavage or lung tissue	A plus B plus C plus D: (A) Symptoms of fever, dyspnea, cough, weight loss or fatigue, (B) abnormal chest X-ray, (C) AFBs seen in sputum or lavage or lung tissue but not grown in culture, (D) responds to treatment
M. tuberculosis disease, extrapulmonary	A plus B: (A) Compatible symptoms, (B) culture or PCR from blood or affected tissue	A plus B plus C: (A) Compatible symptoms, (B) AFBs seen from affected tissue or blood (C) concurrent diagnosis of pulmonary TB or responds to treatment
Nocardiosis	Clinical evidence of invasive infection plus a positive culture from the affected tissue or blood	Clinical evidence of invasive infection plus microscopic evidence of bronchial weakly acid fast organisms from the affected tissue
Penicillium marneffei, disseminated	Culture from a non-pulmonary site	Known presence in a *P. marneffei* endemic area plus characteristic skin lesions plus response to antifungal therapy for penicillosis
PCP	A plus B: (A) compatible clinical syndrome, (B) microscopic or histoloical demonstration of *P. carinii* cysts in a pulmonary specimen	A plus B plus C plus D plus E: (A) dyspnea or cough, or fever progressive over >1 week, (B) diffuse chest x-ray abnormality or, if on inhalational pentamidine, diffuse upper lung field abnormality, (C) evidence of hypoxia, (D) not suggestive of bacterial pneumonia (i.e., not purulent sputum or hemoptysis, no bacterial pathogen identified in blood or bronchial wash), (E) response to PCP treatment
Pneumocystis jirovecii, extrapulmonary	Compatible symptoms, plus microscopy	None

	CONFIRMED	PROBABLE
**INFECTIONS (CONTINUED)**		
Pneumonia, recurrent bacterial excludes, (a) post-obstructive pneumonias, including in those with intrapulmonary/bronchial lesions, (b) aspiration pneumonia, including in those with decreased consciousness levels, (c) nosocomial ventilator-associated pneumonias	Both pneumonia episodes must occur after enrolment and satisfy criteria (A) plus (B) plus (C). The recurrent pneumonia must also satisfy criteria (D) plus (E): (A) Signs and symptoms suggestive of bacterial pneumonia, (B) focal CXR abnormality compatible with bacterial pneumonia, (C) identification of a bacterial pathogen by positive blood culture, positive bronchoscopic sputum culture, detection of Legionella or pneumococcal antigen in urine or blood or diagnostic serologic findings, (D) the second pneumonia had onset of symptoms <365 days after the first episode, (E) there is strong evidence that the first episode was cured such as an intervening clear CXR or absence of symptoms after >1 month off antibacterials effective against pathogens commonly producing pneumonia	Both pneumonia episodes must occur after enrollment and satisfy criteria (A) plus (B) plus (C). The recurrent pneumonia must also satisfy criteria (D) plus (E): (A) Signs and symptoms suggestive of bacterial pneumonia, (B) focal CXR abnormality compatible with bacterial pneumonia, (C) diagnosed by a doctor, physicians' assistant or nurse practitioner, (D) the second pneumonia had onset of symptoms <365 days after the first episode, (E) there is strong evidence that the first episode was cured such as an intervening clear CXR or absence of symptoms after >1 month off antibacterials effective against pathogens commonly producing pneumonia
PML (progressive multifocal leukoencephalopathy)	A or B: (A) positive histology, (B) compatible clinical and radiologic course and positive CSF PCR for JK virus	A plus B plus C: (A) Consistent symptoms, (B) brain image consistent with PML, (C) no response to toxo treatment or toxoplasma seronegative
Rhodococcus equi disease	Clinical evidence of invasive infection plus microbiologic identification of the organism in the affected tissue or blood	None
Salmonella septicemia, recurrent	Both episodes must occur after enrollment and met criterion (A). The second episode must meet criteria (B) and C: (A) Positive blood or tissue culture, (B) the second septicemia had onset of symptoms <365 days after the first episode, (C) the second septicemia must be due to a different Salmonella serotype or there must be strong evidence that the first episode was cured such as a negative blood culture off effective antibacterials for >1 week or absence of symptoms off antibacterials for >1 month	None

	CONFIRMED	PROBABLE
**INFECTIONS (CONTINUED)**		
Toxoplasmosis of brain	Microscopy	A plus B plus C: (A) Symptoms of focal intracranial abnormality or decreased consciousness, (B) brain image consistent with lesion(s) enhanced by contrast, (C) positive toxoplasma serology or responds to treatment clinically or by scan
**NEOPLASMS**		
Cervical carcinoma, invasive	Histology (NOT carcinoma-in-situ)	None
Kaposi sarcoma, (mucocutaneous or visceral)	Histology	Highly typical appearance and persistence for >1 month
Lymphoma, primary, of brain	Histology	Symptoms consistent with lymphoma plus at least one CNS lesion with mass effect plus lack of clinical and radiographic response to at least 2 weeks of treatment for toxoplasmosis
Lymphoma, Hodgkin's	Histology	None
Lymphoma, non-Hodgkin's, all cell types	Histology	None
**NEUROLOGICAL**		
HIV encephalopathy (including AIDS Dementia Complex)	None	Cognitive or motor dysfunction interfering with usual activity, progressive over weeks or months plus no other condition to explain the findings plus brain image obtained and suggests no other causes plus grade 2 or worse impairment in at least 2 domains by NARS (see below) excluding abnormal domains at trial entry. (For persons with abnormal domains at entry worsening by at least two grades meets criteria.)

Abbreviated NARS (Neuropsychiatric AIDS Rating Scale) Grading for HIV Encephalopathy
Adapted from: Price RW, Brew BJ. The AIDS dementia complex. J Infect Dis 1988; 158 (5): 1079-83, and Hughes CP, Berg L, Danziger WL. A new clinical scale for the staging of dementia. Brit J Psych 1982; 140: 566-92.

NARS stage	Cognitive-Behavioral Domains					
	**Orientation**	**Memory**	**Motor**	**Behavior**	**Problem solving**	**Activities of daily living**
**0.5**	fully oriented	complains of memory problems	fully ambulatory slightly slowed movements	normal	has slight mental slowing	slight impairment in business dealings
**1**	fully oriented, may have brief periods of “spaciness"	mild memory problems	balance, co-ordination and handwriting difficulties	more irritable, labile or apathetic, withdrawn	difficulty planning and completing work	can do simple daily tasks, may need prompting
**2**	some disorientation	memory moderately impaired, new learning impaired	ambulatory but may require walking aid	some impulsivity or agitated behaviour	severe impairment, poor social judgement, gets lost easily	needs assistance with ADLs
**3**	frequent disorientation	severe memory loss, only fragments of memory remain	ambulatory with assistance	may have organic psychosis	judgement very poor	cannot live independent ly
**4**	confused and disoriented	virtually no memory	bedridden	mute and unresponsive	no problem solving ability	nearly vegetative

Appendix 2: Template Patient Information Statement and Consent Form

Patient Information Statement and Consent Form

[Insert institutional letterhead]
[name of local institution/s where research is being conducted]

A randomised open-label study comparing the safety and efficacy of ritonavir boosted lopinavir and 2-3N(t)RTI backbone versus ritonavir boosted lopinavir and raltegravir in participants virologically failing first-line NNRTI/2N(t)RTI therapy: the SECOND-LINE study

Invitation

You are invited to participate in a research study to investigate the use of a new antiretroviral drug (Raltegravir) as a key component of therapy following the failure of the first-line anti-HIV regimen (so called 'second-line HIV therapy'). Current guidelines to treat HIV recommend the first types of drugs to be prescribed include drugs from 3 different classes. These classes are called nucleoside reverse transcriptase inhibitors (N(t)RTI), non-nucleoside reverse transcriptase inhibitors (NNRTI) and protease inhibitors (PI). In your case your first set of antiretroviral drugs has included two (2) drugs from the N(t)RTI class and one (1) drug from the NNRTI class. We know from experience that over time these combinations of antiretroviral drugs begin to lose effect in a proportion of people taking them. Eventually in such cases patients need to change to a different set of drugs in order to continue to keep the HIV under control and maintain their health. In general, guidelines recommend that once a person fails treatment with a drug combination with an NNRTI and 2 N(t)RTIs, they should switch therapy to three drugs with a PI combined once again with 2 N(t)RTIs. However, the strength of this recommendation is poor because little research has been done to make sure that this strategy works well. One of the reasons that so little research has been done on that question is that up until recently there haven't been much in the way of alternatives. However, there is now a new anti-HIV drug called raltegravir which is in a new class called the integrase strand-transfer inhibitor (InSTI) class. The evidence to date suggests that raltegravir is powerful and relatively free of unwanted side effects. The availability of this new drug means that we now have an opportunity to see how well it works in patients for whom the 'first-line' therapy is failing.

Raltegravir is the first in class integrase strand-transfer inhibitor licensed for use as part of a combined anti-HIV regimen. At this point in time it is not currently licensed in every country involved in this study.

The study is being conducted by the National Centre in HIV Epidemiology and Clinical Research, University of New South Wales in Australia. The study is part of an international collaborative study.

Before you decide whether or not you wish to participate in this study, it is important for you to understand why the research is being done and what it will involve. Please take the time to read the following information carefully and discuss it with others if you wish.

1. What is the purpose of the study?

The objective of this study is to compare the safety and effectiveness over 96 weeks of two combinations of anti HIV drugs in controlling HIV-infection. Patients enrolled in the study will have been prescribed their first anti-HIV regimen consisting of an NNRTI plus 2N(t)RTIs for a minimum period of 24 weeks, however this treatment is no longer working to control the HIV infection. Participants in the study will change their treatment to either what is currently recommended (LPV/r + 2-3N(t)RTIs) or the new treatment of raltegravir + LPV/r.

2. Why have I been invited to participate in this study?

You have been invited to participate in this study because you have been receiving antiretroviral regimen consisting of an NNRTI plus 2N(t)RTIs for at least 24 weeks and have:

- Failed this therapy according to virological criteria defined by two consecutive (≥7 days apart) HIV RNA results of >500 copies/mL.
- No prior or current exposure to HIV protease inhibitors and/or raltegravir.

3. 'What if I don't want to take part in this study or if I want to withdraw later?'

Participation in this study is voluntary. It is completely up to you whether or not you participate. If you decide not to participate, it will not affect the other treatment you receive now or in the future. Whatever your decision, it will not affect your relationship with the staff caring for you.

New information about the treatment being studied may become available during the course of the study. You will be kept informed of any significant new findings that may affect your willingness to continue in the study.

If you wish to withdraw from the study once it has started, you can do so at any time without having to give a reason.

However, it may not be possible to return your samples to you or withdraw your data from the study results if these have already had your identifying details removed.

4. 'What does this study involve?'

If you agree to participate in this study, you will be asked to sign the Participant Consent Form.
This study will be conducted over 96 weeks.
The treatment being investigated in this study differs from the standard treatment offered in this institution because raltegravir is not currently indicated for this use. However this drug is licensed for the treatment of HIV infection.
This is an open-label study so you will be aware of which treatment you are prescribed.
To ensure you are familiar with the meaning of this terminology and the design of the study the following information may be useful to consider.
The underlying principle for conducting a randomised study is that sometimes the best way of treating patients with a particular condition is not known and so comparisons need to be made between different treatments. To do this, study participants are put into groups and given different treatments, and the results are compared to see whether one treatment is better. To ensure the groups are similar to start with, a computer allocates each study participant into a group randomly, like the flip of a coin. Neither the doctor nor the study participant can decide which treatment the participant receives.

The study aims to enrol 550 subjects from approved sites in Australia, Argentina, Chile, Peru, Mexico, England, Ireland, France, Germany, Malaysia, Singapore, Thailand, Hong Kong, India, New Zealand, South Africa, Israel, and Nigeria.

If you are eligible to enter the study, you will be randomised to receive either:

I. ritonavir boosted lopinavir (LPV/r) 200mg/50mg 4 tabs once daily or 2 tabs twice daily (OR 133.3mg/33.3mg 6 soft gelatin capsules once daily or 3 soft gelatin capsules twice daily) + 2-3N(t)RTIs

II. ritonavir boosted lopinavir (LPV/r) 200mg/50mg 4 tabs once daily or 2 tabs twice daily (OR 133.3mg/33.3mg 6 soft gelatin capsules once daily or 3 soft gelatin capsules twice daily) + raltegravir 400 mg 1 tablet twice daily

You will be required to attend the clinic on 11 occasions over the 96 weeks of the study.

If you agree to participate in this trial, you will be asked to undergo the following procedures:

Initial Visit (to screen for eligibility for study)

- Medical history
- Laboratory blood tests to screen for eligibility and gain a picture of your current state of health. Total amount of blood drawn at this visit will be approximately 30mL (1.5 tablespoons)
- Physical examination by your doctor
- Pregnancy test, if required

Randomisation Visit (on commencement of study drugs)

- Physical examination by your doctor
- Laboratory blood tests for safety and efficacy (fasting). Total amount of blood drawn at this visit will be approximately 75 ml (about 4 tablespoons)
- Pregnancy test, if required
- Measurement of waist and hip circumference, and weight and height
- Questionnaire about your quality of life

Visit at Week 4, 12, 24, 36, 48, 60, 72, 84 and 96

- Physical examination by your doctor
- Laboratory blood tests for safety and efficacy (fasting week 4, 12, 24, 48, 72, 96 and non-fasting weeks 36, 60 and 84). Total amount of blood drawn at this visit will be approximately 70-75mL ml (about 3.5 to 4 tablespoons).
- Pregnancy test, if required
- Assessment for any adverse side-effects of the study drugs
- Measurement of waist and hip circumference, and weight (week 48 and 96 only)
- Questionnaire about your ability to take study medication (week 4, 48 and 96 only)
- Questionnaire about your quality of life (week 48 and 96 only)

Some of the blood collected at all visits will be stored for future testing. It is possible that some of the stored blood may be used as a source of DNA for genetic testing to be done at a later date, but such testing is not so far planned. Prior to any genetic test being undertaken, approval will be sought from your local Research Ethics Committee. In these studies, your DNA may be analysed to see if you have certain types of genes that are responsible for influencing how your body reacts to HIV, the drugs used to treat HIV or infections that can occur in HIV-infected persons. This means the study will only evaluate those genes that have a role in responding to HIV or related infections. If samples are selected for genetic testing, they will not contain any of your personal details such as your name, but only a study ID code. Genetic testing is an optional part of this study. You may refuse genetic testing, which will not affect your participation in this study. If you agree to participate in this part of the study, you have the option of discontinuing at any time. If you change your mind, you must contact research staff and let them know that you do not want your samples used for genetic testing. The results of these genetic tests will be used for research purposes only. Neither you nor your doctor will be given the results from these tests.

At any time during the study, if you have a bad effect from the study medications, you will need to advise your doctor. You may be asked to stop the study medication and be required to come to the clinic for an additional visit(s) for additional exams or lab tests.

Interaction with Concurrent Medications

There can be a risk of serious and/or life threatening side effects when non-study medications are taken with the study drug. For your safety, you must tell the study doctor or nurse about all medications you are taking before you start the study. You must tell any doctor you see during the study about all of the medications you are taking, especially before starting any new medications. This includes any other prescribed medicines, over-the-counter medicines, herbal medicines or supplements. In addition, you must tell the study doctor or nurse before enrolling in any other clinical trials while on this study.

5. 'How is this study being paid for?'

This study is being funded by the National Centre in HIV Epidemiology and Clinical Research (NCHECR), University of New South Wales (UNSW), Merck Sharp & Dohme and Abbott. There is no conflict of interest declared by any of the investigators.

The drugs being used are being provided by their manufacturers: raltegravir by Merck Sharp & Dohme and ritonavir boosted lopinavir by Abbot. All of the money being paid by NCHECR, UNSW to run the trial at your site will be deposited into an account managed by **[*insert hospital/Area Health Service*]**. No money is paid directly to individual researchers.

6. 'Are there risks to me in taking part in this study?'

All medical procedures involve some risk of injury. In addition, there may be risks associated with this study that are presently unknown or unforeseeable.
As part of this study, you will have your blood drawn. This procedure may be uncomfortable but rarely results in any significant problems. Side effects that have been noted with drawing blood include feeling light-headed or faint, fainting, pain and bruising at the needle stick site.
Some of the side effects of the drugs in the study are listed below. Please note that this list does not include all the side effects seen with these drugs. This list includes the more common or serious side effects. If you have questions concerning any additional side effects, please ask the study doctor or nurse at your site.

**Frequency**	**Raltegravir**	**Kaletra/Aluvia**
Very Rare (<1%)	Irritation of the stomach lining, liver problems, herpes simplex, decrease in kidney function, low blood counts, increase in blood glucose	Decreased sex drive
Rare# (≥1% <2%)	Weakness, tiredness, nausea, diarrhoea	Anorexia, muscular pain, abnormal sensations such as burning or tingling, depression, rash
Uncommon# (≥2%<10%)	Headache, diarrhoea	Abdominal pain, weakness, headache, nausea, vomiting, upset stomach, flatulence, insomnia
Common* (>10%)	Headache, nausea, diarrhoea, fever, tiredness, vomiting, illness caused by an infection involving the nose or throat	Diarrhoea

# moderate intensity (discomfort enough to cause interference with usual activity) to severe intensity (incapacitating with inability to work or do usual activity)
* all intensities

Some adverse events have been reported but it remains unknown if they were related to raltegravir and are considered rare, these include: creatinine kinase elevations and muscle disease.

Pregnancy
Because of the unknown effects of raltegravir on the unborn child, women should avoid becoming pregnant during the course of this trial. If you do become pregnant during the study you should tell your study doctor immediately. Your study doctor will discuss the options with you at that time and will follow your progress until your baby is born.

You should speak to the study doctor about the need to avoid pregnancy during this study.

7. What happens if I suffer injury or complications as a result of the study?'

If you suffer any injuries or complications as a result of this study, you should contact the study doctor as soon as possible, who will assist you in arranging appropriate medical treatment.

If you suffer any adverse experience resulting directly from the study drug/procedure, compensation will be provided for the reasonable costs of medical treatment to the extent such costs are not covered by your medical insurance or government health schemes. Compensation will be provided in accordance with the principles of the national compensation scheme of the country in which the study is performed and the incident occurs will be adhered to. If no national scheme exists the Clinical Trial Compensation Guidelines issued by Medicines Australia will be adhered to.

8. 'Will I benefit from the study?'

Information learned from this study may improve future treatments of HIV infection however it may not directly benefit you.

9. 'Will taking part in this study cost me anything, and will I be paid?

The only specific cost in this study will be the travel costs for attending the clinic on the 11 occasions for study specific visits. It is hoped you can arrange for most of these visits to tie in with your normal three monthly visits with your doctor.

The NCHECR, UNSW will cover any cost associated with provision of the study drugs.

10. 'What will happen to my blood sample after it has been used?'

This blood will be stored for current and future medical research that relates to the treatment of HIV. Not all potentially beneficial future research can be known at any one time, as the need for future research is determined by ongoing developments in the field indefinitely for future research. The Human Research Ethics Committee will determine whether, or not, your consent should be obtained at that time for a particular research project.

11. 'How will my confidentiality be protected?'

Of the people treating you, only the study doctor and research team involved in your care will know whether or not you are participating in this study. Any identifiable information that is collected about you in connection with this study will remain confidential and will be disclosed only with your permission, or except as required by law. Only the researchers at your site will have access to your details and results that will be held securely at ***[insert name of institution].***

12. 'What happens with the results?'

If you give us your permission by signing the consent document, we may discuss/publish the results e.g. with the sponsor for monitoring purposes, the HREC for monitoring purposes, peer-reviewed medical and scientific journals, presentation at conferences or other professional forums.
In any publication, information will be provided in such a way that you cannot be identified. Results of the study will be provided to you, if you wish.

13. 'What happens to my treatment when the study is finished?'

Study drug will be provided for a maximum of 3 years as part of the protocol. If the study drugs are not available locally after that time then the decision will be made in consultation between you and your treating doctor about the most appropriate treatment.

14. 'What should I do if I want to discuss this study further before I decide?'

When you have read this information, the researcher ***[insert name]*** will discuss it with you and any queries you may have. If you would like to know more at any stage, please do not hesitate to contact him/her on **[insert *number – or other if different*].**

15. 'Who should I contact if I have concerns about the conduct of this study?'

This study has been approved by **[insert *HREC name*]**. If you have any questions about your rights as a research participant, please contact **[*insert HREC contact details*]** and quote **[insert *HREC project number*].**

Thank you for taking the time to consider this study.
If you wish to take part in it, please sign the attached consent form.
This information sheet is for you to keep.

[Insert institutional letterhead]
[name of local institution/s where research is being conducted]

A randomised open-label study comparing the safety and efficacy of ritonavir boosted lopinavir and 2-3N(t)RTI backbone versus ritonavir boosted lopinavir and raltegravir in participants virologically failing first-line NNRTI/2N(t)RTI therapy: the SECOND-LINE study

1. I,..
   of..
   agree to participate as a subject in the study described in the participant information statement attached to this form.

2. I acknowledge that I have read the participant information statement, which explains why I have been selected, the aims of the study and the nature and the possible risks of the investigation, and the statement has been explained to me to my satisfaction.

3. Before signing this consent form, I have been given the opportunity of asking any questions relating to any possible physical and mental harm I might suffer as a result of my participation and I have received satisfactory answers.

4. I understand that I can withdraw from the study at any time without prejudice to my relationship to the ***([insert or delete as necessary] University [name] and the ......Hospital, Research Institute).***

5. I agree that research data gathered from the results of the study may be published, provided that I cannot be identified.

6. I understand that if I have any questions relating to my participation in this research, I may contact Dr ....on telephone................, who will be happy to answer them.

7. I agree to have my blood samples used for genetic testing ________(Patient initials)
   I do not agree to have my blood samples used for genetic testing______(Patient initials)

8. I acknowledge receipt of a copy of this Consent Form and the Participant Information Statement.

Complaints may be directed to the, Research Governance Officer, ***[insert local details]***

Signature of subject **Please PRINT name** **Date**

____

Signature of witness **Please PRINT name** **Date**

____

Signature of investigator **Please PRINT name** **Date**

____

[Insert institutional letterhead]
[name of local institution/s where research is being conducted]

A randomised open-label study comparing the safety and efficacy of ritonavir boosted lopinavir and 2-3N(t)RTI backbone versus ritonavir boosted lopinavir and raltegravir in participants virologically failing first-line NNRTI/2N(t)RTI therapy: the SECOND-LINE study

REVOCATION OF CONSENT

I hereby wish to **WITHDRAW** my consent to participate in the study described above and understand that such withdrawal **WILL NOT** jeopardise any treatment or my relationship with the (***University...[insert name of university], Hospital or my medical attendants)***.

Signature____Date_____________

Please PRINT Name________________________________

The section for Revocation of Consent should be forwarded to ***(INSERT name and address of Principle Investigator).***

Appendix 3: Diagnostic Criteria for Serious Non-AIDS Events

ACUTE MYOCARDIAL INFARCTION

A. Rise and/or fall of cardiac biomarkers (preferably troponin), with at least one value above 99th percentile of upper reference limit (URL)

B. Occurrence of a compatible clinical syndrome, including symptoms (such as chest pain) consistent with myocardial ischemia

C. ECG changes indicative of new ischemia (new ST-changes or new left bundle branch block [LBBB]), or development of pathological Q waves on the ECG

D. Imaging evidence of new loss of viable myocardium or new regional wall motion abnormality

E. Sudden unexpected cardiac death involving cardiac arrest before biomarkers are obtained or before a time when biomarkers appear, along with (1) new ST-changes or new LBB, or (2) evidence of fresh thrombus on coronary angiography or at autopsy

F. In participants with percutaneous coronary interventions and normal baseline troponin, increases in troponin of three times the 99th percentile of URL

G. In participants with coronary artery bypass grafting and normal baseline troponin, increases in troponin of five times the 99th percentile of URL PLUS at least one of the following: (1) new pathological Q-waves or new LBBB, (2) angiographically documented new graft or native artery occlusion, or (3) imaging evidence of new loss of viable myocardium

H. Pathologic findings of acute myocardial infarction (including acute MI demonstrated as the cause of death on autopsy)

I. Development of 1) evolving new Q waves, or 2) evolving ST elevation, preferably based on at least 2 ECGs taken during the same hospital admission

J. In participants with coronary artery bypass grafting and normal baseline troponin, increases in troponin of five times the 99th percentile of URL

Confirmed: One of the following 5 criteria (adapted from 2007 Universal Definition of Myocardial Infarction):
1. A + (B or C or D)
2. E
3. F
4. G
5. H

Probable: B+I or J

CONGESTIVE HEART FAILURE

A. Clinical signs and symptoms compatible with left or right sided heart failure (e.g., paroxysmal nocturnal dyspnea, rales or S3 on auscultation, jugular venous distention) without an alternative explanation

B. Hemodynamic measurements, radionucleotide ventriculography, echocardiogram, cardiac catheterization, or multiple gated acquisition scan showing a decreased ejection fraction of < 45%

C. Echocardiogram, cardiac catheterization or other studies showing evidence of increased left atrial pressure or right heart failure

D. Elevated levels of Brain Natriuretic Peptide (BNP) or pro-BNP

E. Chest x-ray or other imaging study showing evidence of congestive heart failure, including cardiac enlargement

F. Documentation of treatment for congestive heart failure

Confirmed: (A+B) or (A+C) or (A+D)

Probable: A+E+F

CORONARY ARTERY DISEASE REQUIRING REQUIRING DRUG TREATMENT

Confirmed: A written report in the medical record documenting: (A) myocardial ischemia and/or coronary artery disease, AND (B) use of medications given to treat or prevent angina (e.g., nitrates, beta blockers, calcium channel blockers)

Probable: Not applicable

CORONARY REVASCULARISATION

Confirmed: A written report in the medical record or hospital discharge summary from the hospitalization during which the procedure was performed for treatment of coronary artery disease, including: coronary artery bypass graft, coronary artery stent implant, coronary artherectomy, and percutaneous transluminal angioplasty

Probable: Not applicable

DECOMPENSATED LIVER DISEASE

A. Histologic, radiographic, or ultrasound evidence of cirrhosis, as documented by one of the following:

1. Histologic evidence of cirrhosis obtained by liver biopsy or autopsy
2. MRI or CT consistent with cirrhosis

3. A positive result on transient elastography (FibroScan) or other ultrasound imaging consistent with cirrhosis

B. Clinical evidence of decompensation, as documented by one of the following, and without an alternative explanation:

1. Ascites
2. Hepatic encephalopathy
3. Bleeding from gastric or esophageal varices
4. Spontaneous bacterial peritonitis

Confirmed: A+B

Probable: B

DEEP VEIN THROMBOSIS

A. Diagnosis of deep vein thrombosis (DVT) by contrast venography, helical computed tomography, MRI, or ultrasonography other comparable imaging techniques

B. An elevated D-dimer test OR abnormal plethysmography

C. A score on the Wells Clinical Prediction Rule for DVT of >=3 points

D. Absence of alternative diagnosis as likely or greater than that of deep venous thrombosis

Wells Clinical Prediction Rule for DVT

One point for each of the following:

• Active cancer (treatment ongoing or within previous 6 months, or palliative)
• Paralysis, paresis, or plaster immobilization of lower extremities
• Recently bedridden for more than 3 days, or major surgery, within 4 weeks
• Localized tenderness along distribution of the deep venous system
• Entire leg swollen
• Calf swelling by more than 3 cm when compared with the asymptomatic leg (measured 10 cm below tibial tuberosity)
• Pitting edema (greater in the symptomatic leg)
• Collateral superficial veins (non-varicose)

(Adapted from: Wells PS et al. Lancet 1997;350:1796)

Confirmed: A

Probable: B+C+D

DIABETES MELLITUS

A. Symptoms of diabetes plus casual plasma glucose concentration >= 200 mg/dL (11.1 mmol/L). (Casual is defined as anytime of day without regard to time since last meal. The classic symptoms of diabetes include polyuria and polydipsia).

B. Fasting plasma glucose >= 126 mg/dL (7.0 mmol/L). (Fasting is defined as no caloric intake for at least 8 hours).

C. 2-hour post-load glucose >= 200 mg/dL (11.1 mmol/L) during an oral glucose tolerance test. (The test should be performed as described by WHO, using glucose load containing the equivalent of 75 g anhydrous glucose dissolved in water).

Confirmed: A or B or C

Probable: none

END-STAGE RENAL DISEASE

A. Hemodialysis or peritoneal dialysis for a period of at least one month, documented in a clinical note

B. A kidney transplant, documented in a clinical note

Confirmed: A or B

Probable: Not applicable

NON-AIDS-DEFINING CANCER

A. Diagnosis of cancer other than lymphoma, Kaposi's sarcoma (KS), or invasive cervical cancer in an autopsy report

B. Diagnosis of cancer other than lymphoma, KS, or invasive cervical cancer in a pathology report that established the diagnosis

C. Diagnosis of cancer other than lymphoma, KS, or invasive cervical cancer in a hospital discharge summary or consultation note from the hospitalization or clinic visit during which the diagnosis was established

Confirmed: A or B

Probable: C

PERIPHERAL ARTERIAL DISEASE

A. Compatible clinical signs and symptoms (e.g., intermittent claudication, femoral bruit, decreased peripheral pulses, change in color or temperature of limb suggesting peripheral arterial disease)

B. Positive results on diagnostic imaging studies (e.g., Doppler ultrasound, contrast arteriography, MRI arteriography)

C. Ankle Brachial Pressure Index < 0.90 in non-diabetics

D. A procedure report, hospital discharge summary, or other medical record from the hospitalization during which the procedure was performed documenting an invasive procedure for treatment of peripheral arterial disease (e.g. percutaneous transluminal angioplasty, endovascular procedures, or vascular surgery), or a consultation note documenting the occurrence of the procedure

Confirmed: (A+B) or (A+C) or D

Probable: A

PULMONARY EMBOLISM

A. Symptoms compatible with pulmonary embolism, such as shortness of breath, chest pain, or hemoptysis

B. Results consistent with a diagnosis of pulmonary embolism on pulmonary angiography, helical CT, ventilation-perfusion scan or other comparable imaging studies

C. A diagnosis of pulmonary embolism on autopsy

D. Results consistent with a diagnosis of deep venous thrombosis on venography, ultrasound, or other comparable imaging studies

E. A chest x-ray which, if performed, does not suggest an alternative etiology for the symptoms described in criteria A

Confirmed: (A+B) or C

Probable: A+D+E

STROKE

A. Acute onset with a clinically compatible course, including unequivocal objective findings of a localizing neurologic deficit

B. CT or MRI compatible with diagnosis of stroke and current neurologic signs and symptoms

C. Stroke diagnosed as cause of death at autopsy

D. Positive lumbar puncture compatible with subarachnoid hemorrhage

E. Death certificate or death note from medical record listing stroke as cause of death

Confirmed: (A+B) or C

Probable: (A+D) or (A+E)
