# Supplementary material for: Raltegravir Non-Inferior to Nucleoside Based Regimens in SECOND-LINE Therapy with Lopinavir/Ritonavir over 96 Weeks: A Randomised Open Label Study for the Treatment Of HIV-1 Infection
Source: PLoS One. 2015 Feb 27;10(2):e0118228. doi: 10.1371/journal.pone.0118228 (PMC4344344; doi:10.1371/journal.pone.0118228)
Supplement: S1 Ethics — (DOCX) [file pone.0118228.s003.docx]

| **Ethics Committees** |
| --- |
| The Alfred Hospital Ethics Committee: Australia |
| Comite de etica CAICI-CIAP: Argentina |
| National Research Ethics Service: UK |
| University of Witwatersrand Human Research Ethics Committee: South Africa |
| Minesterio De Salud Sevicio De Salud M. Central: Chile |
| Univertsity of Capetown Human Research Ethics Committee: South Africa |
| Comite de Protection des Personnes Ile de France IV: France |
| Geschaftsfuhrung der Ethik-Kommission: Germany |
| Kowloon Central/ Kowloon East Research Ethics Committee: Hong Kong |
| Chest Research Foundation Independent Ethics Committee: India |
| Mater Misericordiae University Hospital: Ireland |
| Helsinki Committee Rambam Health Care Campus: Helsinki |
| Ethics Committee University of the Free State: South Africa |
| Western Zone- Human Research Ethics Committee: Australia |
| Medical Research & Ethics Committee Ministry of Health Malaysia: Malaysia |
| Jawatankuasa Etika Perubatan: Malaysia |
| Comite ed Etica en Investigacion: Mexico |
| Health and Disability Ethics Committees: New Zealand |
| Jos University Institutional Health Research Ethical Committee: Nigeria |
| Es Salud Ethics Committee: Peru |
| National Healthcare Group: Singapore |
| St Vincent's Hospital Human Research Ethics Committee: Australia |
| National Taiwan University Hospital Research Ethics Committee: Taiwan |
| Chang Mai University Human Experimentation Committee: Thailand |
| Chulalongkorn University Institutional Review Borad: Thailand |
| Khon Kaen University Institutional Review Board: Thailand |
| Khon Kaen Hospital Institutional Review Board: Thailand |
| YRG Care Institutional Review Board: India |
